# PDK4 drives abdominal aortic aneurysm by promoting smooth muscle cell metabolic reprogramming and NLRP3-mediated pyroptosis

**DOI:** 10.1038/s41467-026-71610-w

**Published:** 2026-04-11

**Authors:** Li Zhao, Xuefeng Lin, Zhengqiang Zhu, Ranxin Liu, Lingna Zhao, Xuekun Wu, Mengru Zheng, Rihua Huang, Pengyu Zhou, Fangze Huang, Deshen Liu, Chuanjie Niu, Xiaoxia He, Zean Wang, Xin Li, Jiale Li, Shengping He, Jun Lu, Shaoyi Zheng, Jiaguo Zhou, Qinbao Peng, Xiu Liu

**Affiliations:** 1 Department of Cardiovascular Surgery, Nanfang Hospital, Southern Medical University, Guangzhou, China; 2 Stanford Cardiovascular Institute, Stanford University School of Medicine, Stanford, CA USA; 3 Department of Cardiology, The First Affiliated Hospital, Sun Yat-Sen University, Guangzhou, China; 4 Department of Cardiac Surgery, Guangdong Provincial People’s Hospital (Guangdong Academy of Medical Sciences), Southern Medical University, Guangzhou, China; 5 Department of Pharmacology, Cardiac and Cerebral Vascular Research Center, Zhongshan School of Medicine, Sun Yat-sen University, Guangzhou, China; 6 Guangdong Provincial Key Laboratory of Single-cell and Extracellular Vesicles, Southern Medical University, Guangzhou, China

**Keywords:** Aneurysm, Chronic inflammation

## Abstract

Abdominal aortic aneurysm (AAA) is a progressive dilation of the abdominal aorta that can rupture and cause catastrophic internal bleeding, yet the mechanisms driving AAA remain poorly understood. Here we show that pyruvate dehydrogenase kinase 4 (PDK4), a key metabolic regulator, is upregulated in human and mouse AAA tissues. Deletion of *Pdk4* in vascular smooth muscle cells (VSMCs) significantly reduces AAA formation in male mice. Mechanistically, PDK4 promotes metabolic reprogramming in VSMCs, disrupts mitochondrial respiration, and activates the NLRP3 inflammasome and pyroptosis, thereby exacerbating vascular inflammation and AAA progression. Genetic deletion of *Pdk4* in VSMCs or pharmacological inhibition of NLRP3 attenuates AAA development in mice. These findings identify PDK4 as a driver of AAA and suggest that targeting PDK4 may represent a therapeutic strategy for this life-threatening disease.

## Abstract (web summary)

Abdominal aortic aneurysm (AAA) is a life-threatening condition, but its underlying mechanisms are unclear. Here, the authors show that PDK4 contributes to abdominal aortic aneurysm by promoting metabolic reprogramming in vascular smooth muscle cells, leading to activation of NLRP3.

## Introduction

Abdominal aortic aneurysm (AAA) is a life-threatening condition characterized by localized dilation of the abdominal aorta, posing a significant risk of rupture and fatal hemorrhage^1^. AAA pathogenesis is complex and involves a combination of genetic, environmental, and molecular factors. Clinically, AAA is typically asymptomatic until rupture; therefore, early detection and intervention are crucial for patient survival. Despite advances in surgical interventions such as endovascular aneurysm repair (EVAR) and open surgical repair, the molecular mechanisms underlying AAA development and progression remain incompletely understood^1^. Therefore, further research is needed to elucidate the pathogenesis of AAA and optimize therapeutic strategies.

Vascular smooth muscle cells (VSMCs) play a critical role in maintaining vascular homeostasis and function^2^. VSMCs exhibit remarkable plasticity and can switch between contractile and synthetic phenotypes in response to various stimuli. VSMCs in AAA undergo a phenotypic switch from a contractile to a synthetic phenotype, characterized by increased proliferation, migration, and extracellular matrix production^3^. This phenotypic switch is believed to contribute to the pathological remodeling of the aortic wall observed in AAA.

Mitochondrial dysfunction in VSMCs has emerged as a critical factor in the pathogenesis of various cardiovascular diseases, including AAA^4^. Mitochondria are central to cellular energy metabolism, reactive oxygen species (ROS) production, and apoptotic pathway regulation^5^. Dysfunctional mitochondria in VSMCs can result in impaired oxidative phosphorylation, elevated ROS levels, and mitochondrial DNA damage, all of which have been implicated in AAA pathogenesis^6^. Oxidative stress and energy deficiency can trigger a cascade of pathological events, including metabolic reprogramming and inflammatory responses^7^.

The pyruvate dehydrogenase complex (PDC) is a key regulator of cellular metabolism and plays a crucial role in regulating mitochondria-derived metabolites. PDC catalyzes the conversion of pyruvate, coenzyme A, and nicotinamide adenine dinucleotide (NAD^+^) into acetyl-CoA, reduced nicotinamide adenine dinucleotide (NADH), and CO2, thereby linking glycolysis to the tricarboxylic acid cycle^8^. PDC is inhibited via phosphorylation by pyruvate dehydrogenase kinase (PDK), which has several isoforms (PDK1, PDK2, PDK3, and PDK4)^9^. Increased PDK levels contribute to the pathogenesis of atherosclerosis, heart failure, and various other conditions^10,11^. By analyzing single-cell sequencing data from AAA tissues, we found that PDK4, but not PDK1, PDK2, or PDK3, exhibited the most significant differential expression in VSMCs. Therefore, PDK4 was selected for further investigation. As a key regulator of cellular metabolism, PDK4 suppresses PDC activity and promotes a metabolic shift from oxidative phosphorylation to glycolysis. In addition, PDK4 regulates glucose metabolism and contributes to vascular inflammation, vascular calcification, atherogenesis, and plaque instability^11,12^. These functions highlight PDK4’s potential as a therapeutic target for treating atherosclerosis and other cardiovascular diseases. However, the role of PDK4-mediated mitochondrial respiratory alterations in AAA, as well as the underlying molecular mechanisms, remains unclear.

Therefore, this study investigated the role of PDK4 in regulating mitochondrial respiration in VSMCs to improve our understanding of AAA pathogenesis and identify potential therapeutic targets for AAA. Given the effects of PDK4 on mitochondrial respiration, excessive ROS production, and inflammation12, we hypothesized that reducing PDK4 levels would attenuate AAA progression.

## Results

### PDK4 expression is upregulated in AAA tissues in humans and mice

**Fig. 1:**
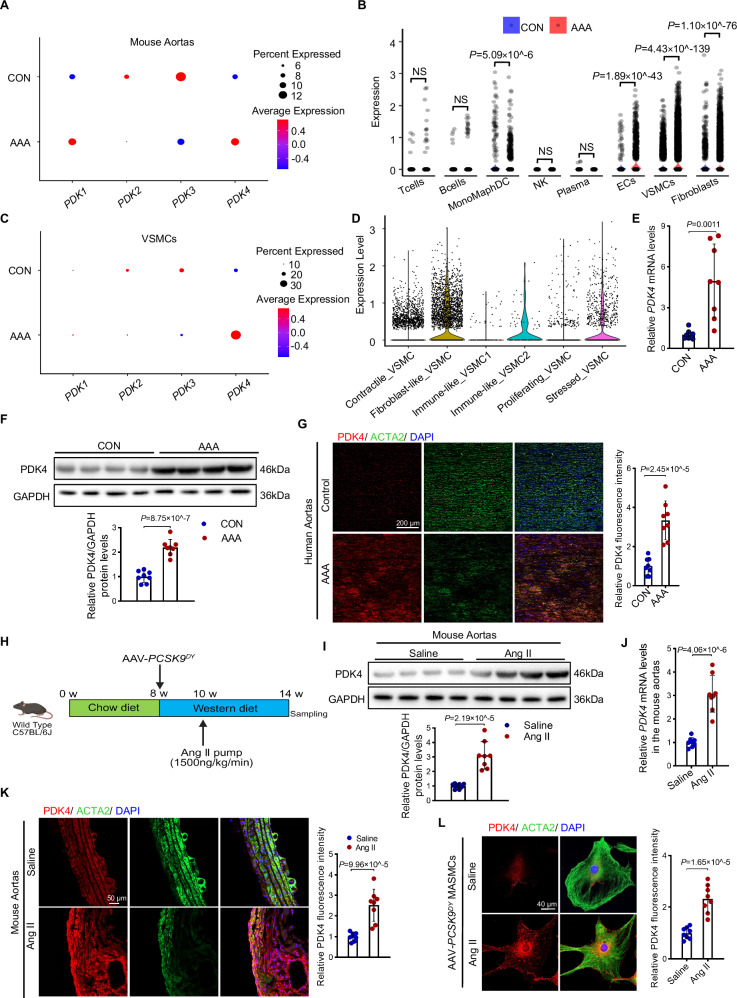
PDK4 is upregulated in human and mouse AAA. **A** Dot plot showing the relative expression levels of *PDK1*, *PDK2*, *PDK3*, and *PDK4* in control and AAA groups (Gene Expression Omnibus accession no. GSE239620). **B** Violin plots showing *PDK4* expression across major cell types in the control and AAA groups. **C** Dot plot indicating the relative expression levels of *PDK1*, *PDK2*, *PDK3*, and *PDK4* in VSMCs between control and AAA groups. **D** Violin plot of *PDK4* expression levels across six VSMC subclusters. **E**, **F** Relative PDK4 mRNA and protein levels were measured by RT-qPCR and Western blot analysis in the abdominal aortas of patients with abdominal aortic aneurysm (AAA) and adjacent non-aneurysmal segments (control) (*n* = 8 patients). **G** PDK4 and ACTA2 immunofluorescence in the aortas of patients with AAA and controls (*n* = 8 patients). **H** Schematic representation of the AAV-*PCSK9*^*DY*^/Ang II-induced AAA model in wild-type mice. Created with BioRender. Liu (2026) https://BioRender.com/xax8hm0. **I**, **J** Relative PDK4 protein and mRNA levels were measured by RT-qPCR and Western blot analysis in the abdominal aortas of mice treated with AAV-*PCSK9*^*DY*^/Ang II for 28 days or AAV-*PCSK9*^*DY*^/saline for 28 days (*n* = 8 biological replicates). **K** PDK4 and ACTA2 immunofluorescence in suprarenal abdominal aortas from mice treated with AAV-*PCSK9*^*DY*^/Ang II for 28 days or AAV-*PCSK9*^*DY*^/saline for 28 days (*n* = 8 biological replicates). **L** PDK4 and ACTA2 immunofluorescence in mouse aortic smooth muscle cells (MASMCs) isolated from AAV-*PCSK9*^*DY*^ mice treated with Ang II or saline for 28 days (*n* = 8 biological replicates). Data are presented as mean ± standard deviation (SD). **B** Two-sided Mann–Whitney *U*-test (exact method). **E**–**G**, **I**–**L** Two-sided unpaired Student’s *t*-test. Source data are provided as a Source Data file.

We reanalyzed single-cell RNA sequencing data from aortic tissues of AAA and control mice in the GEO database (GSE239620) to assess the association between PDK4 expression and abdominal aortic aneurysm. Eight major cell types were identified using classical markers (Fig. S1A). Distinct gene expression profiles characterized individual cell types (Fig. S1B). The results indicated that PDK4 expression is predominantly highest in VSMCs, followed by fibroblasts and endothelial cells (ECs) (Fig. S1C,D). Among the PDK isoforms, PDK4, but not PDK1, PDK2, or PDK3, showed the most significant differential expression in AAA tissues (Fig. 1A). This difference was most pronounced in VSMCs when comparing AAA and control groups (Fig. 1B). Consistently, PDK4, but not PDK1, PDK2, or PDK3, exhibited the most marked differential expression in VSMCs from AAA tissues (Fig. 1C). Analysis of VSMC clusters identified six distinct subpopulations: Contractile_VSMC, Fibroblast-like_VSMC, Immune-like_VSMC1, Immune-like_VSMC2, Proliferating_VSMC, and Stressed_VSMC (Fig. S1E,F). *PDK4* expression was predominantly elevated in the Fibroblast-like_VSMC subpopulation (Fig. 1D).

Consistent with the single-cell RNA sequencing results, Western blot analysis showed that PDK1, PDK2, and PDK3 levels were not significantly altered in AAA tissues from mice treated with AAV-*PCSK9*^*DY*^/ angiotensin II (Ang II) for 28 days compared with those from mice treated with AAV-*PCSK9*^*DY*^/saline for 28 days, whereas PDK4 was markedly upregulated (Fig. S1G, H). Recent studies have indicated that activation of the PI3K/AKT pathway suppresses PDK4 expression^13^. Given that the PI3K/AKT pathway has been reported to play a key role in AAA^14^, we used the PI3K inhibitor LY294002 to examine PDK4 expression. Our results showed that LY294002 increased the mRNA levels of *PDK4* (Fig. S1I). Furthermore, Western blot and reverse transcription-quantitative polymerase chain reaction (RT-qPCR) demonstrated that *PDK4* mRNA and protein levels were elevated in human AAA tissues obtained during abdominal aortic replacement surgery compared with control aortic tissues from non-aneurysmal segments of the same patients (Fig. 1E, F). Immunofluorescence further showed lower ACTA2 expression and higher PDK4 expression in human AAA tissues than in control tissues (Fig. 1G).

We established an in vivo AAA mouse model by inducing hyperlipidemia and hypertension^15^. Hyperlipidemia was induced using an adeno-associated virus (AAV) encoding the PCSK9 D377Y (Asp374-to-Tyr) mutant (AAV-*PCSK9*^*DY*^), whereas hypertension was induced by Ang II infusion^16^. Wild-type mice were used to generate the AAV-*PCSK9*^*DY*^/Ang II-induced AAA model (Fig. 1H). Western blot and RT-qPCR analyses showed that PDK4 mRNA and protein levels were increased in AAA tissues from mice treated with AAV-*PCSK9*^*DY*^/Ang II for 28 days compared to those treated with AAV-*PCSK9*^*DY*^/saline for 28 days (Fig. 1I-J). Immunofluorescence analysis further showed that suprarenal abdominal aortic tissues from mice treated with AAV-*PCSK9*^*DY*^/Ang II for 28 days exhibited reduced ACTA2 expression and increased PDK4 expression compared with tissues from mice treated with AAV-*PCSK9*^*DY*^ /saline for 28 days (Fig. 1K)

Moreover, we isolated mouse aortic smooth muscle cells (MASMCs) from abdominal aortas of AAV-*PCSK9*^*DY*^-treated wild-type mice or *Apoe*^−^^*/*^^−^ mice infused with saline or Ang II for 28 days (Fig. S2A). PDK4 mRNA and protein levels were elevated in MASMCs isolated from AAV-*PCSK9*^*DY*^ or *Apoe*^−^^*/*^^−^ mice infused with Ang II for 28 days (Fig. S2B–E). Immunofluorescence analysis showed that MASMCs isolated from AAV-*PCSK9*^*DY*^ or *Apoe*^−^^*/*^^−^ mice infused with Ang II for 28 days exhibited decreased ACTA2 and elevated PDK4 expression compared to those from AAV-*PCSK9*^*DY*^ or *Apoe*^−^^*/*^^−^ mice infused with saline for 28 days (Fig. 1L and Fig. S2F). Time-course analyses revealed that PDK4 expression was markedly increased within just 3 days in AAA tissues induced by Ang II (Fig. S2G, H). Collectively, these results indicated that PDK4 is upregulated in human and mouse AAA and may play an essential role in AAA.

### VSMC-specific deletion of PDK4 mitigates AAA development in mice

**Fig. 2:**
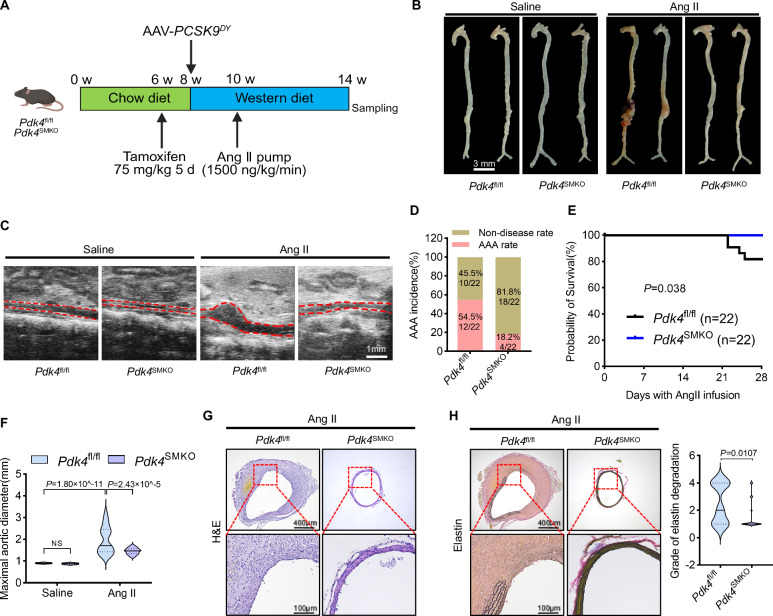
VSMC-PDK4 deficiency mitigates AAA in mice. **A**–**H** Eight-week-old male *Pdk4*^fl/fl^ and *Pdk4*^SMKO^ mice were injected via the tail vein with AAV-*PCSK9*^*DY*^ and fed a Western-type diet. After 2 weeks, mice were infused with Ang II (1500 ng/kg/min) or saline for an additional 4 weeks (*n* = 22 for *Pdk4*^fl/fl^ and *Pdk4*^SMKO^). **A** Schematic representation of the AAV-*PCSK9*^*DY*^/Ang II model established in *Pdk4*^fl/fl^ and *Pdk4*^SMKO^ mice. Created with BioRender. Liu (2026) https://BioRender.com/shehazp. **B** Representative macroscopic images of mouse aortas. **C** Representative ultrasound images of the abdominal aorta (scale bar = 1 mm). **D** Abdominal aortic aneurysm (AAA) incidence. **E** Survival curves were analyzed using the Kaplan–Meier method and compared using log-rank tests. **F** Maximum diameter of suprarenal abdominal aortas measured by B-mode ultrasound imaging (saline group: n = 22 for *Pdk4*^fl/fl^; *n* = 22 for *Pdk4*^SMKO^; Ang II group: *n* = 18 for *Pdk4*^fl/fl^; *n* = 22 for *Pdk4*^SMKO^). **G**, **H** Representative hematoxylin and eosin (H&E) and Verhoeff–Van Gieson staining for elastin of the mouse suprarenal abdominal aorta. The grade of elastin degradation in the aortic wall (*n* = 18 for *Pdk4*^fl/fl^; *n* = 22 for *Pdk4*^SMKO^). Data are presented as mean ± SD. **F** Two-way ANOVA followed by Tukey’s multiple-comparisons test. **H** Two-sided Mann–Whitney *U*-test (exact method). Source data are provided as a Source Data file.

We generated VSMC-specific *Pdk4* knockout mice (*Myh11*-creERT2-Cre/*Pdk4*^fl/fl^), referred to as *Pdk4*^SMKO^ mice, to investigate the role of PDK4 in AAA pathology (Fig. S3A, B). PDK4 deletion in VSMCs from *Pdk4*^SMKO^ mice was confirmed by immunofluorescence and Western blot (Fig. S3C, D). The AAV-*PCSK9*^*DY*^/Ang II-induced AAA model was established in *Pdk4*^fl/fl^ and *Pdk4*^SMKO^ mice (Fig. 2A). No differences were observed in aortic morphology between the AAV-*PCSK9*^*DY*^/saline-infused *Pdk4*^SMKO^ and *Pdk4*^fl/fl^ mice (Fig. 2B). Upon AAV-*PCSK9*^*DY*^/Ang II treatment, *Pdk4*^SMKO^ mice exhibited markedly reduced AAA development, with lower mortality (0% vs. 18.2%) and AAA incidence (18.2% vs. 54.5%) than *Pdk4*^fl/fl^ mice (Fig. 2B–E). Moreover, no differences were observed in maximum abdominal aortic diameter between the AAV-*PCSK9*^*DY*^/saline-infused *Pdk4*^SMKO^ and *Pdk4*^fl/fl^ mice (Fig. 2F). However, after 28 days of AAV-*PCSK9*^*DY*^/Ang II treatment, *Pdk4*^SMKO^ mice showed a significant reduction in maximum aortic diameter and elastin degradation compared with *Pdk4*^fl/fl^ mice (Fig. 2F–H). Blood pressure, body weight, plasma lipid levels, and aortic root plaque areas were comparable between *Pdk4*^fl/fl^ and *Pdk4*^SMKO^ mice after 28 days of AAV-*PCSK9*^*DY*^/Ang II treatment (Fig. S3E–I). SNP genotyping confirmed that *Pdk4*^SMKO^ and *Pdk4*^fl/fl^ mice shared an identical C57BL/6J genetic background (Supplementary Table 1). These findings suggest that VSMC-specific PDK4 deficiency plays a protective role against AAA development.

### VSMC-specific PDK4 overexpression aggravates AAA in mice

**Fig. 3:**
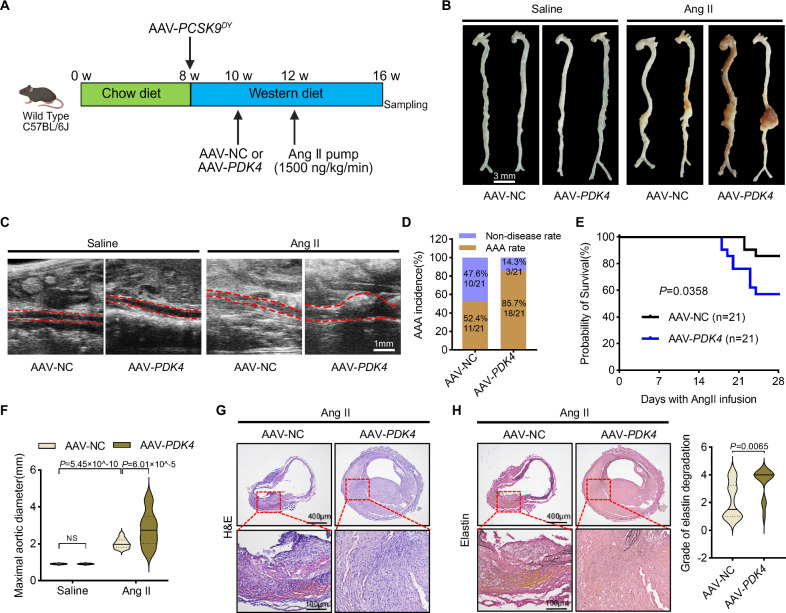
VSMC-PDK4 overexpression aggravates AAA in mice. **A**–**H** Eight-week-old wild-type male mice were injected via the tail vein with AAV-*PCSK9*^*DY*^ and fed a Western-type diet. Two weeks after the AAV-*PCSK9*^*DY*^ injection, the mice were injected with AAV-NC or AAV-*PDK4*. After two weeks, they were infused with Ang II (1500 ng/kg/min) or saline for another four weeks (*n* = 21 for AAV-NC and *n* = 21 for AAV-*PDK4*). **A** Schematic of the AAV-*PCSK9*^*DY*^/Ang II model establishment in AAV-NC and AAV-*PDK4* mice. Created with BioRender. Liu (2026) https://BioRender.com/oqbn39n. **B** Macrographs of mouse aorta. **C** Representative ultrasound images of the abdominal aorta (scale bar = 1 mm). **D** Abdominal aortic aneurysm (AAA) incidence. **E** Survival curves were analyzed using the Kaplan–Meier method and compared using log-rank tests. **F** Measurement of the maximum diameter of suprarenal abdominal aortas was performed using B-mode ultrasound imaging (saline group: *n* = 21 for AAV-NC; *n* = 21 for AAV-*PDK4*; Ang II group: *n* = 18 for AAV-NC; *n* = 12 for AAV-*PDK4*). **G**, **H** Representative hematoxylin and eosin (H&E) and Verhoeff–Van Gieson staining for elastin of the mouse suprarenal abdominal aorta. Grade of elastin degradation in the aortic wall (*n* = 18 for AAV-NC and *n* = 12 for AAV-*PDK4*). Data are presented as mean ± SD. **F** Two-way ANOVA with Tukey’s correction was used. **H** Two-sided Mann–Whitney *U*-test with the exact method. Source data are provided as a Source Data file.

To further investigate the role of VSMC-PDK4 in AAA, we used recombinant GFP-labeled adeno-associated virus serotype 9 (rAAV9) vectors carrying *PDK4* under the control of the smooth muscle protein 22-α (SM22α) promoter to overexpress PDK4 in an AAV-*PCSK9*^*DY*^/Ang II-induced AAA mouse model. Wild-type C57BL/6J mice were injected with AAV-*PCSK9*^*DY*^ at eight weeks of age, followed by AAV-NC or AAV-*PDK4* injection via the tail vein at ten weeks of age, two weeks before Ang II infusion (Fig. 3A). Immunofluorescence analysis confirmed successful transduction of GFP-labeled AAV9 into aortic tissues in mice injected with either AAV-*PDK4* or AAV-NC (Fig. S4A). Moreover, Western blot and immunofluorescence results demonstrated that PDK4 levels were markedly elevated in both MASMCs and mouse aortic tissues injected with AAV-*PDK4* compared with those injected with AAV-NC (Fig. S4B–D). No differences in aortic morphology or maximal abdominal aortic diameter were observed between saline-infused mice injected with AAV-NC and those injected with AAV-*PDK4* (Fig. 3B, F). However, following Ang II infusion, mice injected with AAV-*PDK4* exhibited a significantly higher mortality rate (42.9% vs. 14.3%), greater AAA incidence (85.7% vs. 52.4%), a larger maximal abdominal aortic diameter, and more severe elastin degradation than mice injected with AAV-NC (Fig. 3B–H). Despite these changes, blood pressure, body weight, plasma lipid levels, and aortic root plaque areas were comparable between the groups (Fig. S4E–I). These results suggest that VSMC-specific PDK4 overexpression exacerbates AAA development.

### VSMC-PDK4 deficiency reduces VSMC phenotypic switch

**Fig. 4:**
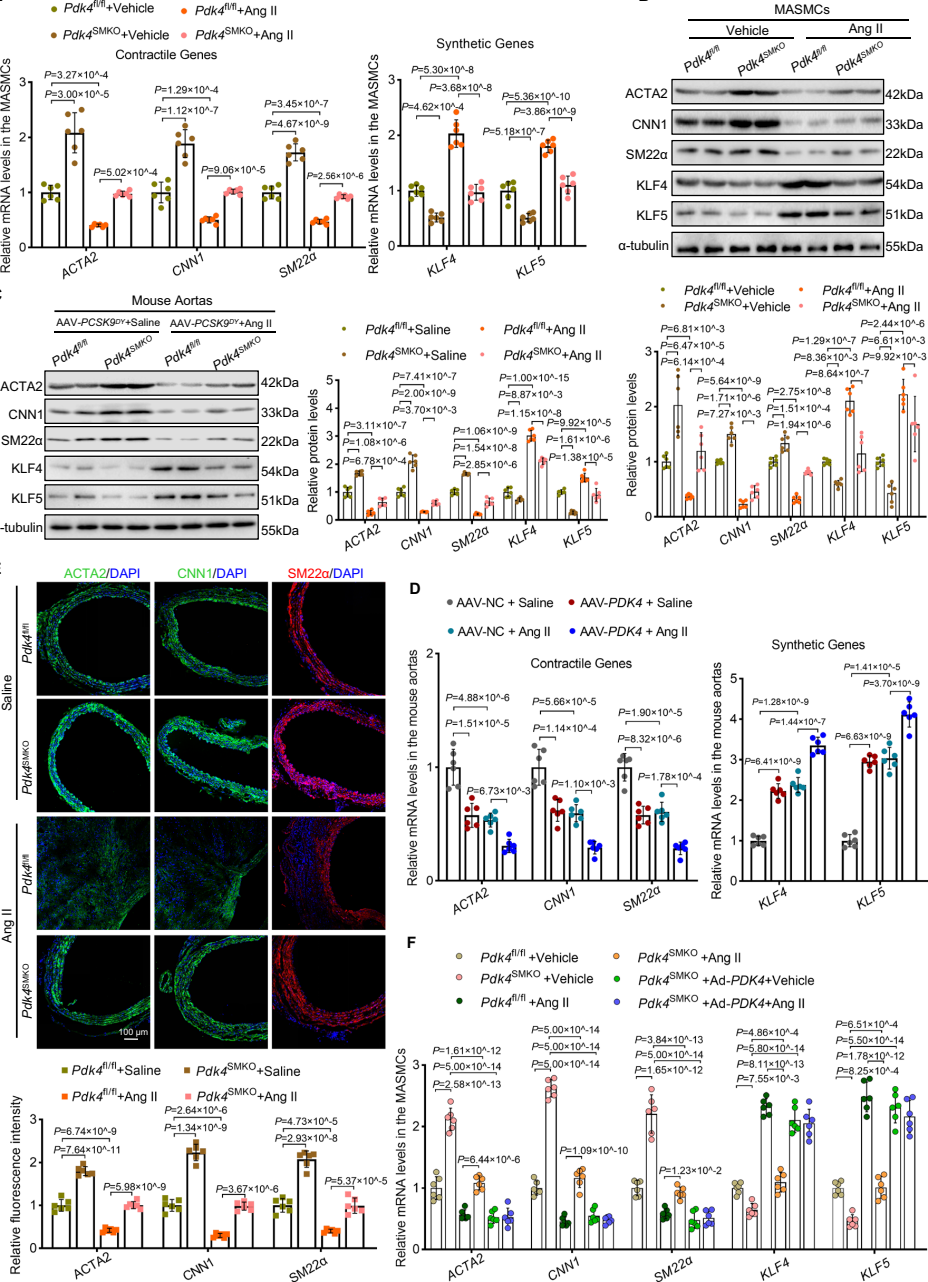
VSMC-PDK4 deficiency inhibits the phenotypic switch of VSMCs from a contractile to a synthetic state. **A** Relative mRNA levels of *ACTA2*, *CNN1*, *SM22α*, *KLF4*, and *KLF5* in mouse aortic smooth muscle cells (MASMCs) from *Pdk4*^SMKO^ and *Pdk4*^fl/fl^ mice treated with vehicle or Ang II (1 μM, 48 h) by RT-qPCR analysis (*n* = 6 biological replicates). **B** Representative Western blot and quantification of ACTA2, CNN1, SM22α, KLF4, and KLF5 protein levels in MASMCs from *Pdk4*^SMKO^ and *Pdk4*^fl/fl^ mice treated with vehicle or Ang II (1 μM, 48 h) (*n* = 6 biological replicates). **C** Representative Western blot and quantification of ACTA2, CNN1, SM22α, KLF4, and KLF5 protein levels in the abdominal aortas of *Pdk4*^SMKO^ and *Pdk4*^fl/fl^ mice treated with AAV-*PCSK9*^*DY*^/Ang II or AAV-*PCSK9*^*DY*^/saline (*n* = 6 biological replicates). **D** Relative mRNA levels of *ACTA2*, *CNN1*, *SM22α*, *KLF4*, and *KLF5* were measured by RT-qPCR analysis in the abdominal aortas from AAV-*PDK4* and AAV-NC mice treated with AAV-*PCSK9*^*DY*^/Ang II for 28 days or AAV-*PCSK9*^*DY*^/saline for 28 days (*n* = 6 biological replicates). **E** ACTA2, CNN1, SM22α immunofluorescence in suprarenal abdominal aortas from *Pdk4*^SMKO^ and *Pdk4*^fl/fl^ mice treated with AAV-*PCSK9*^*DY*^/Ang II for 28 days or AAV-*PCSK9*^*DY*^/saline for 28 days (*n* = 6 biological replicates). **F** Relative mRNA levels of *ACTA2*, *CNN1*, *SM22α*, *KLF4*, and *KLF5* in MASMCs isolated from *Pdk4*^SMKO^ and *Pdk4*^fl/fl^ mice, treated with vehicle or Ang II (1 μM, 48 h), and transfected with Ad-*PDK4* as indicated (*n* = 6 biological replicates). Data are presented as mean ± SD. **A**–**E** two-way ANOVA followed by Tukey’s multiple-comparisons test. **F** One-way ANOVA followed by Tukey’s multiple-comparisons test. Source data are provided as a Source Data file.

Phenotypic switching is crucial for VSMC function^17^. Therefore, we analyzed the effect of PDK4 on VSMC phenotypic switching. MASMCs were isolated from the abdominal aortas of *Pdk4*^fl/fl^ and *Pdk4*^SMKO^ mice, followed by treatment with vehicle or Ang II. RT-qPCR analysis showed that mRNA levels of contractile markers (*ACTA2, CNN1, and SM22α*) were significantly increased, while mRNA levels of synthetic markers (*KLF4, KLF5*) were significantly decreased in vehicle or Ang II-treated MASMCs from *Pdk4*^SMKO^ mice compared to those from *Pdk4*^fl/fl^ mice (Fig. 4A). Western blot revealed a significant increase in the protein levels of contractile markers and a significant decrease in the protein levels of synthetic markers in vehicle or Ang II-treated MASMCs from *Pdk4*^SMKO^ mice compared to those from *Pdk4*^fl/fl^ mice (Fig. 4B). Western blot showed that abdominal aortas from *Pdk4*^SMKO^ mice exhibited increased protein expression of contractile markers and decreased expression of synthetic markers compared to those from *Pdk4*^fl/fl^ mice in the AAV-*PCSK9*^*DY*^/Ang II-induced AAA model (Fig. 4C). Conversely, RT-qPCR analysis indicated that VSMC-PDK4 overexpression decreased the mRNA levels of contractile markers and increased those of synthetic markers in the abdominal aortas from AAV-*PDK4* -treated mice and AAV-NC-treated mice after AAV-*PCSK9*^*DY*^/Ang II treatment (Fig. 4D). Similarly, immunofluorescence staining of mouse suprarenal abdominal aortic tissues confirmed that *Pdk4*^SMKO^ mice showed significantly higher expression of contractile markers than *Pdk4*^fl/fl^ mice under both AAV-*PCSK9*^*DY*^/Ang II or AAV-*PCSK9*^*DY*^/saline conditions (Fig. 4E). Subsequently, MASMCs isolated from *Pdk4*^fl/fl^ or *Pdk4*^SMKO^ mice were transfected with Ad-*PDK4*, followed by treatment with either vehicle or Ang II. RT-qPCR showed that PDK4 deficiency increased the mRNA expression of contractile markers and decreased the expression of synthetic markers, which was reversed by PDK4 overexpression (Fig. 4F). These results suggest that PDK4 deficiency in VSMCs inhibits the phenotypic switch from a contractile to a synthetic state.

### PDK4 alters mitochondrial respiration

**Fig. 5:**
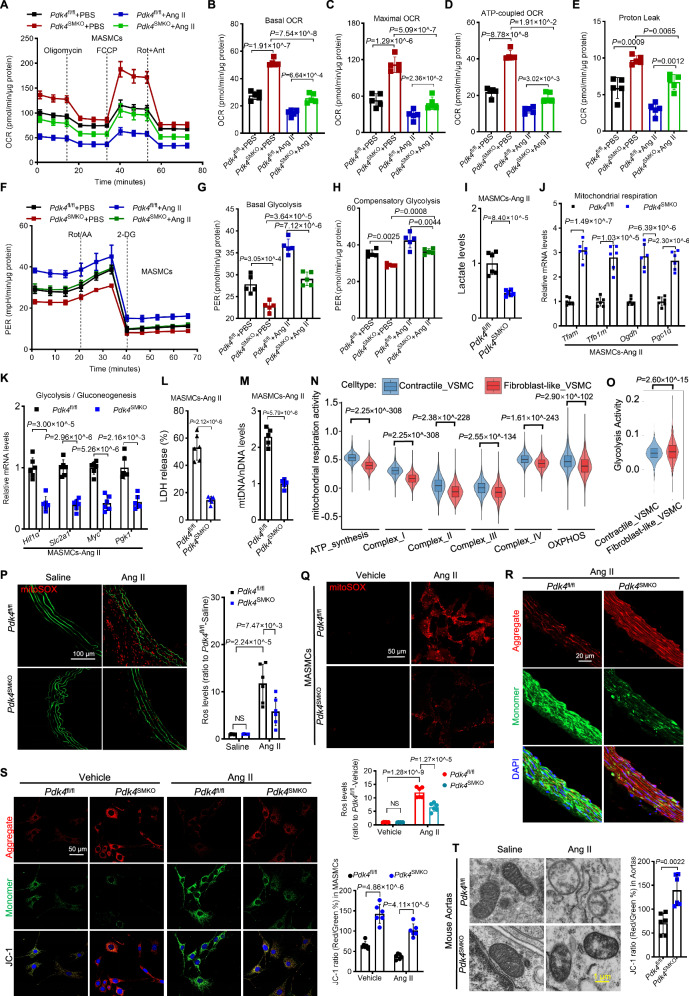
PDK4 alters mitochondrial respiration and increases ROS production. **A**–**H** Oxygen consumption rate (OCR) and proton efflux rate (PER) were detected in MASMCs from *Pdk4*^fl/fl^ and *Pdk4*^SMKO^ mice, treated with PBS or Ang II (1 μM, 48 h). Basal OCR, Maximal OCR, ATP-coupled OCR, Proton leak, Basal and Compensatory glycolysis were measured (*n* = 5 biological replicates). **I**–**M** Measurements in MASMCs from *Pdk4*^fl/fl^ and *Pdk4*^SMKO^ mice after treatment with Ang II (1 μM, 48 h). **I** Lactate levels (*n* = 6 biological replicates). **J**, **K** mRNA levels of mitochondrial respiration and glycolysis genes (*n* = 6 biological replicates). **L** Lactate dehydrogenase (LDH) levels (*n* = 6 biological replicates). **M** mtDNA/nDNA levels (*n* = 5 biological replicates). **N**, **O** Violin plot showing the scores of mitochondrial respiration and glycolysis pathway activity in Contractile_VSMC and Fibroblast-like_VSMC clusters (*n* = 4971 cells for Contractile_VSMC and *n* = 3302 cells for Fibroblast-like_VSMC). **P** Representative mitochondria-derived ROS (MitoSOX) staining of suprarenal abdominal aortas from *Pdk4*^fl/fl^ and *Pdk4*^SMKO^ mice treated with AAV-*PCSK9*^*DY*^/Ang II or AAV-*PCSK9*^*DY*^/saline for 28 days (*n* = 6 biological replicates). **Q** Representative MitoSOX staining in MASMCs from *Pdk4*^fl/fl^ and *Pdk4*^SMKO^ mice, treated with vehicle or Ang II (1 μM, 48 h) (*n* = 6 biological replicates). **R** Representative JC-1 staining in suprarenal abdominal aortas of *Pdk4*^fl/fl^ and *Pdk4*^SMKO^ mice treated with AAV-*PCSK9*^*DY*^/Ang II for 28 days (*n* = 6 biological replicates). **S** Representative JC-1 staining and quantification in MASMCs from *Pdk4*^fl/fl^ and *Pdk4*^SMKO^ mice, treated with vehicle or Ang II (1 μM, 48 h) (*n* = 6 biological replicates). **T** Representative transmission electron microscopy (TEM) images of mitochondria in the abdominal aortas of *Pdk4*^fl/fl^ and *Pdk4*^SMKO^ mice treated with AAV-*PCSK9*^*DY*^/saline or AAV-*PCSK9*^*DY*^/Ang II for 28 days (*n* = 6 biological replicates). Data are presented as mean ± SD. **N**, **O** The center line represents the median. The box bounds represent the interquartile range (IQR), with the upper limit being quartile 3 (Q3) and the lower limit being quartile 1 (Q1). The whiskers extend to Q1 − 1.5 × IQR and Q3 + 1.5 × IQR. **B**–**E**, **G**, **H**, **P**, **Q**, **S** Two-way ANOVA with Tukey’s correction. **I**, **J**, **L**, **M** Two-sided Unpaired Student’s *t*-tests. **K** Two-sided Unpaired Student’s *t-*tests or Mann–Whitney *U*-test (exact method). **N**, **O**, **R** Two-sided Mann–Whitney *U*-test (exact method). NS nonsignificant. Source data are provided as a Source Data file.

The TCA cycle plays a crucial role in maintaining mitochondrial homeostasis. Changes in mitochondrial metabolism can result in impaired mitochondrial respiration, mitochondrial DNA damage, and electron transport chain (ETC) dysregulation^18^. PDK4 is a regulator of the TCA cycle and influences mitochondrial respiration in an expression-dependent manner^19^. Therefore, we investigated the effects of PDK4 deficiency on mitochondrial respiration in VSMCs. Seahorse analysis of mitochondrial oxidative phosphorylation (OXPHOS) showed that VSMC-PDK4 deficiency increased the basal, maximal, and ATP-coupled oxygen consumption rates and proton leak in response to Ang II in MASMCs (Fig. 5A–E). Reprogramming of glucose metabolism from mitochondrial OXPHOS to aerobic glycolysis has been observed during VSMC phenotype switching^20^. MASMCs from *Pdk4*^fl/fl^ and *Pdk4*^SMKO^ mice showed significant increases in basal and compensatory glycolysis in response to Ang II. However, the increase was less pronounced in MASMCs from *Pdk4*^SMKO^ mice (Fig. 5F–H). Lactate levels were significantly decreased in MASMCs isolated from *Pdk4*^SMKO^ mice compared to those from *Pdk4*^fl/fl^ mice (Fig. 5I). Additionally, the mRNA levels of mitochondrial respiration genes, including *Tfam*, *Tfb1m*, *Ogdh*, and *Pgc1α*, were significantly upregulated in MASMCs from *Pdk4*^SMKO^ mice with Ang II treatment, whereas those of glycolysis or gluconeogenesis genes, including *Hif1α*, *Slc2a1*, *Myc*, and *Pgk1*, were markedly decreased compared to MASMCs from *Pdk4*^fl/fl^ mice (Fig. 5J, K). Lactate dehydrogenase (LDH) release and mitochondrial DNA levels were significantly reduced in MASMCs isolated from *Pdk4*^SMKO^ mice compared to those from *Pdk4*^fl/fl^ mice (Fig. 5L, M). VSMC-PDK4 overexpression decreased the mRNA levels of mitochondrial respiration-related genes, increased the mRNA levels of glycolysis-related genes, increased LDH release, and mitochondrial DNA levels in MASMCs after Ang II stimulation (Fig. S5A–D). Subsequent re-analysis of the single-cell RNA-seq data revealed that Fibroblast-like_VSMCs exhibited suppressed mitochondrial respiration pathway activity and elevated glycolysis pathway activity compared to Contractile_VSMCs, as quantified by AUCell scoring^21^ (Fig. 5N, O). Meanwhile, mitochondria-derived ROS fluorescence probe (MitoSOX) staining results showed that mitochondrial ROS production was markedly decreased in suprarenal abdominal aortic tissues from *Pdk4*^SMKO^ mice treated with AAV-*PCSK9*^*DY*^/Ang II and in MASMCs isolated from *Pdk4*^SMKO^ mice after Ang II stimulation compared to those from *Pdk4*^fl/fl^ mice (Fig. 5P, Q). VSMC-PDK4 overexpression increased mitochondrial ROS production in MASMCs after Ang II stimulation (Fig. S5E). In addition, we conducted JC-1 staining on Ang II-treated MASMCs derived from *Pdk4*^fl/fl^ and *Pdk4*^SMKO^ mice, as well as on suprarenal abdominal aortic tissues obtained from AAV-*PCSK9*^*DY*^/Ang II-induced AAA models in *Pdk4*^fl/fl^ and *Pdk4*^SMKO^ mice, to assess mitochondrial membrane potential. The results showed that suprarenal abdominal aortic tissues and MASMCs from *Pdk4*^fl/fl^ mice exhibited significantly lower JC-1 fluorescence intensity compared to *Pdk4*^SMKO^ mice (Fig. 5R, S). However, MASMCs transfected with Ad-*PDK4* exhibited a significantly lower JC-1 fluorescence intensity compared to those transfected with Ad-Con (Fig. S5F). PDK4 regulates pyruvate dehydrogenase (PDH) activity by phosphorylating multiple residues, which inhibits the conversion of glycolytic intermediates into the TCA cycle^22^. Consequently, we assessed PDH activity along with several related metabolites to further explore this regulation. The levels of PDH activity and acetyl-CoA were significantly increased in MASMCs isolated from *Pdk4*^SMKO^ mice compared to those from *Pdk4*^fl/fl^ mice (Fig. S5G,H). However, the level of pyruvate was significantly decreased in MASMCs isolated from *Pdk4*^SMKO^ mice compared to those from *Pdk4*^fl/fl^ mice (Fig. S5I). The Western blot results showed that there was no compensatory upregulation of PDK1, PDK2, or PDK3 levels in vehicle or Ang II-treated MASMCs from *Pdk4*^SMKO^ mice compared to those from *Pdk4*^fl/fl^ mice (Fig. S5J). Transmission electron microscopy (TEM) revealed that VSMC-PDK4 deficiency attenuated mitochondrial swelling and cristae collapse in Ang II-treated MASMCs in the AAV-*PCSK9*^*DY*^/Ang II-induced AAA model (Fig. 5T). These results indicated that PDK4 promotes metabolic reprogramming and alters mitochondrial respiration.

### PDK4 promotes NLRP3-mediated pyroptosis in VSMCs

RNA-seq was performed on MASMCs transfected with Ad-*PDK4* or Ad-Con and subsequently treated with Ang II (1 μM, 48 h) to investigate the mechanisms by which PDK4 regulates VSMC function in AAA pathogenesis (Fig. S6A). Differentially expressed gene (DEG) analysis indicated that PDK4 overexpression significantly reduced mtDNA-encoded gene expression of the ETC complexes in MASMCs (Fig. S6B), consistent with our previous findings. RT-qPCR analysis revealed that the expression of these genes was significantly downregulated in Ang II-treated MASMCs transfected with Ad-*PDK4* compared to those transfected with Ad-Con (Fig. S6C).

**Fig. 6:**
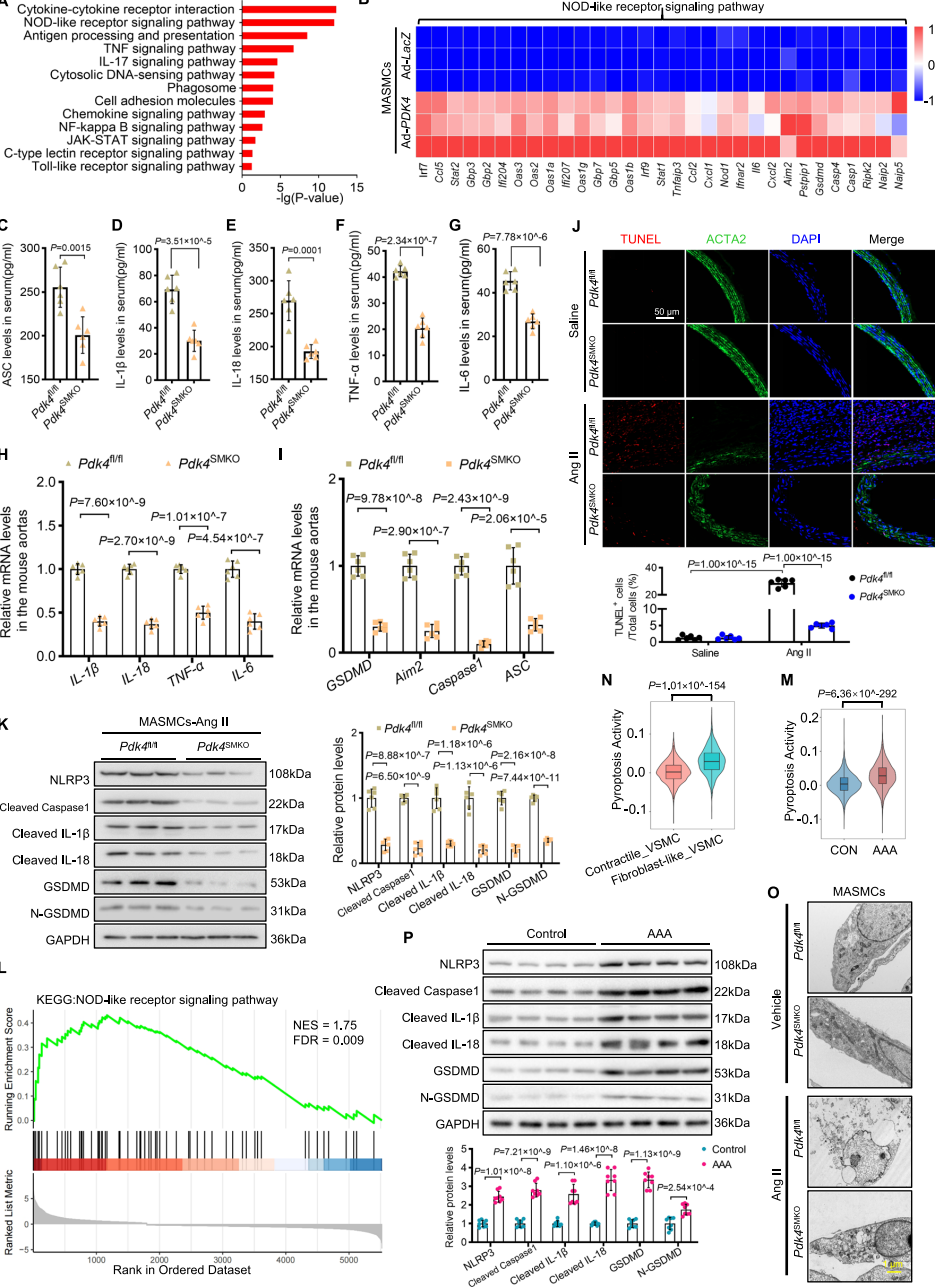
PDK4 induces an inflammatory response and NLRP3-mediated pyroptotic cell death in VSMCs during AAA. **A** KEGG analysis of pathways upregulated in MASMCs transfected with Ad-Con or Ad-*PDK4*, followed by Ang II treatment (1 μM, 48 h). **B** Heat map of RNA-seq from the same cells as in (**A**) (*n* = 3 biological replicates) (blue indicates downregulation; red indicates upregulation). **C**–**G** Serum levels of ASC, IL-1β, IL-18, TNF-α, and IL-6 were measured by ELISA, and **H**, **I** mRNA levels of *IL-1β*, *IL-18*, *TNF-α*, *IL-6*, *GSDMD*, *Aim2*, *Caspase1*, and *ASC* in abdominal aortas were measured in *Pdk4*^SMKO^ and *Pdk4*^fl/fl^ mice treated with AAV-*PCSK9*^*DY*^/Ang II for 28 days (*n* = 6 biological replicates). **J** Representative images of TUNEL staining in suprarenal abdominal aortas of *Pdk4*^fl/fl^ and *Pdk4*^SMKO^ mice treated with AAV-*PCSK9*^*DY*^/saline or AAV-*PCSK9*^*DY*^/Ang II for 28 days (*n* = 6 biological replicates). **K** Representative Western blot and quantification of NLRP3, Cleaved Caspase1, Cleaved IL-1β, Cleaved IL-18, GSDMD, and N-GSDMD protein levels in MASMCs from *Pdk4*^SMKO^ and *Pdk4*^fl/fl^ mice treated with Ang II (1 μM, 48 h) (*n* = 6 biological replicates). **L** GSEA of the KEGG NOD-like receptor signaling pathway in VSMCs comparing control and AAA groups. **M**, **N** Violin plots showing the scores of pyroptosis pathway activity in control and AAA groups (*n* = 5606 cells for control and *n* = 5074 cells for AAA) and Contractile_VSMC and Fibroblast-like_VSMC clusters (*n* = 2466 cells for Contractile_VSMC and *n* = 1634 cells for Fibroblast-like_VSMC). **O** Representative transmission electron microscopy (TEM) images of MASMCs from *Pdk4*^SMKO^ and *Pdk4*^fl/fl^ mice, treated with vehicle or Ang II (1 μM, 48 h) (*n* = 6 biological replicates). **P** Representative Western blot and quantification of NLRP3, Cleaved Caspase1, Cleaved IL-1β, Cleaved IL-18, GSDMD, and N-GSDMD in the abdominal aortas of patients with AAA and adjacent non-aneurysmal segments (control) (*n* = 8 patients). Data are presented as mean ± SD. **M**, **N** The center line represents the median. Box bounds represent the interquartile range (IQR), with the upper limit being quartile 3 (Q3) and the lower limit being quartile 1 (Q1). The whiskers extend to Q1 − 1.5 × IQR and Q3 + 1.5 × IQR. **A** Hypergeometric distribution test. **C**–**I**, **K**, **P** Two-sided Unpaired Student’s *t*-test. **J** Two-way ANOVA with Tukey’s correction. **M**, **N** Two-sided Mann–Whitney *U*-test (exact method). Source data are provided as a Source Data file.

Kyoto Encyclopedia of Genes and Genomes (KEGG) analysis revealed significant enrichment of multiple pathways in Ang II-treated MASMCs transfected with Ad-*PDK4* compared with Ad-Con, highlighting key enriched pathways such as cytokine-cytokine receptor interaction, nucleotide oligomerization domain (NOD)-like receptor signaling, antigen processing and presentation, tumor necrosis factor (TNF) signaling, and interleukin-17 (IL-17) signaling (Fig. 6A). DEG analysis indicated that NOD-like receptor signaling-related genes, such as *Irf7*, *Ccl5*, *Stat2*, *Gsdmd*, and *Caspase1*, were markedly upregulated with PDK4 overexpression (Fig. 6B). Complementarily, RNA-seq was performed on Ang II (1 μM, 48 h)-treated *Pdk4*^SMKO^ and *Pdk4*^fl/fl^ MASMCs (Fig. S6D). KEGG analysis identified significant downregulation of TNF signaling, IL-17 signaling, NOD-like receptor signaling, and cytokine-cytokine receptor interaction pathways (Fig. S6E). DEG analysis confirmed suppression of NOD-like receptor signaling-related genes, such as *Nlrp3*, *Aim2*, *Caspase4*, and *Nod2* in *Pdk4*^SMKO^ MASMCs (Fig. S6F). Previous studies emphasized the crucial role of pyroptosis in AAA^23,24^. These transcriptomic findings suggested that PDK4 may regulate inflammatory signaling, particularly cytokine-cytokine receptor interaction and NOD-like receptor pathways. Pyroptosis is induced by the activation of inflammasome components, including ASC, NOD-like receptors, and caspase1, such as in the most typical NOD-like receptor protein 3 (NLRP3) inflammasome^25^. Serum levels of pyroptosis-related proteins, including ASC, IL-1β, IL-18, TNF-α, and IL-6, were measured to investigate whether PDK4 is related to pyroptosis and the NLRP3 activation pathway. These proteins were significantly decreased in *Pdk4*^SMKO^ mice compared to *Pdk4*^fl/fl^ mice (Fig. 6C–G). RT-qPCR showed that the mRNA levels of *IL-1β*, *IL-18*, *TNF-α*, and *IL-6* were significantly decreased in the abdominal aortas of *Pdk4*^SMKO^ mice compared to *Pdk4*^fl/fl^ mice with AAV-*PCSK9*^*DY*^/Ang II treatment (Fig. 6H). Additionally, RT-qPCR showed that the mRNA levels of pyroptosis-associated molecules, including *gasdermin D (GSDMD)*, *Aim2*, *Caspase1* and *ASC* were significantly decreased in the abdominal aortas of *Pdk4*^SMKO^ mice compared to *Pdk4*^fl/fl^ mice treated with AAV-*PCSK9*^*DY*^/Ang II (Fig. 6I). Terminal deoxynucleotidyl transferase dUTP nick-end labeling (TUNEL) staining revealed fewer TUNEL-positive cells in suprarenal abdominal aortic tissues from *Pdk4*^SMKO^ mice compared to those from *Pdk4*^fl/fl^ mice (Fig. 6J). NLRP3 inflammasome activation promotes caspase1 activation. This process subsequently leads to GSDMD cleavage by activated caspase1. The resulting N-GSDMD fragment is responsible for plasma membrane permeabilization, which ultimately triggers pyroptosis^26^. Western blot analysis revealed that the protein levels of NLRP3, Cleaved Caspase1, Cleaved IL-1β, Cleaved IL-18, GSDMD, and N-GSDMD were significantly decreased in MASMCs from *Pdk4*^SMKO^ mice compared to those from *Pdk4*^fl/fl^ mice with Ang II treatment (Fig. 6K). Gene set enrichment analysis (GSEA) of the reanalyzed single-cell RNA sequencing dataset (GSE239620) further revealed a strong association between NOD-like receptor signaling and AAA progression in VSMCs (Fig. 6L). AAA groups exhibited higher pyroptosis pathway activity than Control groups (Fig. 6M). Fibroblast-like_VSMCs exhibited higher pyroptosis pathway activity than Contractile_VSMCs (Fig. 6N). TEM showed that VSMC-PDK4 deficiency reduced cell swelling and membrane pore formation in MASMCs treated with Ang II (Fig. 6O). Western blot showed that human AAA tissues exhibited higher NLRP3, Cleaved Caspase1, Cleaved IL-1β, Cleaved IL-18, GSDMD, and N-GSDMD expression than control tissues (Fig. 6P). After transfection with siR-*PDK4* and treatment with Ang II (1 μM, 48 h), MASMCs exhibited lower levels of PDK4, NLRP3, Cleaved Caspase1, Cleaved IL-1β, Cleaved IL-18, GSDMD, and N-GSDMD compared to those transfected with siR-Con (Fig. S6G). To determine whether PDK4 also regulates pyroptosis in macrophages, we performed experiments in bone marrow-derived macrophages (BMDMs) transfected with siR-*PDK4* and treated with LPS (100 ng/mL, 4 h)/Nigericin (10 μM, 30 min). Western blot analysis showed no significant differences in the levels of NLRP3, GSDMD, and N-GSDMD between BMDMs transfected with siR-*PDK4* and those transfected with siR-Con (Fig. S6H). After transfection with siR-*PDK1* and treatment with Ang II, MASMCs showed no significant changes in the levels of NLRP3, Cleaved Caspase1, Cleaved IL-1β, Cleaved IL-18, GSDMD, or N-GSDMD compared with siR-Con treated cells (Fig. S6I). These results indicate that VSMC-PDK4 promotes pyroptosis and NLRP3 inflammasome activation in our experimental system. In contrast, PDK4 does not appear to significantly influence pyroptosis in macrophages, and PDK1 knockdown does not attenuate pyroptosis in VSMCs under these conditions.

### ROS-mediated NLRP3 inflammasome activation is involved in PDK4-induced VSMC pyroptosis

**Fig. 7:**
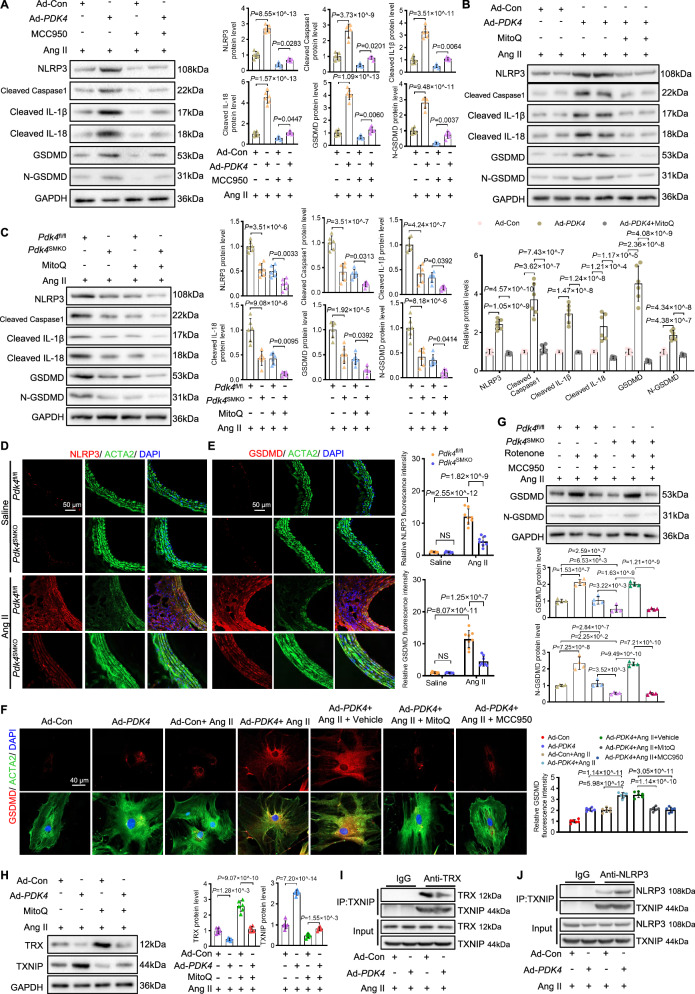
ROS-mediated NLRP3 inflammasome activation is involved in PDK4-induced VSMC pyroptosis. **A** Representative Western blot and quantification of pyroptosis markers in MASMCs transfected with Ad-*PDK4* or Ad-Con for 48 h, pre-treated with MCC950 (10 μM) for 1 h, and stimulated with Ang II (1 μM) for 48 h (*n* = 6 biological replicates). **B** MASMCs were serum-starved and simultaneously transfected with Ad-*PDK4* or Ad-Con for 48 h, followed by treatment with MitoQ (250 nM, 2 h) and then Ang II (1 μM, 48 h). Representative Western blot and quantification of pyroptosis markers (*n* = 6 biological replicates). **C** Representative Western blot and quantification of pyroptosis markers in MASMCs from *Pdk4*^SMKO^ and *Pdk4*^fl/fl^ mice treated with MitoQ (250 nM, 2 h) followed by Ang II (1 μM, 48 h) (*n* = 6 biological replicates). **D**, **E** Immunofluorescence staining of GSDMD, NLRP3, and ACTA2 in suprarenal abdominal aortas of *Pdk4*^SMKO^ and *Pdk4*^fl/fl^ mice treated with AAV-*PCSK9*^*DY*^/Ang II or AAV-*PCSK9*^*DY*^/saline for 28 days (*n* = 8 biological replicates). **F** Immunofluorescence staining of GSDMD and ACTA2 in MASMCs transfected with Ad-Con or Ad-*PDK4* for 48 h, pre-treated with MitoQ (250 nM, 2 h) or MCC950 (10 μM, 1 h), and stimulated with Ang II (1 μM) for 48 h (*n* = 6 biological replicates). **G** Representative Western blot and quantification of GSDMD and N-GSDMD in MASMCs from *Pdk4*^SMKO^ and *Pdk4*^fl/fl^ mice, sequentially treated with vehicle or MCC950 (10 μM, 1 h) and Rotenone (1 μM, 2 h) prior to Ang II (1 μM, 48 h) stimulation (*n* = 4 biological replicates). **H** MASMCs were serum-starved and simultaneously transfected with Ad-*PDK4* or Ad-Con for 48 h, followed by treatment with MitoQ (250 nM, 2 h) and then Ang II (1 μM, 48 h). Representative Western blot and quantification of TRX and TXNIP levels (*n* = 6 biological replicates). **I**, **J** MASMCs were serum-starved and simultaneously transfected with Ad-*PDK4* or Ad-Con for 48 h, followed by treatment with Ang II (1 μM, 48 h). Cell lysates were immunoprecipitated with TXNIP antibody and immunoblotted for TRX or NLRP3 (*n* = 4 biological replicates). Data are presented as mean ± SD. **A**–**C**, **F**–**H** One-way ANOVA followed by Tukey’s multiple-comparisons test. **D**, **E** two-way ANOVA followed by Tukey’s multiple-comparisons test. Source data are provided as a Source Data file.

MASMCs were transfected with Ad-*PDK4* or Ad-Con and treated with vehicle or MCC950, an NLRP3 inflammasome inhibitor, to investigate whether PDK4-mediated pyroptosis is associated with NLRP3 inflammasome activation^27^. MCC950 administration reduced the protein levels of NLRP3, Cleaved Caspase1, Cleaved IL-1β, Cleaved IL-18, GSDMD, and N-GSDMD induced by PDK4 overexpression in MASMCs (Fig. 7A). ROS interact with newly synthesized mtDNA to produce oxidized mtDNA. Oxidized mtDNA is released into the cytosol with mitochondrial damage, activating the NLRP3 inflammasome^28^. MASMCs were transfected with Ad-*PDK4* or Ad-Con in vitro and treated with ROS scavengers to determine whether increased mtROS production is responsible for NLRP3 inflammasome activation induced by PDK4 overexpression^29^. MitoQ is a mitochondria-targeted antioxidant, a derivative of coenzyme Q, that specifically inhibits mitochondrial ROS production^30^. N-acetylcysteine (NAC) acts as a precursor for the synthesis of glutathione and is regarded as a regulator of the intracellular redox balance^31^. Western blot analysis showed that MitoQ and NAC treatment reduced the protein levels of NLRP3, Cleaved Caspase1, Cleaved IL-1β, Cleaved IL-18, GSDMD, and N-GSDMD induced by PDK4 overexpression in MASMCs (Fig. 7B and Fig. S7A). Moreover, MitoQ and NAC treatment further reduced the expression of these proteins in MASMCs, with lower levels observed in *Pdk4*^SMKO^ cells than in *Pdk4*^fl/fl^ cells (Fig. 7C and Fig. S7B). Immunofluorescence analysis demonstrated that suprarenal abdominal aortic tissues from *Pdk4*^SMKO^ mice exhibited decreased GSDMD and NLRP3 expression and increased ACTA2 expression compared to those from *Pdk4*^fl/fl^ mice, whereas VSMC-PDK4 overexpression showed the opposite effect (Fig. 7D, E and Fig. S7C,D). Similarly, immunofluorescence results of MASMCs indicated that MitoQ, NAC, and MCC950 attenuated the PDK4 overexpression-induced increase in GSDMD expression under Ang II treatment (Fig. 7F and Fig. S7E). Rotenone treatment reversed the reduction in the expression of GSDMD and N-GSDMD in MASMCs induced by PDK4 deficiency, and this effect was blocked by MCC950 (Fig. 7G). Moreover, under oxidative stress, ROS promote the dissociation of thioredoxin-interacting protein (TXNIP) from thioredoxin (TRX), allowing TXNIP to activate the NLRP3 inflammasome. MitoQ treatment attenuated PDK4 overexpression-induced TXNIP upregulation and restored TRX protein levels in MASMCs (Fig. 7H). We next evaluated alterations in TXNIP-TRX and TXNIP-NLRP3 interactions using co-immunoprecipitation (Co-IP). The results demonstrated significantly weakened TXNIP-TRX binding in MASMCs transfected with Ad-*PDK4*, whereas TXNIP-NLRP3 interaction was conversely enhanced (Fig. 7I, J). These findings suggest that PDK4 promotes the dissociation of TXNIP from TRX through mitochondrial ROS accumulation, thereby activating the NLRP3 inflammasome. This process contributes to inflammation and subsequent VSMC pyroptosis.

### PDK4 deletion in VSMCs reduces mitochondrial ROS production and suppresses VSMC pyroptosis

**Fig. 8:**
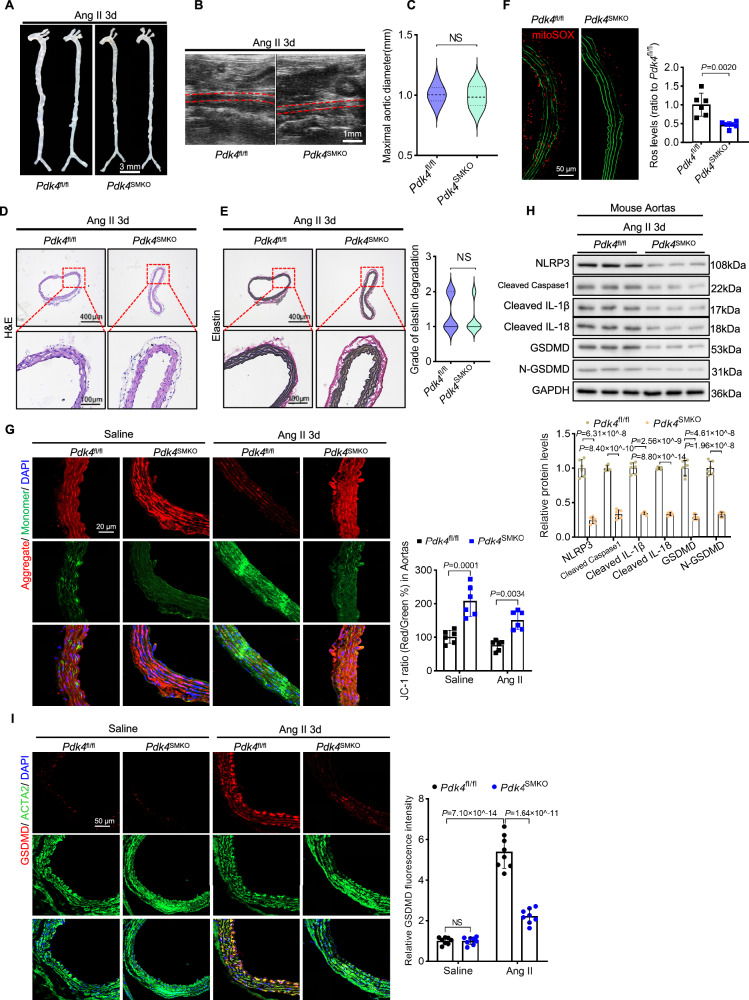
VSMC-PDK4 deficiency reduces mitochondrial ROS production and suppresses VSMC pyroptosis. **A**–**I** Eight-week-old male *Pdk4*^fl/fl^ or *Pdk4*^SMKO^ mice were injected via the tail vein with AAV-*PCSK9*^*DY*^ and fed a Western-type diet. After two weeks, the mice were infused with saline or Ang II (1500 ng/kg/min) for another three days (*n* = 16 for *Pdk4*^fl/fl^ and *Pdk4*^SMKO^). **A** Macrograph of the mouse aorta of the AAV-*PCSK9*^*DY*^/Ang II model established in *Pdk4*^fl/fl^ and *Pdk4*^SMKO^ mice. **B** Representative ultrasound images of the abdominal aorta (scale bar = 1 mm). **C** Maximum diameter of suprarenal abdominal aortas measured by B-mode ultrasound imaging (*n* = 16 for *Pdk4*^fl/fl^; *n* = 16 for *Pdk4*^SMKO^). **D**, **E** Representative hematoxylin and eosin (H&E) and Verhoeff–Van Gieson staining for elastin of the mouse abdominal aorta. The grade of elastin degradation in the aortic wall (*n* = 16 for *Pdk4*^fl/fl^; *n* = 16 for *Pdk4*^SMKO^). **F** Representative MitoSOX staining and quantification in suprarenal abdominal aortas (*n* = 6 biological replicates). **G** Representative JC-1 staining and quantification in suprarenal abdominal aortas (*n* = 6 biological replicates). **H** Representative Western blot and quantification of NLRP3, Cleaved Caspase1, Cleaved IL-1β, Cleaved IL-18, GSDMD, and N-GSDMD protein levels in the abdominal aortas (*n* = 6 biological replicates). **I** Immunofluorescence staining of GSDMD and ACTA2 in suprarenal abdominal aortas was performed (*n* = 8 biological replicates). Data are presented as mean ± SD. **C**, **F**, **H** Two-sided Unpaired Student’s *t*-test. **E** Two-sided Mann–Whitney *U*-test (exact method); **G**, **I** Two-way ANOVA with Tukey’s correction. NS, nonsignificant. Source data are provided as a Source Data file.

To determine whether these findings resulted directly from PDK4 deletion or were a consequence of AAA inhibition, abdominal aortas were collected from *Pdk4*^fl/fl^ and *Pdk4*^SMKO^ mice after 3 days of Ang II treatment, before any detectable changes in AAA formation. No differences were observed in aortic morphology, histological staining, and maximum abdominal aortic diameter between *Pdk4*^SMKO^ and *Pdk4*^fl/fl^ mice infused with AAV-*PCSK9*^*DY*^/Ang II for 3 days (Fig. 8A–E). MitoSOX staining results showed that mitochondrial ROS production was markedly decreased in suprarenal abdominal aortic tissues from *Pdk4*^SMKO^ mice treated with AAV-*PCSK9*^*DY*^/Ang II for 3 days compared with *Pdk4*^fl/fl^ mice (Fig. 8F). The JC-1 staining results showed that suprarenal abdominal aortic tissues from *Pdk4*^fl/fl^ mice exhibited significantly lower JC-1 fluorescence intensity compared with *Pdk4*^SMKO^ mice treated with AAV-*PCSK9*^*DY*^/Ang II or AAV-*PCSK9*^*DY*^/saline for 3 days (Fig. 8G). Western blot analysis revealed that the protein levels of NLRP3, Cleaved Caspase1, Cleaved IL-1β, Cleaved IL-18, GSDMD, and N-GSDMD were significantly decreased in abdominal aortic tissues from *Pdk4*^SMKO^ mice compared to those from *Pdk4*^fl/fl^ mice treated with AAV-*PCSK9*^*DY*^/Ang II for 3 days (Fig. 8H). Immunofluorescence analysis demonstrated that suprarenal abdominal aortic tissues from *Pdk4*^SMKO^ mice exhibited decreased GSDMD expression and increased ACTA2 expression compared to those from *Pdk4*^fl/fl^ mice with AAV-*PCSK9*^*DY*^/Ang II or AAV-*PCSK9*^*DY*^/saline for 3 days treatment (Fig. 8I). These findings suggest that PDK4 deletion restores mitochondrial membrane potential, reduces mitochondrial ROS production, and suppresses VSMC pyroptosis during the early stage of AAA development after 3 days of Ang II treatment.

### DCA alleviates the formation of AAA in mice

**Fig. 9:**
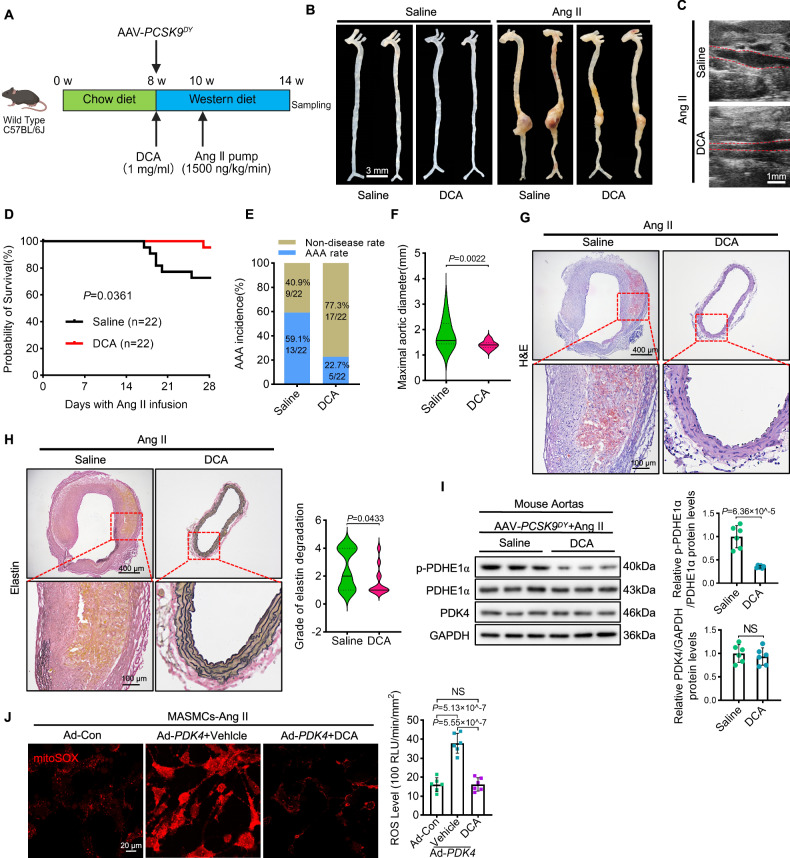
Dichloroacetate (DCA) alleviates AAA formation in mice. **A**–**I** Eight-week-old male C57BL/6J mice were injected with AAV-*PCSK9*^*DY*^, fed a Western diet, and concurrently treated with DCA or saline. After two weeks, they were infused with Ang II (1500 ng/kg/min) for another four weeks (*n* = 22 for DCA and *n* = 22 for saline). **A** Schematic of the AAV-*PCSK9*^*DY*^/Ang II + DCA model established in mice. Created with BioRender. Liu (2026) https://BioRender.com/bpj0bv5. **B** Macrographs of mouse aorta. **C** Representative ultrasound images of the abdominal aorta (scale bar = 1 mm). **D** Survival curves were analyzed using the Kaplan–Meier method and compared using log-rank tests. **E** Abdominal aortic aneurysm (AAA) incidence. **F** Maximum diameter of suprarenal abdominal aortas measured by B-mode ultrasound imaging (*n* = 16 for saline, *n* = 21 for DCA). **G**, **H** Representative hematoxylin and eosin (H&E) and Verhoeff–Van Gieson staining for elastin of the mouse suprarenal abdominal aorta. Grade of elastin degradation in the aortic wall (*n* = 16 for saline; *n* = 21 for DCA). **I** Representative Western blot and quantification of p-PDHE1α, PDHE1α, and PDK4 protein levels in the abdominal aortas (*n* = 6 biological replicates). **J** Representative MitoSOX staining in MASMC which were transfected with Ad-Con or Ad-*PDK4*, then pre-treated with either vehicle or DCA (5 mM) for 48 h, and subsequently stimulated with Ang II (1 μM) for an additional 48 h (*n* = 6 biological replicates). Data are presented as mean ± SD. **F**, **I** Two-sided Unpaired Student’s *t*-test. **H** Two-sided Mann–Whitney *U*-test (exact method). **J** One-way ANOVA followed by Tukey’s multiple-comparisons test. NS nonsignificant. Source data are provided as a Source Data file.

To investigate the therapeutic potential of PDK4 inhibition for AAA, we employed dichloroacetate (DCA), a pharmacological inhibitor of PDK4 activity. Wild-type C57BL/6J mice were injected with AAV-*PCSK9*^*DY*^ at eight weeks of age and treated with DCA. Two weeks after AAV injection, Ang II was infused for the final four weeks (Fig. 9A). The aortic morphology of mice treated with saline or DCA remained unchanged under saline conditions. However, DCA inhibited AAV-*PCSK9*^*DY*^/Ang II-induced AAA progression in mice (Fig. 9B). Mice treated with DCA showed a decreased mortality rate (4.5%), AAA incidence (22.7%), and maximum abdominal aortic diameter compared to saline-treated mice (27.2% mortality rate and 59.1% AAA incidence) (Fig. 9C–F). DCA reduced elastin degradation (Fig. 9G, H). Western blot analysis of abdominal aortic tissues from AAV-*PCSK9*^DY^/Ang II-treated mice revealed significantly decreased protein levels of p-PDHE1α, without altering the expression of PDHE1α or PDK4, in the DCA group compared to the saline group, demonstrating effective inhibition of PDK4 activity (Fig. 9I). Moreover, DCA attenuated mitochondrial ROS production induced by PDK4 overexpression in Ang II-stimulated MASMCs (Fig. 9J). Collectively, these results suggest that DCA mitigates AAA progression through inhibition of PDK4 activity.

### MCC950 supplementation reverses the effect of PDK4 in AAA

**Fig. 10:**
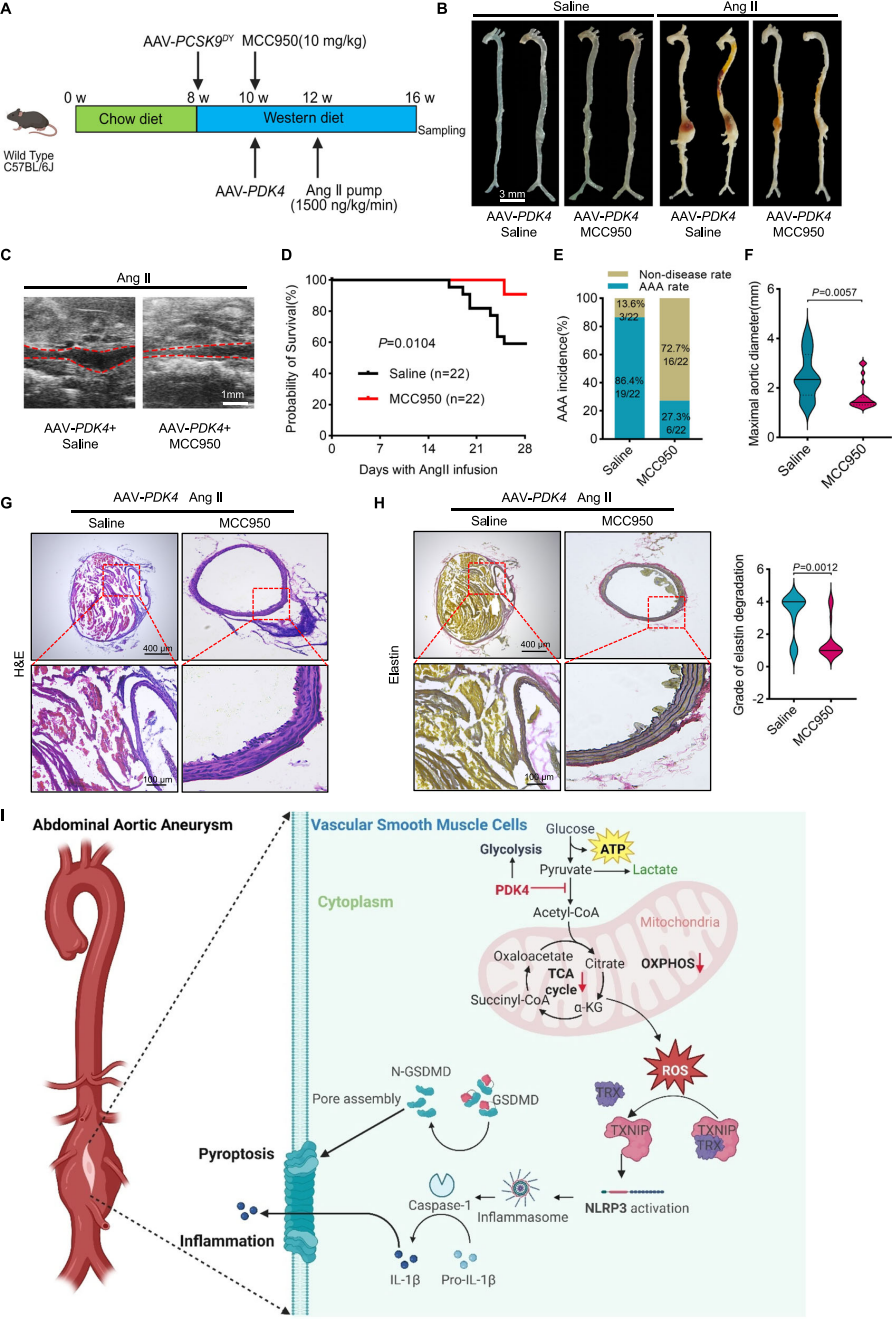
MCC950 alleviates AAA formation in mice with VSMC-PDK4 overexpression. **A**–**H** Eight-week-old male C57BL/6J mice were injected with AAV-*PCSK9*^*DY*^, fed a Western diet, and subsequently treated with AAV-*PDK4* and MCC950 or saline. After two weeks, they were infused with Ang II (1500 ng/kg/min) for another four weeks (*n* = 22 for MCC950 and *n* = 22 for saline). **A** Schematic representation of the AAV-*PCSK9*^*DY*^/Ang II + MCC950 model in AAV-*PDK4* mice. Created with BioRender. Liu (2026) https://BioRender.com/npaw44a. **B** Macrographs of mouse aorta. **C** Representative ultrasound images of the abdominal aorta (scale bar = 1 mm). **D** Survival curves were analyzed using the Kaplan–Meier method and compared using log-rank tests. **E** Abdominal aortic aneurysm (AAA) incidence. **F** Maximum diameter of suprarenal abdominal aortas measured by B-mode ultrasound imaging (*n* = 13 for saline, *n* = 20 for MCC950). **G**, **H** Representative hematoxylin and eosin (H&E) and Verhoeff–Van Gieson staining for elastin of the mouse suprarenal abdominal aorta. Grade of elastin degradation in the aortic wall (*n* = 13 for saline; *n* = 20 for MCC950). **I** Schematic illustration of the functional role of VSMC-PDK4 in AAA formation. Created with BioRender. Liu (2026) https://BioRender.com/jvwc1lz. Data are presented as mean ± SD. **F**, **H** Two-sided Mann–Whitney *U*-test (exact method). Source data are provided as a Source Data file.

Building on our results on the role of PDK4 in AAA development, we investigated whether the inhibition of NLRP3 inflammasome activation can attenuate AAA progression in AAV-*PDK4* mice. We evaluated the effects of MCC950 on AAA development and progression in an AAA mouse model. AAV-*PDK4* was used to overexpress PDK4 in an AAV-*PCSK9*^*DY*^/Ang II-induced AAA mouse model. Wild-type C57BL/6J mice were injected with AAV-*PCSK9*^*DY*^ at eight weeks of age, followed by AAV-*PDK4* injection via the tail vein at ten weeks, with MCC950 treatment administered two weeks prior to Ang II infusion (Fig. 10A). The aortic morphology of AAV-*PDK4* mice treated with saline or MCC950 remained unchanged under saline conditions. However, MCC950 inhibited AAV-*PCSK9*^*DY*^/Ang II-induced AAA progression in AAV-*PDK4* mice (Fig. 10B). Mice injected with AAV-*PDK4* and treated with MCC950 showed a decreased mortality rate (9.1%), AAA incidence (27.3%), and maximum abdominal aortic diameter compared to saline-treated mice (40.9% mortality rate and 86.4% AAA incidence) (Fig. 10C–F). MCC950 reduced elastin degradation without affecting body weight, blood pressure, or plasma lipid levels (Fig. 10G, H and Fig. S8A–D). Moreover, MCC950 treatment significantly decreased serum levels of ASC, IL-1β, IL-18, TNF-α, and IL-6 in AAV-*PDK4*-injected mice (Fig. S9A–E). The pyroptosis-associated protein levels of NLRP3, cleaved caspase1, cleaved IL-1β, cleaved IL-18, GSDMD, and N-GSDMD were significantly reduced with MCC950 treatment compared to saline treatment (Fig. S9F). TUNEL staining indicated reduced TUNEL^+^ cells in suprarenal abdominal aortic tissues of AAV-*PDK4* mice treated with MCC950 compared to saline treatment (Fig. S9G). Immunofluorescence results demonstrated a significant decrease in GSDMD and NLRP3 expression with MCC950 treatment compared with saline treatment (Fig. S9H,I). In addition, Western blot results showed that MCC950 treatment had no significant effect on the expression of PDK4 in MASMCs (Fig. S9J). However, MCC950 treatment significantly increased the expression of contractile markers and decreased the expression of synthetic markers compared to the saline treatment (Fig. S9K). These findings suggested that MCC950 exerts a protective effect against AAA progression via a PDK4-dependent mechanism in VSMCs. Overall, these data demonstrated that PDK4 activates the NLRP3 inflammasome in VSMCs, thereby promoting pyroptosis and AAA progression.

## Discussion

AAA is a prevalent cardiovascular condition associated with substantial morbidity and mortality in older adults because of its potential for rupture^1^. AAA is associated with atherosclerosis, neovascularization, transmural degeneration, VSMC apoptosis, and chronic inflammation^32^. Key risk factors for AAA include smoking, genetic predisposition, male sex, and advancing age^33^, with epidemiological studies showing a 4-6 times greater prevalence in males compared to females^34^. In experimental models, male C57BL/6 mice exhibit greater susceptibility to AAA induction^35^. Currently, treatment options for AAA are limited to open or endovascular surgical repair, and no effective drugs exist to prevent its onset and progression^36^. In this study, we found that VSMC-PDK4 plays a significant role in AAA progression, as demonstrated by murine models of VSMC-PDK4 deficiency or overexpression. We found that PDK4 expression was elevated in the abdominal aortic tissues of patients with AAA and AAA mouse models, whereas VSMC-specific PDK4 knockout inhibited AAA development in mice. PDK4 deficiency in VSMCs prevents the contractile-to-synthetic phenotypic switch. Mechanistically, PDK4 altered mitochondrial respiration and accelerated metabolic reprogramming, resulting in mitochondrial ROS production, NLRP3 inflammasome activation, and proinflammatory cytokine release. This promotes pyroptosis in VSMCs, ultimately resulting in AAA development and progression. Furthermore, MCC950, an inhibitor of the NLRP3 inflammasome, reduced pyroptosis-associated protein expression and limited AAA progression in a PDK4-dependent manner. Our findings suggest that targeting VSMC-PDK4 is a potential therapeutic strategy against AAA. In addition, Oil Red O staining revealed comparable plaque burden between genotypes, suggesting that the differences in AAA severity are unlikely to result from changes in atherosclerotic lesion formation.

We employed AAV-*PCSK9*^*DY*^/Ang II-induced models to investigate the effect of PDK4 on AAA. An AAV encoding *PCSK9* D377Y (Asp374-to-Tyr mutant) targets the low-density lipoprotein (LDL) receptor of hepatocytes and induces its degradation, resulting in hyperlipidemia^37^. This model, combined with Ang II infusion and a Western-type diet, was used to induce AAA^38^. VSMC-specific knockout of PDK4 inhibited AAA development, as evidenced by a decreased incidence rate, reduced maximum aortic diameter, and lower elastin degradation. In contrast, PDK4 overexpression accelerated AAA progression, thereby reinforcing the promotive role of PDK4 in AAA formation.

PDK4 is a crucial enzyme located in the mitochondrial matrix and plays a significant role in cellular metabolism by regulating PDC activity^12^. PDK4 phosphorylates and inactivates PDC, thereby suppressing glucose oxidation and promoting a metabolic shift toward glycolysis and fatty acid oxidation. This shift can profoundly affect cellular energy production and metabolic homeostasis^39^. Recent studies on PDK4 have primarily focused on its role in cancer. However, its impact and underlying mechanisms in cardiovascular diseases, particularly AAA, are not well understood^40^. Our results demonstrate that PDK4 promotes metabolic reprogramming in VSMCs.

Previous studies have provided important insights into the role of pyruvate dehydrogenase kinase (PDK) isoforms in regulating inflammation and cellular metabolism in different contexts. For example, dichloroacetate (DCA), a broad inhibitor of PDK isoforms, has been shown to reduce atherosclerosis and IL-1β release in macrophages^11^. Although this study highlighted the anti-inflammatory effects of DCA in macrophages, it did not examine its effects in VSMCs or directly address the role of PDK4 in NLRP3 inflammasome activation. Another study showed that DCA suppresses NLRP3-mediated pyroptosis in BV2 microglial cells by reducing ROS production and stabilizing mitochondrial membrane potential^41^. Although PDK4 was mentioned in that context, the study focused on microglia and did not mechanistically investigate the direct involvement of PDK4 in NLRP3 activation. In addition, elevated PDK4 expression has been associated with inflammasome activation and apoptosis in skeletal muscle in a polycystic ovary syndrome (PCOS) rat model^42^. However, that study neither established a direct causal relationship between PDK4 and NLRP3 activation nor explored the relevance of PDK4 in vascular pathology. Thus, previous findings were limited to specific cell types, including macrophages, microglia, and skeletal muscle cells, and did not provide mechanistic evidence linking PDK4 to NLRP3 inflammasome activation in VSMCs. In contrast to these earlier studies, our work provides direct evidence that PDK4 induces metabolic changes in VSMCs characterized by altered mitochondrial respiration, increased mitochondrial ROS production, and NLRP3 inflammasome activation. NLRP3 inflammasome activation triggers pyroptosis, a type of inflammatory cell death that exacerbates vascular inflammation and AAA progression. This mechanistic link between PDK4 and NLRP3 activation in VSMCs has not been previously established and provides new insights into the pathogenesis of AAA.

A Recent study demonstrated that DCA attenuated neutrophil-mediated inflammation in the pancreatic elastase (PPE)-induced AAA model via a PDK1-related mechanism, but without in vivo evidence specifically in VSMCs^43^. Building upon this earlier finding, our study provides complementary insights by identifying PDK4 as an important regulator through several lines of evidence. First, single-cell RNA sequencing analysis of mouse AAA tissues identified PDK4 as the most differentially expressed PDK isoform in VSMCs in AAA. This isoform specificity was further validated using VSMC-specific PDK4 knockout and overexpression models in our AAV-*PCSK9*^*DY*^/Ang II-induced AAA system, which recapitulates key clinical features, including hyperlipidemia and hypertension. Moreover, our results demonstrate that DCA mitigates AAV-*PCSK9*^*DY*^/Ang II-induced AAA progression. Importantly, we delineated a novel PDK4/ROS/NLRP3/pyroptosis axis wherein PDK4-mediated metabolic reprogramming in VSMCs promotes mitochondrial dysfunction, NLRP3 inflammasome activation, and subsequent pyroptosis.

The specific involvement of PDK4 observed in our study differs from the PDK1 regulation reported in the PPE-induced AAA model^43^. This divergence is likely influenced by the intrinsic pathophysiological differences between the two models. The PPE model predominantly induces acute inflammatory injury and elastin degradation, whereas the AAV-*PCSK9*^*DY*^/Ang II model, combined with a Western diet, incorporates additional metabolic and hemodynamic components, including hyperlipidemia and hypertension^44^. Such model-dependent differences in inflammatory context, vascular stress, and metabolic status may engage distinct upstream signaling pathways and thereby preferentially activate different PDK isoforms. Notably, a recent study demonstrated that VSMC-*PDK1* deficiency confers protection against aortic aneurysm and dissection (AAD) in the high-fat diet plus Ang II model^45^, directly implicating PDK1 in VSMC-driven pathogenesis. Considered together with our findings, this new evidence suggests that the PDK/PDH axis, rather than any single PDK isoform, functions as a central metabolic node in AAA development. Both PDK1 and PDK4 can be functionally relevant, likely operating in a context-dependent manner by influencing different cell types or responding to distinct pathological stimuli. This conceptual framing accommodates the possibility that multiple PDKs converge on common downstream metabolic consequences, with the specific isoform engaged being determined by the cellular or pathological context. Future comparative studies across diverse AAA models will be valuable to delineate isoform-specific and cell-type-specific roles, and to assess the translational potential of targeting the PDK/PDH axis as a unified therapeutic strategy.

TXNIP functions as a physiological inhibitor of reduced TRX by directly binding to its redox-active catalytic domain, thereby suppressing TRX reductase activity. Under oxidative stress, ROS accumulation triggers TXNIP dissociation from the TXNIP-TRX complex, enabling its translocation to bind NLRP3 and initiate inflammasome assembly^46^. We propose that PDK4 alters mitochondrial respiration, leading to disruption of mitochondrial membrane potential and excessive production of mitochondrial ROS. These ROS serve as upstream activators of the NLRP3 inflammasome by triggering TXNIP dissociation from the TXNIP-TRX complex. This concept is supported by previous studies on the ROS-TXNIP-NLRP3 axis in other diseases^46–48^.

VSMCs play a crucial role in maintaining vascular tone and structure and exhibit two main phenotypic states: contractile and synthetic^49^. The contractile phenotype is characterized by the expression of contractile proteins, such as ACTA2, myosin heavy chain 11 (MYH11), and SM22α, which enable these cells to regulate blood vessel diameter and blood pressure through contraction and relaxation. These VSMCs have a spindle-like shape and low proliferative and migratory capacity^50–52^. Conversely, the synthetic phenotype is characterized by reduced expression of contractile proteins and increased production of extracellular matrix components, cytokines, and growth factors, along with elevated expression of KLF4 and KLF5^53,54^. Synthetic VSMCs can proliferate and migrate, contributing to tissue remodeling and repair^55^. These cells typically have a more rhomboid shape and are actively involved in pathological conditions, such as aortic aneurysm and dissection, where they switch from a contractile to a synthetic phenotype in response to vascular injury or stress^56^. The phenotypic plasticity of VSMCs is essential for their ability to adapt to physiological and pathological stimuli. However, this plasticity plays a pivotal role in the progression of various vascular diseases. Understanding the mechanisms underlying this phenotypic switch is crucial for developing targeted therapies for cardiovascular diseases^57,58^. In the present study, we explored the role of PDK4 in the phenotypic transition of VSMCs. PDK4 overexpression in VSMCs suppressed the expression of contractile proteins, such as CNN1, SM22α, and α-SMA. Conversely, VSMC-specific PDK4 deletion resulted in increased levels of these proteins in the aortas of AAV-*PCSK9*^*DY*^/Ang II-induced AAA mice. These findings indicate that PDK4 promotes the phenotypic switch of VSMCs from a contractile to a synthetic state.

Pyroptosis is a highly inflammatory form of programmed cell death distinct from apoptosis and necrosis^59^. It is characterized by cell swelling, lysis, and the release of proinflammatory cytokines, such as IL-1 and IL-18^60^. Pyroptosis is primarily mediated by the activation of inflammasomes, particularly the NLRP3 inflammasome, which leads to GSDMD cleavage and activation by caspase1. Activated GSDMD forms pores in the cell membrane, resulting in cell lysis and the release of intracellular contents that drive inflammation^61^. ASC is a key adapter protein in the formation of inflammasomes, including NLRP3, which facilitates the recruitment and activation of caspase1^62^. In the context of cardiovascular diseases such as AAA, pyroptosis contributes to the inflammatory milieu that exacerbates disease progression^63^. Several studies have documented that elevated ROS levels can cause oxidative stress, which in turn can result in NLRP3 inflammasome activation^64–66^. Previous studies have shown that PDK4 promotes macrophage polarization toward the M1 proinflammatory phenotype^67^. Notably, we observed no significant effect on pyroptosis when PDK4 was inhibited in macrophages, suggesting that PDK4 does not directly regulate pyroptosis in macrophages. Meanwhile, our study highlighted the role of PDK4 in promoting pyroptosis in VSMCs. We found that PDK4 upregulation in VSMCs activated the mitochondrial ROS-mediated NLRP3 inflammasome, resulting in increased pyroptosis and inflammation. This mechanistic insight suggests that targeting PDK4 and associated pyroptotic pathways may be a potential therapeutic strategy for mitigating AAA progression.

MCC950, a potent and selective inhibitor of the NLRP3 inflammasome, effectively attenuates PDK4-mediated NLRP3 activation and pyroptosis^68^. The inhibition of the NLRP3 inflammasome using MCC950 has been shown to have protective effects in models of aortic aneurysms and dissections. In a previous study^69^, MCC950 treatment effectively reduced the development and progression of aortic aneurysms and dissections in mice by suppressing NLRP3-caspase1 inflammasome activation and MMP-9 activation. In our study, we demonstrated that PDK4 plays a crucial role in activating the NLRP3 inflammasome, thereby promoting the development of AAA in AAV-*PCSK9*^*DY*^/Ang II-induced AAA models. This finding adds a novel layer to the understanding of the molecular mechanisms underlying AAA, as PDK4 activation leads to the induction of NLRP3 inflammasome activation, which contributes to inflammation and VSMC pyroptosis. By targeting PDK4, we can modulate NLRP3 inflammasome activation, offering a potential therapeutic strategy for AAA.

Despite the mechanistic framework established in this study, the full sequence of events linking PDK4-mediated mitochondrial reprogramming to pyroptosis during AAA progression remains incompletely defined. Our findings support a model in which PDK4 disrupts mitochondrial respiration and increases mitochondrial ROS, thereby activating the TXNIP-NLRP3 inflammasome axis in VSMCs. However, whether PDK4-driven mitochondrial dysfunction promotes AAA primarily through NLRP3-dependent pyroptosis or also engages additional ROS-responsive or inflammasome-independent pathways requires further investigation. Future studies using more refined mechanistic approaches will be necessary to delineate the relative contributions of these pathways to PDK4-driven AAA development. Additionally, although our study observed increased PDK4 expression in VSMCs within AAA tissue and found that LY294002 treatment significantly enhanced PDK4 expression, the upstream regulatory mechanisms driving this upregulation remain unknown. One possible explanation is that genetic or epigenetic mechanisms may contribute to this increased expression, but this requires further exploration.

In summary, our study clarified the role of VSMC-PDK4 in AAA development and progression. Elevated PDK4 expression disrupts VSMC mitochondrial respiration, increases mitochondrial ROS production, and induces NLRP3-mediated pyroptosis. PDK4 hinders the conversion of pyruvate to acetyl-CoA, decreasing TCA cycle activity and mitochondrial oxidative phosphorylation, thereby increasing ROS production. Increased ROS levels activate the NLRP3 inflammasome via TXNIP, resulting in caspase1 activation and pyroptosis. This results in the release of proinflammatory cytokines, such as IL-1, intensifying vascular wall inflammation, which is crucial for AAA progression. Therefore, PDK4 and its downstream signaling pathways may represent promising therapeutic targets for AAA, and pharmacological intervention with DCA or MCC950 may offer potential treatment strategies.

## Methods

### Human samples

Human aortic samples were collected from Nanfang Hospital, Southern Medical University, using protocols approved by the hospital’s institutional review board. Human AAA samples were obtained from patients who were diagnosed with AAA according to the 2022 ACC/AHA Guidelines for the Diagnosis and Management of Aortic Diseases, had an abdominal aortic diameter ≥55 mm, and underwent aortic repair. Control aortic samples were collected from adjacent non-aneurysmal segments of the same patient. All human samples were used with the approval of the Ethical Committee of Nanfang Hospital (approval number: NFEC-2023-476), and written informed consent was obtained from all participants. Participants were not compensated for the use of discarded tissue samples or clinical data. The characteristics of the patients included in this study are listed in Supplementary Table 2. Participant sex was determined based on self-report.

Aortic samples were shipped to our laboratory on wet ice and processed within 2 h of collection. Periaortic fat and intraluminal thrombi were removed, and the samples were rinsed with 0.9% normal saline. The aortic tissues were then divided into several segments and either fixed in 10% formalin and embedded in paraffin for histological analysis, embedded in optimal cutting temperature (OCT) compound for immunofluorescence staining, soaked in RNAlater for RNA analysis, or snap-frozen in liquid nitrogen for protein extraction.

All experiments using human samples were performed according to the principles of the Declaration of Helsinki.

### Animal studies

All animal protocols were approved by the Animal Care and Use Committee of Nanfang Hospital, Southern Medical University (approval number NFYY-2021-1260) and followed the Guide for the Care and Use of Laboratory Animals of the National Institute of Health in China.

To generate PDK4 conditional knockout mice, we purchased *PDK4*-floxed (*Pdk4*^fl/fl^) mice from Cyagen Biosciences (Suzhou, China), which contain loxP sites flanking exons 4, 5, and 6 of *PDK4* (Fig. S3A). Two gRNAs (gRNA1: 5′-CTAGATAGAAATTGTCACAT-3′; gRNA2: 5′-CCTTCTCACAATGTTATCCA-3′) were synthesized and inserted into a gRNA expression vector. This vector was then combined with Cas9 mRNA and single-stranded donor DNAs and microinjected into zygotes from C57BL/6J mice. PCR was used to confirm the successful introduction of the two loxP inserts into the target sites in the founder mice. These founders were subsequently bred with heterozygous *Pdk4*^fl+/^^−^ mice to produce homozygous *Pdk*4^fl/fl^ mice. *Myh11*-Cre mice [B6.FVB-Tg (Myh11-icre/ERT2)1Soff/J; stock no. 019079] were purchased from Jackson Laboratory (Bar Harbor, ME, USA). *Myh11*-Cre/*Pdk4*^fl/fl^ (*Pdk4*^SMKO^) mice were generated by crossing *Pdk4*^fl/fl^ mice with *Myh11*-Cre mice. The *Myh11*-Cre transgene is Y-linked and is therefore transmitted only to male offspring. As a result, the VSMC-specific *Pdk4* knockout model can only be generated in male mice. Accordingly, all animal experiments in this study were performed using male mice. To induce the Cre expression, *Myh11*-Cre/Pdk4^fl/fl^ mice and *Pdk4*^fl/fl^ control mice received intraperitoneal tamoxifen (T5648, Sigma-Aldrich) at 75 mg/kg/day for 5 consecutive days. AAA models were induced 2 weeks after tamoxifen administration. Genotyping was performed by PCR using the primers listed in Supplementary Table 3.

To generate PDK4 conditional overexpression mice, two weeks after AAV-*PCSK9*^*DY*^ injection, ten-week-old male C57BL/6J mice were injected with AAV-NC or AAV-*PDK4* (2 × 10^11^ genomic copies per mouse).

For tamoxifen induction, tamoxifen (20 mg/mL; T5648, Sigma, St. Louis, MO, USA) was prepared in corn oil (C8267, Sigma) and administered intraperitoneally to 6-week-old *Pdk4*^fl/fl^ and *Pdk4*^SMKO^ mice at 75 mg/kg/day for 5 consecutive days. Two weeks later, the AAA model was established.

Mouse *PCSK9*^*DY*^/Ang II model: male mice, eight weeks old, were administered AAV-*PCSK9*^*DY*^ (2 × 10^11^ genomic copies) and maintained on a Western-type diet for either 6 or 8 weeks. Starting two or four weeks after AAV injection, Ang II (1500 ng/kg/min) (Med Chem Express, HY-13948) or saline was infused for the final 4 weeks of the study using a mini-pump (Alzet, model 2004) (Med Chem Express, HY-13948).

In the dichloroacetate (DCA) treatment experiment, after AAV injection, saline or 1 mg/ml DCA (~220 mg/kg/day; 347795, Sigma-Aldrich, St Louis, USA) was administered via drinking water for six weeks in C57BL/6 mice. Treatments were administered via light-protected standard bottles changed weekly.

In the AAA mouse model, abdominal aortic diameters were measured using DP2-BSW software (Olympus Life Science Solutions, Center Valley, PA) in a double-blinded manner. An aneurysm was characterized by an aortic dilation exceeding 50% of the normal size. Mice that died from aortic rupture were included in the analysis of AAA incidence but excluded from subsequent analyses of aortic diameter, serum parameters, and elastin degradation. For pharmacologic treatment experiments, mice of each genotype were randomized into control or treatment groups, ensuring that littermates were allocated to different groups whenever possible. Detailed information on the animal experiments is provided in Supplementary Table 4.

All mice were maintained under a 12-h light/dark cycle at 23 °C and 50–70% relative humidity. Eight-week-old male mice were fed a chow diet or a Western diet (40% kcal fat, 10.5% kcal sucrose, 1.25% cholesterol; XT108C; Jiangsu Xietong Pharmaceutical Bio-engineering, Nanjing, China) for 6 or 8 weeks. Mice were deeply anesthetized with isoflurane, and terminal blood was collected by cardiac puncture. Following blood collection and tissue harvest, death was confirmed by cervical dislocation. All procedures were performed by trained personnel in accordance with institutional animal care guidelines.

### scRNA-seq dataset analysis

Single-cell RNA sequencing data from aortic tissues of control and AAA mice were obtained from the GEO database (GSE239620). The control group included NA (normal aorta) and AAD (abdominal dilated aorta without dissection). The AAA group included AAA (single aortic aneurysm without dissection), IMH-I (aortic dissection/intramural hematoma without aneurysm), IMH-II (single aortic aneurysm with dissection/intramural hematoma), and IMH-III (multiple distinct aortic aneurysms with dissection/intramural hematoma). The scRNA-seq data were analyzed using Seurat (version 5.0.1) for quality control, dimensionality reduction, and clustering. Genes expressed in more than 5 cells and cells with more than 300 detected genes were retained in the expression matrix. Cells with more than 5000 detected genes, fewer than 200 detected genes, or more than 20% mitochondrial reads were excluded. The NormalizeData function was used for normalization, FindVariableFeatures was used to identify highly variable genes, and ScaleData was applied for data scaling. The Harmony package was used to correct batch effects. The top 20 Harmony dimensions were used for t-distributed stochastic neighbor embedding (*t*-SNE) analysis, with a clustering resolution of 1. Cell cluster identities were assigned based on established marker gene expression patterns. Gene expression patterns were visualized using heatmaps. Differentially expressed genes (DEGs) were defined as those with |log_2_FC| > 0.25 and a false discovery rate (FDR)-adjusted *P*-value < 0.05. GSEA was performed using the clusterProfiler R package, and mean expression values were calculated using Seurat’s AverageExpression function. Pyroptosis, mitochondrial respiration, and glycolysis pathway activities were quantified using the AUCell package. Gene sets for pyroptosis, mitochondrial respiration, and glycolysis were obtained from MSigDB (Supplementary Table 5).

### Adeno-associated virus (AAV) construction and transfection

The pAAV/D374Y-hPCSK9 (*PCSK9*^*DY*^) plasmid, driven by the ApoEHCR-hAAT promoter, was generously provided by Dr. Bentzon (Addgene plasmid #58379). To produce AAV8 particles (AAV-*PCSK9*^*DY*^), the *PCSK9*^*DY*^ plasmid was co-transfected into HEK293T cells together with the pAAV2/8 trans-plasmid, which contains the AAV rep and cap genes, and the pAAV helper plasmid. Viral titers were determined by PCR using vector-specific primers. AAV-*PCSK9*^*DY*^ (2 × 10^11^ vector genomes per mouse) was administered to mice by a single tail-vein injection. At the time of AAV injection, mice were simultaneously placed on either a chow diet or a Western diet for 6 or 8 weeks.

To generate mice with VSMC-specific PDK4 overexpression, recombinant GFP-labeled AAV9 vectors containing either *PDK4* or an empty vector under the control of the smooth muscle 22α (*SM22α*) promoter (AAV9-*SM22α*-*PDK4*-GFP or AAV9-*SM22α*-empty-GFP) were produced by HanYi Biosciences Inc. (Guangzhou, China). The AAV9-*SM22α*-empty-GFP vector served as a negative control. These vectors (5 × 10^11^ genomic copies per mouse) were administered by intravenous injection. In the MCC950 treatment experiment, saline or MCC950 (MedChemExpress, CP-456773) was administered to mice by intraperitoneal injection every other day at a dose of 10 mg/kg. Two weeks after injection, PDK4 overexpression was confirmed by immunofluorescence and Western blot.

### Cell culture and transfection

Mouse aortic smooth muscle cells (MASMCs) were isolated from the abdominal aortas of 10-week-old wild-type, *Pdk4*^fl/fl^, and *Pdk4*^SMKO^ mice by collagenase digestion. The purity of MASMCs was verified by alpha-smooth muscle actin (ACTA2) immunofluorescence staining. Isolated MASMCs were cultured in high-glucose Dulbecco’s modified Eagle medium (DMEM; Gibco BRL, Grand Island, NY, USA) supplemented with 10% fetal bovine serum (FBS; VivaCell, Shanghai, China), 100 U/mL penicillin, and 100 μg/mL streptomycin at 37 °C in a humidified incubator with 5% CO_2_. MASMCs from passages 4–8 were used for subsequent experiments. For in vitro experiments, MASMCs at 80% confluence were serum-starved for 48 h in DMEM containing 1% penicillin/streptomycin-glutamine. The PI3K inhibitor LY294002 was purchased from MedChemExpress (HY-10108).

To isolate bone marrow-derived macrophages (BMDMs), bone marrow cells were collected from the tibiae and femurs of 8–12-week-old mice. The cells were cultured in DMEM supplemented with 10% FBS, 1% HEPES, 100 U/mL penicillin, 100 μg/mL streptomycin, and 30% L929-conditioned medium for 7 days as a source of macrophage colony-stimulating factor (M-CSF; Invitrogen, Carlsbad, CA, USA) to facilitate BMDM differentiation. To induce pyroptosis, BMDMs were primed with LPS (100 ng/mL) for 4 h, followed by stimulation with nigericin (10 μM) for 30 min in serum-free medium. All cells were maintained at 37 °C in a humidified incubator with 5% CO_2_.

Human monocyte-derived macrophages (HMDMs) were isolated from peripheral blood mononuclear cells (PBMCs) obtained from healthy volunteer donors using Percoll density-gradient centrifugation. PBMCs were then cultured in RPMI-1640 medium supplemented with 10% FBS, 1% HEPES, 100 U/mL penicillin, 100 μg/mL streptomycin, and 100 ng/mL M-CSF for 6 days to induce differentiation into HMDMs.

Human aortic smooth muscle cells (HASMCs) were obtained from Procell (CP-H081). Human umbilical vein endothelial cells (HUVECs) and AC16 cells were obtained from the Cell Bank of the Chinese Academy of Sciences (PSC-01; SCSP-555). HASMCs and AC16 cells were cultured in high-glucose DMEM supplemented with 10% FBS, 100 U/mL penicillin, and 100 μg/mL streptomycin at 37 °C in a humidified incubator with 5% CO_2_. HUVECs were cultured in RPMI-1640 medium supplemented with 10% FBS, 100 U/mL penicillin, and 100 μg/mL streptomycin at 37 °C in a humidified incubator with 5% CO_2_.

PDK4 knockdown was performed by transfecting cells with an siRNA targeting PDK4 (siRNA-*PDK4*; Sigma-Aldrich; sense: 5′-AGGTGGAGCATTTCTCGCGCTA-3′, antisense: 5′-GAATGTTGGCGAGTCTCACAGG-3). A scrambled siRNA (Sigma-Aldrich) was used as a negative control. siRNA transfection was performed using HiPerFect Transfection Reagent (Qiagen) according to the manufacturer’s instructions.

### Plasmid construction and transfection

The nucleotide sequence encoding PDK4 was amplified by PCR using the following primers: forward, 5′-AGGGAGGTCGAGCTGTTCTC-3′; reverse, 5′-GGAGTGTTCACTAAGCGGTCA-3′. The PCR products were digested and cloned into the pEnCMV vector containing a 3× HA tag. The construction of all plasmids was confirmed by DNA sequencing. Plasmid transfection was performed using Lipofectamine 2000 reagent (Invitrogen) according to the manufacturer’s instructions.

### Ultrasound

Ultrasonic imaging was performed using a Vevo 2100 ultrasound system (VisualSonics, ON, Canada) equipped with a 40-MHz transducer. Mice were anesthetized with 2% isoflurane and placed in the supine position on a 37 °C platform. Images of the abdominal aorta were obtained, and cine loops of 300 frames were recorded for measurement of aortic lumen diameter. The maximum abdominal aortic diameter was measured during diastole in B-mode imaging, and all measurements were performed by a blinded operator to minimize bias. All ultrasound data were analyzed statistically.

### Histological examinations

After sacrifice, mice were perfused with saline through the left ventricle. Abdominal aortic tissues were dissected, fixed in 4% paraformaldehyde, embedded in OCT compound, and sectioned. The sections were then stained with hematoxylin and eosin (H&E) or Verhoeff–Van Gieson (VVG) according to the manufacturer’s instructions.

For H&E staining, the sections were immersed in hematoxylin staining solution for 5–10 min, rinsed with distilled water to remove excess stain, differentiated in hydrochloric acid ethanol for 2 s, transferred quickly to distilled water for 2–3 min, and then stained with eosin staining solution for 30 s to 2 min. The sections were dehydrated sequentially in 70%, 80%, 95%, 100% ethanol I, and 100% ethanol II, each for 30 s, followed by two changes of xylene for 5 min each. Finally, the sections were cleared and mounted with synthetic resin.

For Verhoeff–Van Gieson staining, the sections were immersed in the working solution for 5 min and then rinsed with distilled water to remove excess dye. The sections were differentiated until the elastic fibers appeared black and the background became gray-white or colorless. Next, the sections were stained with Van Gieson solution for 1–3 min, rinsed briefly with distilled water, and rapidly dehydrated in three changes of absolute ethanol. The sections were then immersed in two changes of fresh xylene for 20 s and 5 min, respectively, for clearing, air-dried, and mounted with synthetic resin. Elastic fiber fragmentation was scored on a scale of 1–4 (1 = none, 2 = minimal, 3 = moderate, 4 = severe or rupture). All images were captured using an Olympus BX63 microscope (Olympus, Tokyo, Japan).

### Immunofluorescence

OCT-embedded abdominal aortic sections and paraformaldehyde-fixed cells were permeabilized with 0.1% Triton X-100, blocked with 5% BSA at room temperature, and then incubated with primary antibodies overnight at 4 °C. After washing with PBS, the sections or cells were incubated with secondary antibodies. Nuclei were counterstained with 4′,6-diamidino-2-phenylindole (DAPI). The antibodies used in this study were as follows: PDK4 (1:50 dilution; 12949-1-AP), ACTA2 (1:100 dilution; 67735-1-Ig), CNN1 (1:50 dilution; 24855-1-AP), SM22α (1:200 dilution; 10493-1-AP), NLRP3 (1:200 dilution; 30109-1-AP), and GSDMD (1:50 dilution; 20770-1-AP) (Proteintech). Immunofluorescence images of cells and tissues were acquired using a confocal fluorescence microscope (LSM 980, Carl Zeiss, Oberkochen, Germany).

### RNA sequencing

Total RNA was extracted from MASMCs using TRIzol reagent (Invitrogen Life Technologies) according to the manufacturer’s instructions. RNA integrity was evaluated using the RNA Nano 6000 Assay Kit on an Agilent Bioanalyzer 2100 system (Agilent Technologies). Library preparation was performed by Novogene (Beijing, China). For RNA sequencing, 1 μg of total RNA was used as input material for library construction using the NEBNext® Ultra™ RNA Library Prep Kit for Illumina® (New England Biolabs), according to the manufacturer’s instructions. Library quality was assessed using the Agilent Bioanalyzer 2100 system. Libraries were sequenced using 150-bp paired-end reads on an Illumina HiSeq 2500 platform. Differential expression analysis was performed using the DESeq2 R package. Genes with a *P*-value < 0.05 and |fold change| ≥ 2 were considered differentially expressed. Multiple-testing correction was performed using the Benjamini–Hochberg method. Kyoto Encyclopedia of Genes and Genomes (KEGG) pathway enrichment analysis of differentially expressed genes was performed using the clusterProfiler R package, with statistical significance set at *P* < 0.05.

### Quantitative reverse transcription polymerase chain reaction (RT-qPCR)

Total RNA was extracted from abdominal aortic tissues and VSMCs using TRIzol reagent (Ambion, USA). cDNA was synthesized from total RNA using Evo M-MLV RT Master Mix (Agbio, Hunan, China). Gene expression was analyzed using a LightCycler 480 real-time PCR system (Roche, Indianapolis, IN, USA). qPCR reactions were performed using SYBR Green Premix (Agbio, Hunan, China) according to the manufacturer’s instructions. Relative gene expression was calculated using the 2^−^^ΔΔCt^ method, with β-actin as the internal control. Primer sequences are listed in Supplementary Table 3.

### SNP genotyping for confirmation of genetic background

Genomic DNA was extracted from tail biopsies of *Pdk4*^fl/fl^ and *Pdk4*^SMKO^ mice. To verify that the two genotypes shared a uniform C57BL/6J background, SNP genotyping was performed on mice from multiple independent litters within the same breeding colony. PCR amplification of strain-informative SNP loci was followed by Sanger sequencing. Primers flanking each SNP locus were designed using Primer3, and PCR products were resolved on a 1.5% agarose gel and purified. Sequencing was performed using BigDye® Terminator v3.1 chemistry on an ABI 3730xl sequencer. The obtained sequences were aligned to the C57BL/6J reference genome to confirm allele identity and exclude mixed-background contamination.

### Assessment of mtDNA

Total DNA was extracted from MASMCs using the PureLink™ Genomic DNA Kit (Thermo Fisher Scientific, Carlsbad, CA, USA). mtDNA content was quantified by qPCR using primers specific for the mitochondrial ATP6 gene, with GAPDH used as the nuclear DNA (nDNA) reference. qPCR was performed on a LightCycler 480 real-time PCR system (Roche, Indianapolis, IN, USA) using SYBR Green Premix (Agbio, Hunan, China). Relative mtDNA content was calculated as the ratio of mtDNA to nDNA (mtDNA/nDNA). Primer sequences are listed in Supplementary Table 3.

### JC-1 staining

JC-1 staining was performed using a mitochondrial membrane potential detection kit (C2006, Beyotime Biotechnology). MASMCs and OCT-embedded abdominal aortic sections that were not fixed in 4% paraformaldehyde were permeabilized in PBS containing 0.1% Triton X-100 for 5 min. Samples were then incubated with the JC-1 working solution for 1 h. During this period, 1× JC-1 staining buffer was prepared by diluting 1 mL of JC-1 buffer (5×) with 4 mL of distilled water and kept on ice. After incubation, samples were washed twice with 1× JC-1 buffer and then incubated with DAPI for 5 min. The samples were mounted with anti-fade medium, and images were acquired using a confocal fluorescence microscope (LSM 980, Carl Zeiss, Oberkochen, Germany).

### Protein extraction and Western blot

Proteins were extracted from cells and tissues using RIPA lysis buffer supplemented with protease inhibitors (Merck, Darmstadt, Germany) and quantified using a BCA protein assay kit (Thermo Fisher Scientific, Waltham, MA, USA). Equal amounts of protein were separated on 6–12% SDS–PAGE gels and transferred onto polyvinylidene difluoride (PVDF) membranes (Millipore, Billerica, MA, USA). Membranes were blocked with 5% non-fat milk for 1 h at room temperature and incubated with primary antibodies overnight at 4 °C. The following primary antibodies were used: PDK4 (12949-1-AP, 1:2000), PDK1 (18262-1-AP, 1:1000), PDK2 (15647-1-AP, 1:500), PDK3 (12215-1-AP, 1:500), ACTA2 (67735-1-Ig, 1:20,000), CNN1 (24855-1-AP, 1:2000), SM22α (10493-1-AP, 1:5000), α-Tubulin (66031-1-Ig, 1:20,000), TXNIP (18243-1-AP, 1:500), and TRX (14999-1-AP, 1:1000) from Proteintech; KLF4 (ab215036, 1:1000), KLF5 (ab137676, 1:500), GAPDH (ab8245, 1:500), NLRP3 (ab263899, 1:100), p-PDHE1α (ab177461, 1:1000), and PDHE1α (ab168379, 1:1000) from Abcam; cleaved caspase1 (#89332, 1:1000), cleaved IL-1β (#63124, 1:1000), GSDMD (#39754, 1:1000), and N-GSDMD (#10137, 1:1000) from Cell Signaling Technology; and cleaved IL-18 (A24057, 1:1000) from Abclonal. After washing with TBST, membranes were incubated with the appropriate secondary antibodies. Signals were detected using an enhanced chemiluminescence (ECL) kit (Thermo Fisher Scientific), and band intensities were quantified using ImageJ software (v1.8.0).

### Immunoprecipitation

Immunoprecipitation assays were performed using cell lysates normalized to 500 μg of total protein. Lysates were incubated overnight at 4 °C with either anti-TXNIP antibody (18243-1-AP; Proteintech) or rabbit IgG isotype control (30000-0-AP; Proteintech), followed by precipitation with Protein A/G PLUS-Agarose (sc-2003; Santa Cruz Biotechnology). After washing the beads with ice-cold PBS, immunoprecipitates were separated on 10% SDS–PAGE gels and transferred onto PVDF membranes (Millipore, Billerica, MA, USA). Membranes were blocked with 5% non-fat milk for 1 h at room temperature and incubated with primary antibodies overnight at 4 °C. The following primary antibodies were used: TXNIP (18243-1-AP, 1:500) and TRX (14999-1-AP, 1:1000) from Proteintech, and NLRP3 (ab263899, 1:100) from Abcam. After washing with TBST, membranes were incubated with the appropriate secondary antibodies. Signals were detected using an enhanced chemiluminescence (ECL) kit (Thermo Fisher Scientific), and band intensities were quantified using ImageJ software (v1.8.0).

### Blood pressure

Baseline blood pressure was measured using a tail-cuff system. Each mouse underwent at least three consecutive measurements, and the mean value was used for analysis.

### Serum analysis

Following an overnight fast, blood samples were collected from mice anesthetized with intraperitoneal pentobarbital sodium before sacrifice. Total serum cholesterol, triglyceride, low-density lipoprotein, and high-density lipoprotein levels were measured using a fully automated clinical chemistry analyzer (Chemray800, Rayto Life and Analytical Sciences Co., Ltd., Shenzhen, China).

### Enzyme-linked immunosorbent assay (ELISA)

Serum levels of ASC, IL-1β, IL-18, TNF-α, and IL-6 in *Pdk4*^SMKO^ and *Pdk4*^fl/fl^ mice were measured using ELISA kits according to the manufacturer’s instructions. ELISA kits for mouse ASC, IL-1β, IL-18, TNF-α, and IL-6 were purchased from Abcam Biotechnology Co., Ltd. For measurement of serum ASC levels, blood was collected, and serum was separated by centrifugation. Serum samples and ASC standards were added to 96-well plates pre-coated with capture antibody. After incubation, detection was performed using a biotinylated anti-ASC antibody, streptavidin-HRP conjugate, and TMB substrate. Absorbance was measured at 450 nm, and ASC concentrations were calculated based on a standard curve.

### Mitochondrial reactive oxygen species (ROS) detection

Cells were seeded in confocal dishes and cultured to 50–60% confluence. The culture medium was removed, and cells were washed with PBS and then incubated with 5 μM MitoSOX™ Red (M36008, Invitrogen) in serum-free medium for 30 min at 37 °C in the dark. Cells were then thoroughly washed to remove excess MitoSOX™ Red and imaged using a confocal fluorescence microscope (LSM 980, Carl Zeiss, Oberkochen, Germany) with excitation at 396 nm and emission at 610 nm. Fresh frozen abdominal aortic sections without prior 4% formaldehyde fixation were stained with 5 μM MitoSOX™ Red in PBS at room temperature in the dark for 30 min and then imaged using the same confocal fluorescence microscope.

### TUNEL staining

TUNEL staining was performed using a one-step TUNEL apoptosis detection kit (C1089, Beyotime Biotechnology). Fresh frozen abdominal aortic sections were fixed with 4% paraformaldehyde for 30 min, followed by two washes with PBS for 5 min each. The sections were then treated with autofluorescence quenching reagent at room temperature for 5 min, followed by incubation with TUNEL detection solution at 37 °C in the dark for 90 min. DAPI staining was performed for 15 min, and the sections were mounted with anti-fade mounting medium. Images were acquired using a confocal fluorescence microscope (LSM 980, Carl Zeiss, Oberkochen, Germany).

### LDH release assay

To assess cell cytotoxicity, an LDH release assay was performed using an LDH Cytotoxicity Detection Kit (#K313-500, BioVision, USA) according to the manufacturer’s instructions. Briefly, MASMCs were seeded in 96-well plates and treated with Ang II (1 μM) for 48 h. After treatment, 100 μL of culture supernatant from each well was transferred to a new 96-well plate. An equal volume of LDH reaction mixture was added to each well, and the plate was incubated at room temperature for 30 min in the dark. Absorbance was measured at 450 nm using a TECAN Infinite M200 microplate reader.

### Transmission electron microscopy

After euthanasia, abdominal aortic tissues were rapidly dissected into small pieces, with no dimension (length, width, or height) exceeding 1 mm. The tissue samples were immediately immersed in 2.5% glutaraldehyde fixative for preservation. For MASMCs, the culture medium was removed, and the cells were fixed with 2.5% glutaraldehyde for 5 min at room temperature. The cells were then collected by low-speed centrifugation and resuspended in fresh 2.5% glutaraldehyde fixative for storage at 4 °C. Fixed samples were processed and imaged by PINUOFEI Biotech (Wuhan, China).

### Cellular oxygen consumption rate (OCR) detection

Mouse aortic smooth muscle cells (MASMCs) were seeded in Seahorse XFe96 cell culture microplates at a density of 5000 cells per well and cultured overnight to allow adherence. On the day of the assay, the culture medium was replaced with Seahorse XF assay medium (pH 7.4) supplemented with 2 mM glutamine, 1 mM pyruvate, and 10 mM glucose. Cells were then incubated at 37 °C in a non-CO_2_ incubator for 1 h. Mitochondrial respiration was assessed using a Seahorse XFe96 Extracellular Flux Analyzer (Agilent Technologies) according to the manufacturer’s instructions. OCR was measured under basal conditions and after sequential injection of oligomycin (1.5 μM), FCCP (1 μM), and rotenone/antimycin A (0.5 μM each). OCR data were analyzed using Seahorse Wave software (Agilent Technologies), and basal respiration, ATP-linked respiration, maximal respiration, and spare respiratory capacity were calculated. OCR values were normalized to protein content.

### Proton efflux rate (PER) detection

MASMCs were seeded in Seahorse XFe96 cell culture microplates at a density of 5,000 cells per well and incubated overnight to allow adherence. The Seahorse XF sensor cartridge was hydrated overnight in Seahorse XF calibrant at 37 °C in a non-CO₂ incubator. On the day of the assay, the culture medium was replaced with Seahorse XF assay medium supplemented with 1 mM sodium pyruvate, 2 mM glutamine, and 10 mM glucose, and cells were equilibrated at 37 °C in a non-CO₂ incubator for 1 h. The assay was performed using a Seahorse XFe96 Extracellular Flux Analyzer (Agilent Technologies) according to the manufacturer’s instructions. PER was measured under basal conditions and after sequential injection of rotenone/antimycin A (0.5 μM) and 2-deoxyglucose (2-DG) (50 μM) using the Seahorse XF Glycolytic Rate Assay Kit (103344-100, Agilent Technologies, USA). Data were analyzed using Wave software (Agilent Technologies), and basal glycolysis and compensatory glycolysis were calculated. OCR/PER values were normalized to the total protein content of each well.

### Metabolite measurements

MASMCs were cultured in 10-cm dishes until they reached 80% confluence and were then harvested for metabolite measurements. PDH activity was measured using the PDH Activity Colorimetric Assay Kit (ab287837, Abcam) according to the manufacturer’s instructions. Absorbance at 450 nm was measured kinetically for approximately 30 min at 37 °C after addition of the PDH substrate and developer. Acetyl-CoA levels were measured using the PicoProbe Acetyl-CoA Fluorometric Assay Kit (K317-100, BioVision) according to the manufacturer’s protocol. Pyruvate levels were measured using the Pyruvate Assay Kit (ab65342, Abcam) according to the manufacturer’s protocol.

### Statistical analysis

All statistical analyses were performed using GraphPad Prism (version 9.0; GraphPad Software, La Jolla, CA, USA). Quantitative data are presented as mean ± standard deviation (SD). Data normality was assessed using the Shapiro–Wilk test. For data that passed the normality test, comparisons between two groups were performed using a two-tailed unpaired Student’s *t*-test, whereas comparisons among three or more groups were performed using one-way or two-way analysis of variance (ANOVA), followed by Dunnett’s or Tukey’s multiple-comparisons test. For non-normally distributed data, comparisons between two groups were performed using the nonparametric Mann–Whitney *U*-test. Statistical significance was defined as *P* < 0.05.

### Reporting summary

Further information on research design is available in the Nature Portfolio Reporting Summary linked to this article.

## Supplementary information

Supplementary Information

Peer Review File

Reporting Summary

## Source data

Source data

## Data Availability

The RNA sequencing data generated in this study have been deposited in the Gene Expression Omnibus (GEO) database under accession codes GSE269546 and GSE305105. Previously published datasets analyzed in this study are available in GEO under accession number GSE239620^70^. All data supporting the findings of this study are included in the main text, the Supplementary Information, or the Source Data file. Source data are provided with this paper.
