# Supplementary material for: PDK4 drives abdominal aortic aneurysm by promoting smooth muscle cell metabolic reprogramming and NLRP3-mediated pyroptosis
Source: Nat Commun. 2026 Apr 11;17:5086. doi: 10.1038/s41467-026-71610-w (PMC13246965; doi:10.1038/s41467-026-71610-w)
Supplement: Supplementary file 1 — Supplementary Information [file 41467_2026_71610_MOESM1_ESM.pdf]

Supplementary Information

PDK4 drives abdominal aortic aneurysm by promoting smooth muscle cell metabolic reprogramming and NLRP3-mediated pyroptosis

Li Zhao (赵力)[1, †]; Xuefeng Lin[1, †]; Zhengqiang Zhu[1, †]; Ranxin Liu[1, †]; Lingna Zhao[1]; Xuekun Wu[2]; Mengru Zheng[1]; Rihua Huang[3]; Pengyu Zhou[1]; Fangze Huang[1]; Deshen Liu[1]; Chuanjie Niu[1]; Xiaoxia He,[1]; Zean Wang[1]; Xin Li[4]; Jiale Li[1]; Shengping He[1]; Jun Lu[1]; Shaoyi Zheng (郑少忆)[1, *]; Jiaguo Zhou (周家国)[5, *]; Qinbao Peng (彭勤宝)[1, *]; Xiu Liu (刘秀)[1, 6 *]

[1]Department of Cardiovascular Surgery, Nanfang Hospital, Southern Medical University, Guangzhou, 510515, China.

[2]Stanford Cardiovascular Institute, Stanford University School of Medicine, Stanford, CA 94305, USA.

[3]Department of Cardiology, The First Affiliated Hospital, Sun Yat-Sen University, Guangzhou, 510080, China.

[4]Department of Cardiac Surgery, Guangdong Provincial People's Hospital (Guangdong Academy of Medical Sciences), Southern Medical University, Guangzhou, 510080, China.

[5]Department of Pharmacology, Cardiac and Cerebral Vascular Research Center, Zhongshan School of Medicine, Sun Yat-sen University, Guangzhou, 510080, China.

[6]Guangdong Provincial Key Laboratory of Single-cell and Extracellular Vesicles, Southern Medical University, Guangzhou, 510515, China.

[†]These authors contributed equally to this work.

*Corresponding authors

Xiu Liu: liux266@smu.edu.cn; Qinbao Peng: sharp1986@smu.edu.cn; Jiaguo Zhou: zhoujg@mail.sysu.edu.cn; Shaoyi Zheng: zhsy@smu.edu.cn.

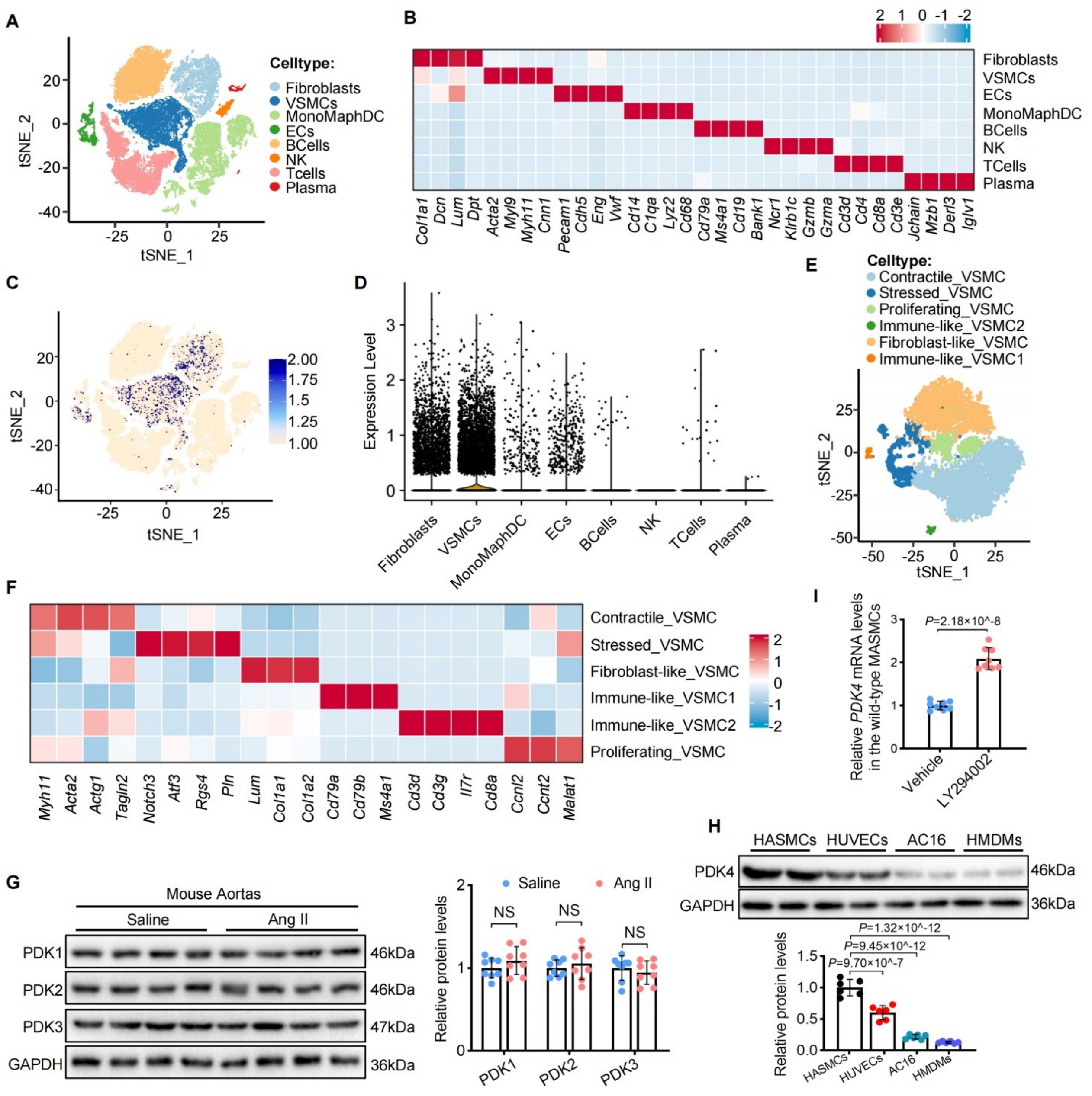

Fig. S1 | PDK4 is the predominant dysregulated isoform in AAA tissue. A t-distributed stochastic neighbor embedding (*t*-SNE) plot displaying all cells from mouse aortas, colored according to the eight identified cell types (Gene Expression Omnibus accession no. GSE239620). **B** Mean expression of marker genes across major cell types. **C** t-SNE plots showing single-cell gene expression of *PDK4* across cell clusters from the GEO data. **D** Violin plot of *PDK4* expression levels across different cell groups. **E** *t*-SNE visualization of VSMC subclusters with color-coded classification. **F** Mean expression of marker genes across six VSMC subclusters. **G** Relative PDK1, PDK2, and PDK3 protein

levels were measured by Western blot analysis in the abdominal aortas of mice treated with AAV-*PCSK9*^{DY}/Ang II for 28 days or AAV-*PCSK9*^{DY}/saline for 28 days (n=8 biological replicates). **H** Relative PDK4 protein levels in different cells, including HASMCs, HUVECs, AC16, and HMDMs (n=6 biological replicates). **I** Relative *PDK4* mRNA levels in MASMCs isolated from wild-type mice treated with vehicle or LY294002 (50 μM, 48 h) (n=8 biological replicates). Data are presented as mean ± SD. (G, I) Two-sided Unpaired Student's *t*-test; (H) one-way ANOVA followed by Tukey's multiple-comparisons test. Source data are provided as a Source Data file.

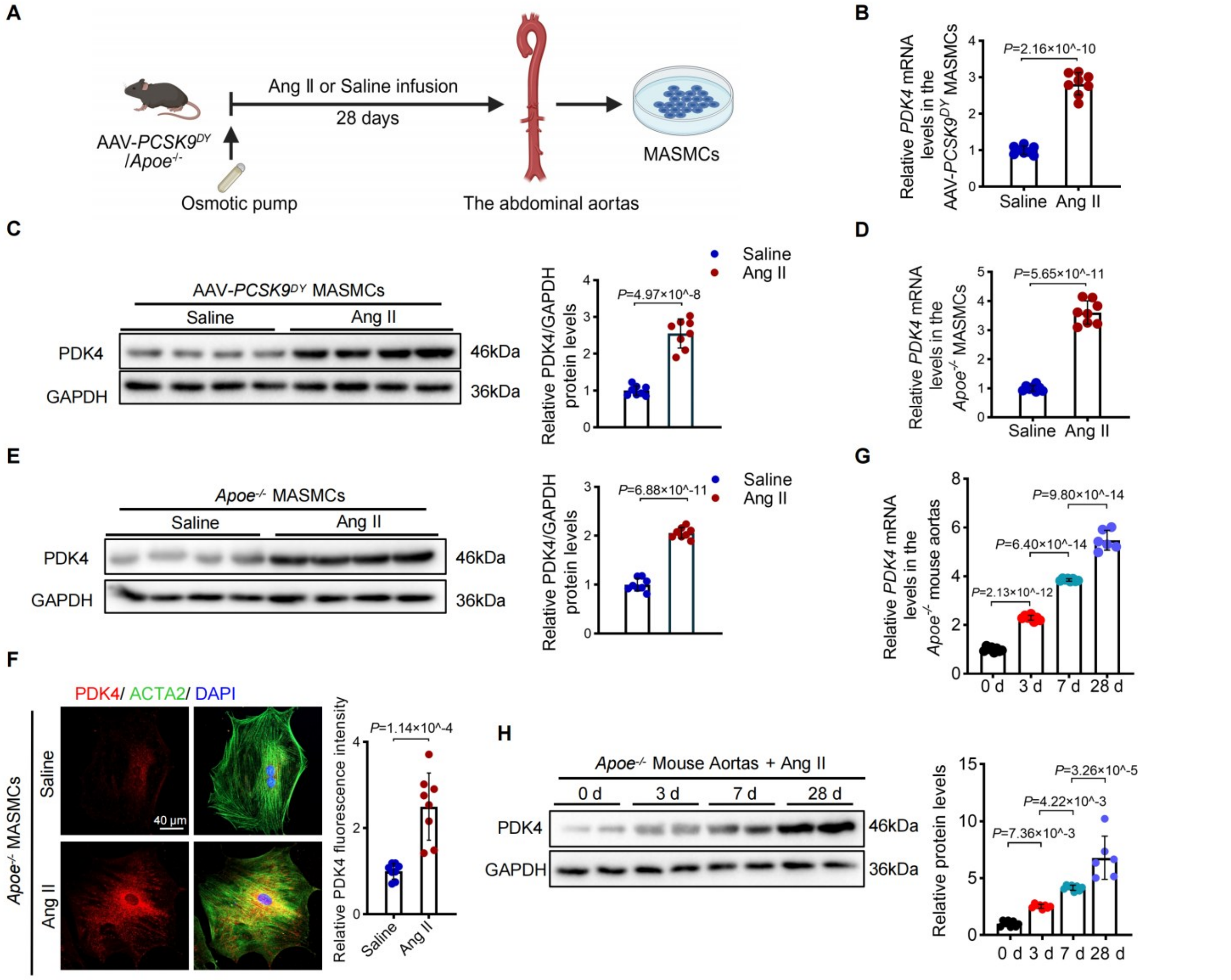

Fig. S2 | PDK4 is upregulated in MASMCs with Ang II. A Schematic diagram of the extraction method and treatment for MASMCs from AAV-*PCSK9*^{DY} or *Apoe*$^{-/-}$ mice. Created with BioRender. Liu, X. (2026) https://BioRender.com/po5y0ge. **B-C** Relative PDK4 mRNA and protein levels in MASMCs isolated from AAV-*PCSK9*^{DY} mice treated with Ang II or saline for 28 days (n=8 biological replicates). **D-E** Relative PDK4 mRNA and protein levels in MASMCs isolated from *Apoe*$^{-/-}$ mice treated with Ang II or saline for 28 days (n=8 biological replicates). **F** PDK4 and ACTA2 immunofluorescence in MASMCs isolated from *Apoe*$^{-/-}$ mice treated with Ang II or saline for 28 days (n=8 biological replicates). **G-H** Relative PDK4 mRNA and protein levels were measured by RT-qPCR and Western blot analysis in the abdominal aortas of *Apoe*$^{-/-}$ mice at different time points (0, 3,

7 and 28 days) after Ang II treatment (n=8 biological replicates for 0, 3, 7 days; n=6 biological replicates for 28 days). Data are presented as mean ± SD. (B-F) Two-sided Unpaired Student's *t*-test; (G, H) one-way ANOVA followed by Tukey’s multiple-comparisons test. Source data are provided as a Source Data file.

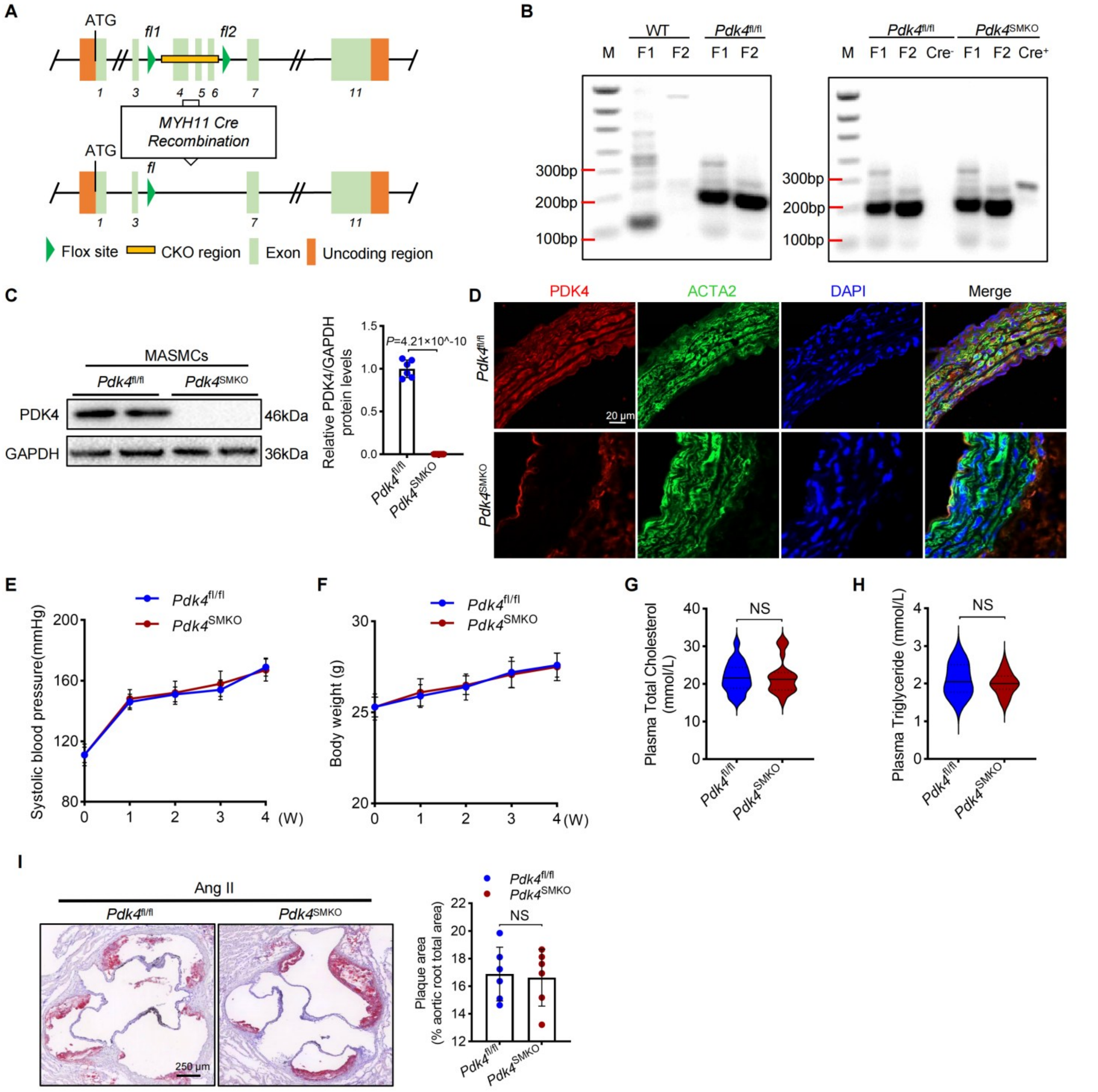

Fig. S3 | Construction and validation of the VSMC-specific PDK4 knockout mouse model. A Strategy for generation of *Pdk4*^{SMKO} mice. **B** Mice were PCR-genotyped using specific primers for fl1 (F1), fl2 (F2), or *Myh11-Cre*. Mice harboring fl1, fl2, and *Mhy11-Cre* were considered *Pdk4*^{SMKO}; those harboring fl1 and fl2, but not *Myh11-Cre*, were considered *Pdk4*$^{\text{fl/fl}}$. **C** Western blot analysis showing the PDK4 protein levels in VSMCs isolated from *Pdk4*$^{fl/fl}$ and *Pdk4*^{SMKO} mice (n=6 biological replicates). **D** Representative images of PDK4 and ACTA2 expression using immunofluorescence

staining in suprarenal abdominal aortas from mice of *Pdk4*$^{fl/fl}$ and *Pdk4*^{SMKO} (n=6 biological replicates). **E-H** Systolic blood pressure, body weight, and serum TC and TG levels in *Pdk4*$^{fl/fl}$ and *Pdk4*^{SMKO} mice treated with AAV-*PCSK9*^{DY}/Ang II (n=18 for *Pdk4*$^{fl/fl}$ and n=22 for *Pdk4*^{SMKO}, mice that died of aortic rupture were not included in the measurements). **I** Representative Oil Red O-stained aortic root sections and quantification in *Pdk4*$^{fl/fl}$ and *Pdk4*^{SMKO} mice (n=6 biological replicates). Data are presented as mean ± SD. (G) Two-sided Mann–Whitney *U* test (exact method); (C, E-F, H-I) Two-sided Unpaired Student's *t*-test. NS, nonsignificant. Source data are provided as a Source Data file.

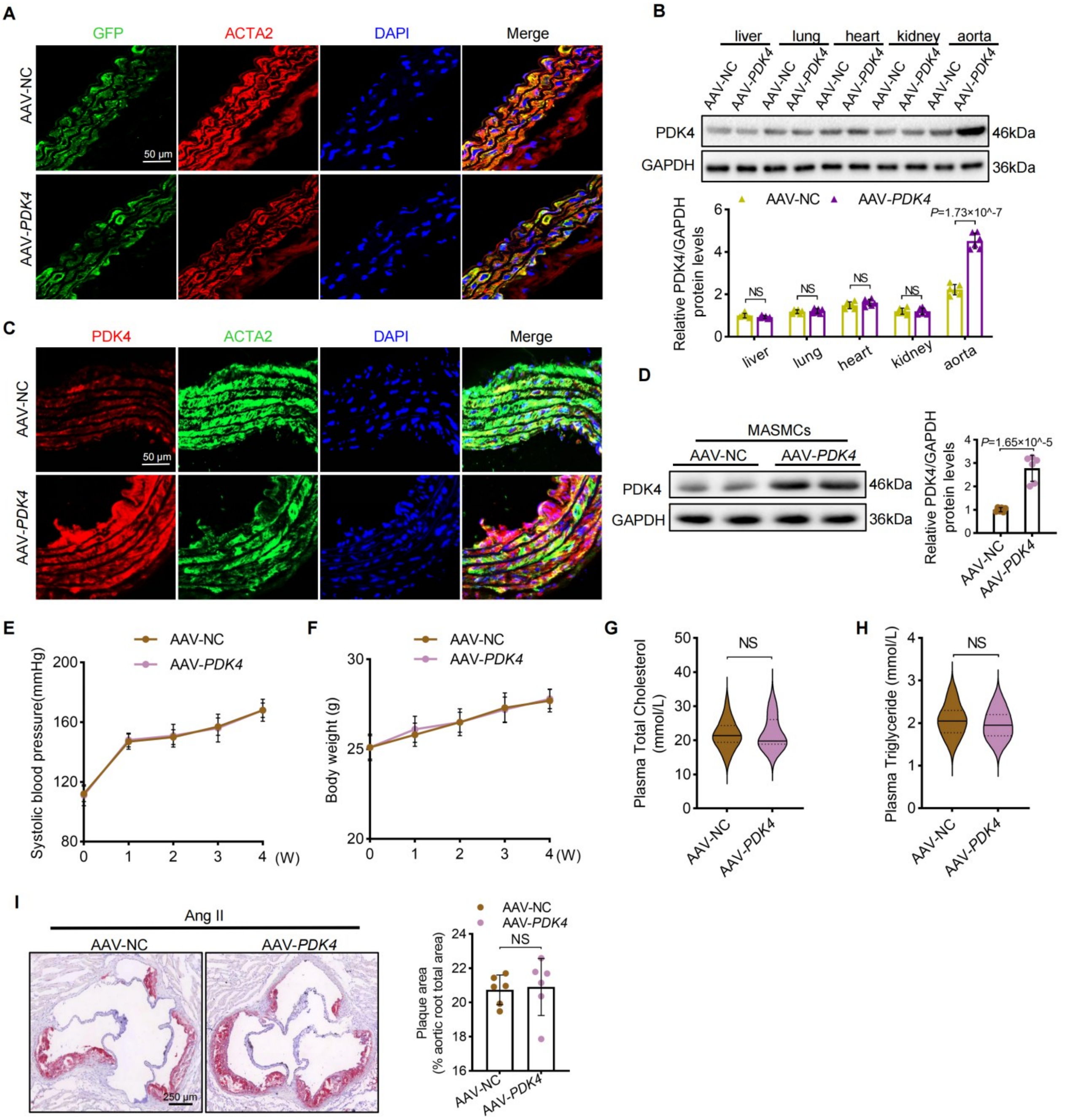

Fig. S4 | Validation of the VSMC-specific PDK4 overexpression mouse model. A Representative immunofluorescence images showing green fluorescent protein (GFP) and ACTA2 expression in suprarenal abdominal aortas from mice injected with AAV-NC or AAV-*PDK4* (n=6 biological replicates). **B** PDK4 protein levels in the liver, lung, heart, kidney, and aorta of AAV-NC and AAV-*PDK4* mice (n=6 biological replicates). **C** Representative immunofluorescence staining of PDK4 and ACTA in suprarenal abdominal aortas from mice injected with AAV-NC and AAV-*PDK4* (n=6

biological replicates). **D** PDK4 protein levels in VSMCs isolated from AAV-NC and AAV-*PDK4* mice (n=6 biological replicates). **E-H** Systolic blood pressure, body weight, and serum TC and TG levels in AAV-NC and AAV-*PDK4* mice (n=18 for AAV-NC and n=12 for AAV-*PDK4*, mice that died from aortic rupture were excluded from these measurements). **I** Representative Oil Red O-stained aortic root sections and quantification in AAV-NC and AAV-*PDK4* mice (n=6 biological replicates). Data are presented as mean ± SD. (B, D-F, H-I) Two-sided Unpaired Student's *t*-test; (G) Two-sided Mann–Whitney *U* test (exact method). NS, nonsignificant. Source data are provided as a Source Data file.

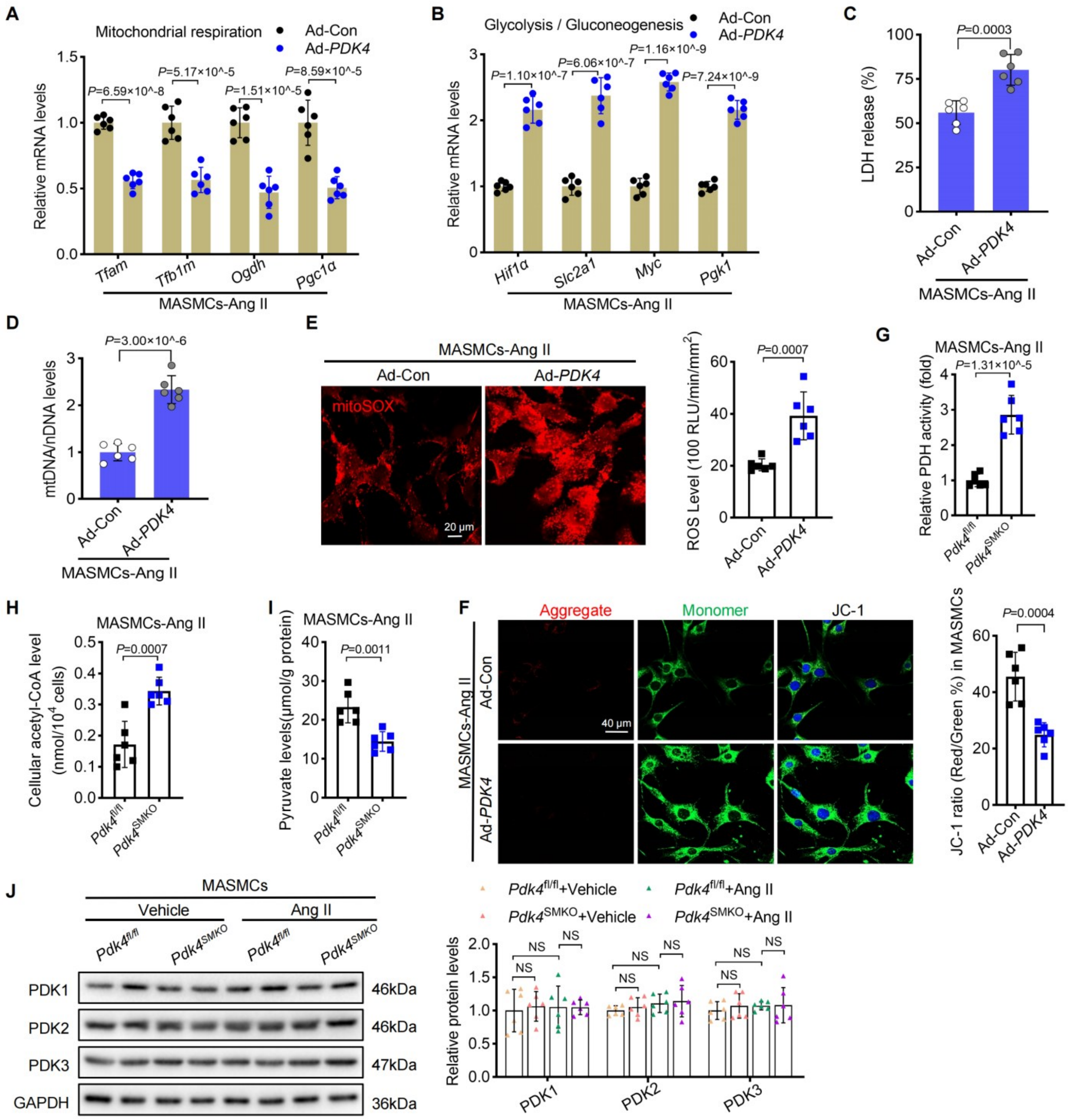

Fig. S5 | PDK4 overexpression alters mitochondrial respiration and ROS production. A-F MASMCs transfected with Ad-NC or Ad-*PDK4*, followed by treatment with Ang II (1 μM, 48 h). **A** Relative mRNA levels of genes related to mitochondrial respiration were measured by RT-qPCR analysis in the MASMCs (n=6 biological replicates). **B** Relative mRNA levels of genes related to glycolysis/gluconeogenesis were measured by RT-qPCR analysis in the MASMCs (n=6 biological replicates). **C** LDH levels in the MASMCs (n=6 biological replicates). **D** mtDNA/nDNA levels in the MASMCs (n=6 biological replicates). **E** Representative mitochondria-derived ROS fluorescence

probe (MitoSOX) staining in MASMCs (n=6 biological replicates). **F** Representative JC-1 staining and quantification in MASMCs (n=6 biological replicates). **G-I** Levels of PDH activity, acetyl-CoA and pyruvate in MASMCs isolated from *Pdk4*$^{fl/fl}$ and *Pdk4*^{SMKO} mice, followed by treatment with Ang II (1 μM, 48 h) (n=6 biological replicates). **J** Relative PDK1, PDK2, and PDK3 protein levels were measured by Western blot analysis in MASMCs isolated from *Pdk4*$^{fl/fl}$ and *Pdk4*^{SMKO} mice, followed by treatment with vehicle or Ang II (1 μM, 48 h) (n=6 biological replicates). Data are presented as mean ± SD. (A-I) Two-sided Unpaired Student's *t*-test. (J) two-way ANOVA followed by Tukey's multiple-comparisons test. NS, nonsignificant. Source data are provided as a Source Data file.

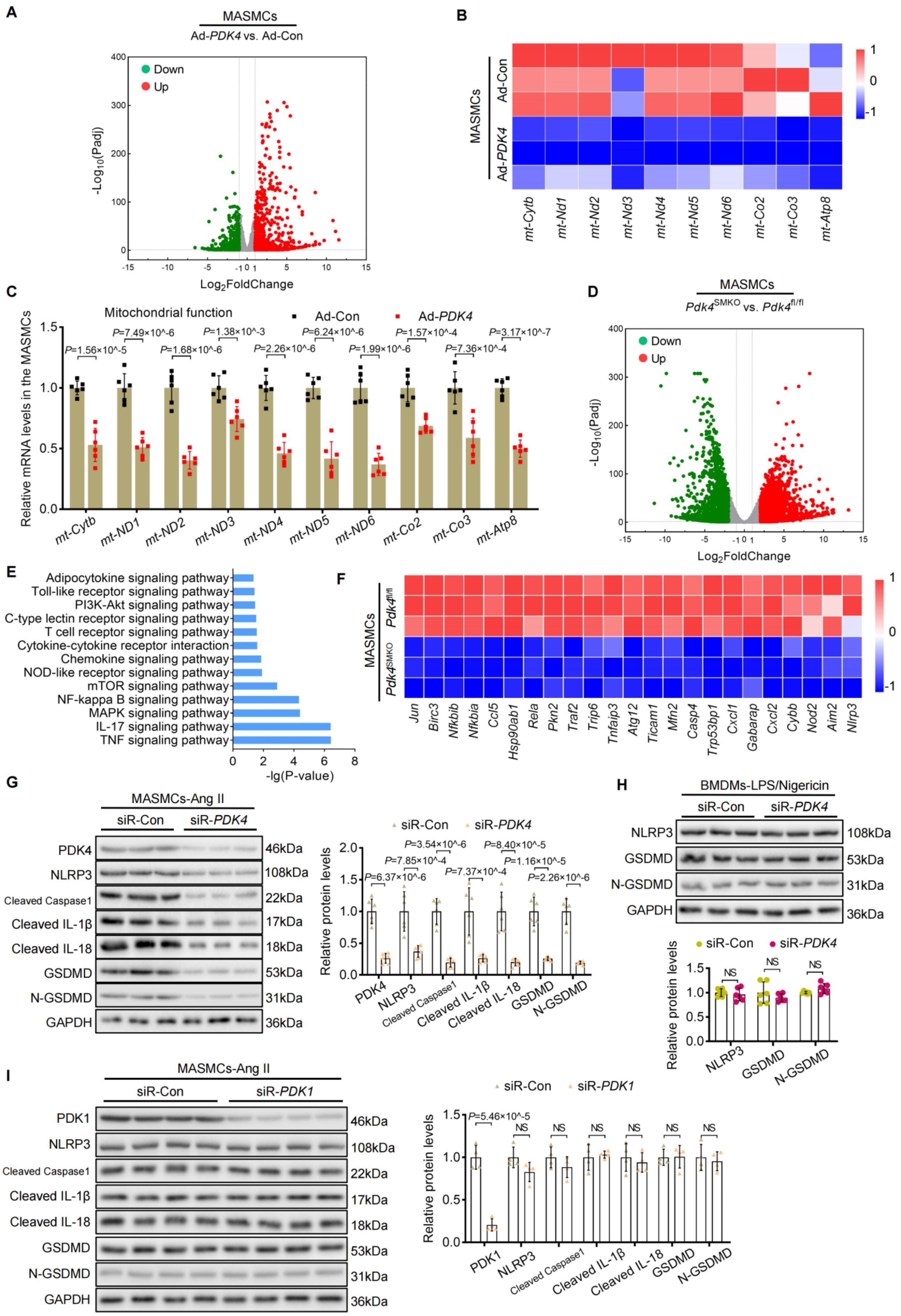
A
MASMCs
Ad-PDK4 vs. Ad-Con
Down
Up
-Log10(Padj)
Log2FoldChange
B
MASMCs
Ad-Con
Ad-PDK4
mt-Cytb
mt-Nd1
mt-Nd2
mt-Nd3
mt-Nd4
mt-Nd5
mt-Nd6
mt-Co2
mt-Co3
mt-Atp8
C
Mitochondrial function
Ad-Con
Ad-PDK4
Relative mRNA levels in the MASMCs
P=1.56×10^-5
P=7.49×10^-6
P=1.68×10^-6
P=1.38×10^-3
P=2.26×10^-6
P=6.24×10^-6
P=1.99×10^-6
P=1.57×10^-4
P=7.36×10^-4
P=3.17×10^-7
mt-Cytb
mt-ND1
mt-ND2
mt-ND3
mt-ND4
mt-ND5
mt-ND6
mt-Co2
mt-Co3
mt-Atp8
D
MASMCs
Pdk4SMKO vs. Pdk4fl/fl
Down
Up
-Log10(Padj)
Log2FoldChange
E
Adipocytokine signaling pathway
Toll-like receptor signaling pathway
PI3K-Akt signaling pathway
C-type lectin receptor signaling pathway
T cell receptor signaling pathway
Cytokine-cytokine receptor interaction
Chemokine signaling pathway
NOD-like receptor signaling pathway
mTOR signaling pathway
NF-kappa B signaling pathway
MAPK signaling pathway
IL-17 signaling pathway
TNF signaling pathway
-lg(P-value)
F
MASMCs
Pdk4fl/fl
Pdk4SMKO
Jun
Birc3
Nfkbib
Nfkbia
Ccl5
Hsp90ab1
Rela
Pkn2
Traf2
Trip6
Tnfaip3
Atg12
Ticam1
Mfn2
Casp4
Trp53bp1
Cxcl1
Gabarap
Cxcl2
Cybb
Nod2
Aim2
Nlrp3
G
MASMCs-Ang II
siR-Con
siR-PDK4
PDK4 46kDa
NLRP3 108kDa
Cleaved Caspase1 22kDa
Cleaved IL-1β 17kDa
Cleaved IL-18 18kDa
GSDMD 53kDa
N-GSDMD 31kDa
GAPDH 36kDa
Relative protein levels
P=6.37×10^-6
P=7.85×10^-4
P=3.54×10^-6
P=7.37×10^-4
P=8.40×10^-5
P=1.16×10^-5
P=2.26×10^-6
H
BMDMs-LPS/Nigericin
siR-Con
siR-PDK4
NLRP3 108kDa
GSDMD 53kDa
N-GSDMD 31kDa
GAPDH 36kDa
Relative protein levels
NS
I
MASMCs-Ang II
siR-Con
siR-PDK1
PDK1 46kDa
NLRP3 108kDa
Cleaved Caspase1 22kDa
Cleaved IL-1β 17kDa
Cleaved IL-18 18kDa
GSDMD 53kDa
N-GSDMD 31kDa
GAPDH 36kDa
Relative protein levels
P=5.46×10^-5
NS

Fig. S6 | Transcriptomic profiling and validation of PDK4-regulated pathways in MASMCs. A Volcano plots of the differentially expressed genes in MASMCs transfected with Ad-Con or Ad-*PDK4*, followed by treatment with Ang II (1 μM, 48 h) (n=3 biological replicates). **B** Heatmap comparing the gene expression profiles of MASMCs transfected with Ad-Con or Ad-*PDK4*, followed by treatment with Ang II (1 μM, 48 h) (blue indicates downregulation; red indicates upregulation). **C** Relative mRNA levels of mtDNA genes were measured by RT-qPCR analysis in MASMCs transfected with Ad-Con or Ad-*PDK4*, followed by treatment with Ang II (1 μM, 48 h) (n=6 biological replicates). **D** Volcano plots of the differentially expressed genes in MASMCs isolated from *Pdk4*^{SMKO} and *Pdk4*$^{fl/fl}$ mice and treated with Ang II (1 μM, 48 h) (n=3 biological replicates). **E** KEGG analysis of downregulated pathways identified by RNA-seq in MASMCs isolated from *Pdk4*^{SMKO} and *Pdk4*$^{fl/fl}$ mice after Ang II treatment (1 μM, 48 h) (n=3 biological replicates). **F** Heat map of RNA-seq data from MASMCs isolated from *Pdk4*^{SMKO} and *Pdk4*$^{fl/fl}$ mice and treated with Ang II (1 μM, 48 h) (n=3 biological replicates) (blue indicates downregulation; red indicates upregulation). **G** Representative Western blot and quantification of PDK4, NLRP3, Cleaved Caspase1, Cleaved IL-1β, Cleaved IL-18, GSDMD and N-GSDMD protein levels in MASMCs transfected with siR-Con or siR-*PDK4*, followed by treatment with Ang II (1 μM, 48 h) (n=6 biological replicates). **H** Representative Western blot and quantification of NLRP3, GSDMD and N-GSDMD protein levels in BMDMs transfected with siR-Con or siR-*PDK4*, followed by treatment with LPS (100 ng/mL, 4 h) and nigericin (10 μM, 30 min) (n=6 biological replicates). **I** Representative Western blot and quantification of PDK1, NLRP3, Cleaved Caspase-1, Cleaved IL-1β, Cleaved IL-18, GSDMD, and N-GSDMD protein levels in MASMCs transfected with siR-Con or siR-*PDK1*, followed by Ang II treatment (1 μM, 48 h) (n=6 biological replicates). Data are presented as mean ± SD. (C, G, H) Two-sided Unpaired Student's *t*-

test. (I) Two-sided Unpaired Student's *t*-test or Mann–Whitney *U* test (exact method). NS, nonsignificant. Source data are provided as a Source Data file.

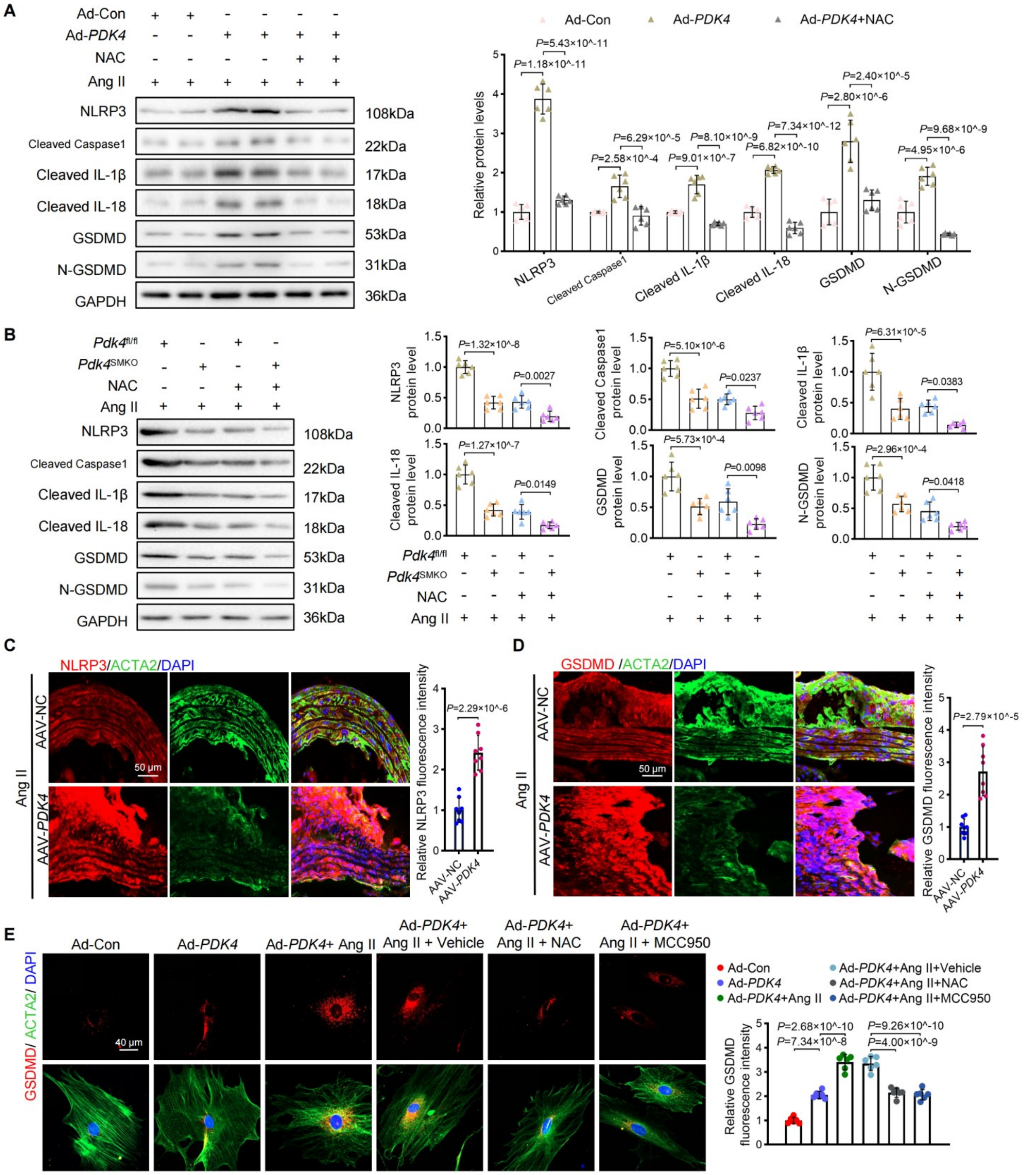

Fig. S7 | *PDK4* overexpression promotes pyroptosis in VSMCs. A MASMCs were serum-starved and simultaneously transfected with Ad-*PDK4* or Ad-Con for 48 h, followed by treatment with NAC (10 mM, 1 h) and then Ang II (1 μM, 48 h). Representative Western blot and quantification of NLRP3, Cleaved Caspase1, Cleaved IL-1β, Cleaved IL-18, GSDMD, and N-GSDMD protein levels (n=6

biological replicates). **B** Representative Western blot and quantification of NLRP3, Cleaved Caspase1, Cleaved IL-1β, Cleaved IL-18, GSDMD and N-GSDMD in MASMCs isolated from *Pdk4*^{SMKO} and *Pdk4*$^{fl/fl}$ mice and treated with NAC (10 mM, 1 h) followed by Ang II (1 μM, 48 h) (n=6 biological replicates). **C-D** Immunofluorescence staining of GSDMD, NLRP3, and ACTA2 was performed in suprarenal abdominal aortas from AAV-NC and AAV-*PDK4* mice treated with AAV-*PCSK9*^{DY}/Ang II for 28 days (n=8 biological replicates, representative images from eight biologically independent samples are shown). **E** Immunofluorescence staining for GSDMD and ACTA2 was performed in MASMCs transfected with Ad-Con or Ad-*PDK4* for 48 h, pre-treated with either NAC (10 mM) or MCC950 (10 μM) for 1 h, and subsequently stimulated with Ang II (1 μM) for 48 h (n=6 biological replicates). Data are presented as mean ± SD. (A-B, E) one-way ANOVA followed by Tukey's multiple-comparisons test. (C-D) Two-sided Unpaired Student's *t*-test. NS, nonsignificant. Source data are provided as a Source Data file.

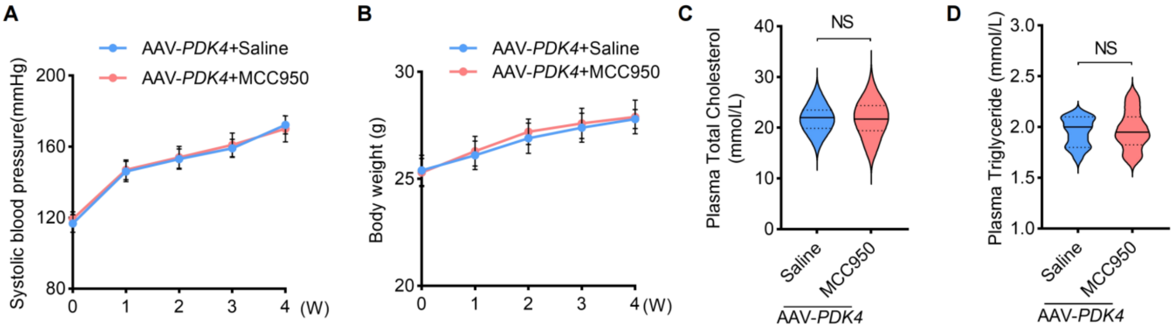

Fig. S8 | MCC950 has limited effects on body weight, systolic blood pressure, and serum lipids. **A-D** Eight-week-old male C57BL/6J mice were injected with AAV-*PCSK9*^{DY} and fed a Western diet. Two weeks later, mice were treated with AAV-*PDK4* and MCC950 or saline. Two weeks later, mice were infused with Ang II (1,500 ng/kg/min) for an additional 4 weeks. **A-D** Systolic blood pressure, body weight, serum TC, and serum TG levels in mice injected with or treated with AAV-*PDK4* + saline or AAV-*PDK4* + MCC950 (n=13 for AAV-*PDK4* + saline and n=20 for AAV-*PDK4* + MCC950, mice that died from aortic rupture were excluded from these measurements). Data are presented as mean ± SD. (A-D) Two-sided Unpaired Student's *t*-test. NS, nonsignificant. Source data are provided as a Source Data file.

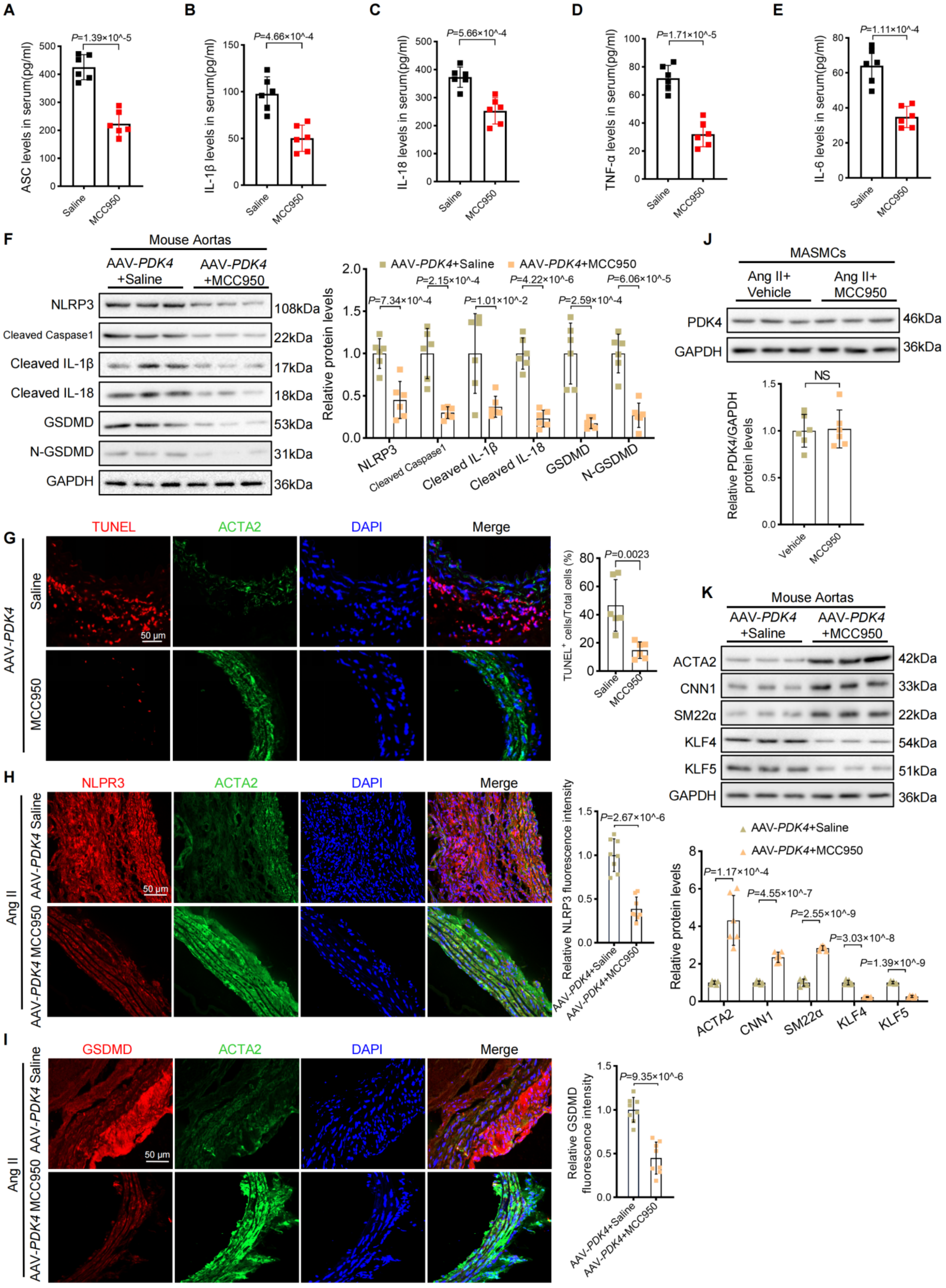
A
B
C
D
E
ASC levels in serum(pg/ml)
IL-1β levels in serum(pg/ml)
IL-18 levels in serum(pg/ml)
TNF-α levels in serum(pg/ml)
IL-6 levels in serum(pg/ml)
P=1.39×10^-5
P=4.66×10^-4
P=5.66×10^-4
P=1.71×10^-5
P=1.11×10^-4
Saline
MCC950
F
Mouse Aortas
AAV-PDK4 +Saline
AAV-PDK4 +MCC950
NLRP3
Cleaved Caspase1
Cleaved IL-1β
Cleaved IL-18
GSDMD
N-GSDMD
GAPDH
108kDa
22kDa
17kDa
18kDa
53kDa
31kDa
36kDa
Relative protein levels
AAV-PDK4+Saline
AAV-PDK4+MCC950
P=7.34×10^-4
P=2.15×10^-4
P=1.01×10^-2
P=4.22×10^-6
P=2.59×10^-4
P=6.06×10^-5
J
MASMCs
Ang II+ Vehicle
Ang II+ MCC950
PDK4
46kDa
36kDa
NS
Relative PDK4/GAPDH protein levels
Vehicle
G
TUNEL
ACTA2
DAPI
Merge
AAV-PDK4
50 μm
P=0.0023
TUNEL+ cells/Total cells (%)
K
ACTA2
CNN1
SM22α
KLF4
KLF5
42kDa
33kDa
22kDa
54kDa
51kDa
H
NLPR3
Ang II
AAV-PDK4 Saline
AAV-PDK4 MCC950
P=2.67×10^-6
Relative NLRP3 fluorescence intensity
P=1.17×10^-4
P=4.55×10^-7
P=2.55×10^-9
P=3.03×10^-8
P=1.39×10^-9
I
GSDMD
P=9.35×10^-6
Relative GSDMD fluorescence intensity

Fig. S9 | MCC950 alleviates AAA formation by attenuating PDK4-induced NLRP3 inflammasome activation and pyroptotic cell death. A-I Eight-week-old male C57BL/6J mice were injected with AAV-*PCSK9*^{DY} and fed a Western diet. Two weeks later, mice were treated with AAV-PDK4 and MCC950 or saline. After an additional 2 weeks, mice were infused with Ang II (1,500 ng/kg/min) for 4 weeks (n = 22 for MCC950 and n = 22 for saline). **A**-**E** Enzyme-linked immunosorbent assay (ELISA) was utilized to measure the serum levels of ASC, IL-1β, IL-18, TNF-α and IL-6 in AAV-*PDK4* + saline and AAV-*PDK4* + MCC950 mice treated with AAV-*PCSK9*^{DY}/Ang II (n=6 biological replicates). **F** Representative Western blot and quantification of NLRP3, Cleaved Caspase1, Cleaved IL-1β, Cleaved IL-18, GSDMD and N-GSDMD protein levels in the abdominal aortas from AAV-*PDK4* + saline and AAV-*PDK4* + MCC950 mice treated with AAV-*PCSK9*^{DY}/Ang II (n=6 biological replicates). **G** Representative images of TUNEL staining in suprarenal abdominal aortas from AAV-*PDK4* + saline and AAV-*PDK4* + MCC950 mice treated with AAV-*PCSK9*^{DY}/Ang II (n=6 biological replicates). **H**-**I** Immunofluorescence staining of GSDMD, NLRP3, and ACTA2 in suprarenal abdominal aortas from AAV-*PDK4* + saline and AAV-*PDK4* + MCC950 mice treated with AAV-*PCSK9*^{DY}/Ang II (n=8 biological replicates). **J** Representative Western blot and quantification of PDK4 protein levels in MASMCs pre-treated with MCC950 (10 μM) for 1 h, followed by stimulation with Ang II (1 μM) for 48 h (n=6 biological replicates). **K** Representative Western blot and quantification of ACTA2, CNN1, SM22α, KLF4, and KLF5 protein levels in abdominal aortas from AAV-*PDK4* + saline- and AAV-*PDK4* + MCC950-treated mice after AAV-*PCSK9*^{DY}/Ang II treatment (n=6 biological replicates). Data are presented as mean ± SD. (A-K) Two-sided Unpaired Student's *t*-test. NS, nonsignificant. Source data are provided as a Source Data file.

Supplementary Tables

Supplementary Table 1. SNP genotyping analysis used in this study.

dbSNP ID	C57BL/6J Alleles	*Pdk4*^{SMKO} (n=5 biological replicates)	*Pdk4*$^{fl/fl}$ (n=5 biological replicates)	Genetic Background
rs13475886	T/T	T/T	T/T	C57BL/6J
rs13476337	A/A	A/A	A/A	C57BL/6J
rs13476359	G/G	G/G	G/G	C57BL/6J
rs33142586	A/A	A/A	A/A	C57BL/6J
rs13476956	C/C	C/C	C/C	C57BL/6J
rs13477019	T/T	T/T	T/T	C57BL/6J
rs13477132	G/G	G/G	G/G	C57BL/6J
rs13477622	T/T	T/T	T/T	C57BL/6J
rs13477746	T/T	T/T	T/T	C57BL/6J
rs13478320	C/C	C/C	C/C	C57BL/6J
rs3662161	A/A	A/A	A/A	C57BL/6J
rs33208334	T/T	T/T	T/T	C57BL/6J
rs30764547	T/T	T/T	T/T	C57BL/6J
rs13478783	A/A	A/A	A/A	C57BL/6J
rs6157367	T/T	T/T	T/T	C57BL/6J
rs13479522	A/A	A/A	A/A	C57BL/6J
rs13479540	A/A	A/A	A/A	C57BL/6J
rs3709624	T/T	T/T	T/T	C57BL/6J
rs13480100	T/T	T/T	T/T	C57BL/6J
rs13480109	A/A	A/A	A/A	C57BL/6J
rs13480122	T/T	T/T	T/T	C57BL/6J
rs13480575	T/T	T/T	T/T	C57BL/6J
rs13480619	T/T	T/T	T/T	C57BL/6J
rs29359333	G/G	G/G	G/G	C57BL/6J
rs13480628	T/T	T/T	T/T	C57BL/6J
rs13459122	T/T	T/T	T/T	C57BL/6J
rs13480759	C/C	C/C	C/C	C57BL/6J
rs3659787	G/G	G/G	G/G	C57BL/6J
rs13481014	T/T	T/T	T/T	C57BL/6J
rs13481117	G/G	G/G	G/G	C57BL/6J
rs13481403	G/G	G/G	G/G	C57BL/6J
rs13481439	A/A	A/A	A/A	C57BL/6J
rs223935946	T/T	T/T	T/T	C57BL/6J
rs13481569	G/G	G/G	G/G	C57BL/6J
rs13481634	A/A	A/A	A/A	C57BL/6J
rs13481676	A/A	A/A	A/A	C57BL/6J

dbSNP, Database of Single Nucleotide Polymorphisms.

Supplementary Table 2. Characteristics of patients included in this study.

Characteristics	AAA (n = 8)
Age (years)	63.5 ± 4.504
Male (n, %)	7 (87.5)
Body mass index (kg/m^2)	25.03 ± 1.061
History of smoking (n, %)	3 (37.5)
Hypertension (n, %)	6 (75.0)
Diabetes mellitus (n, %)	1 (12.5)
Hyperlipidemia (n, %)	3 (37.5)
Maximum aortic diameter (cm)	6.3 ± 1.697

Data are presented as number (percentage) or mean ± standard deviation. AAA, abdominal aortic aneurysm.

Supplementary Table 3. Primers used in this study.

Gene	Forward primers (5′ – 3′)	Reverse primers (5′ – 3′)
Primers used for genotyping		
Pdk4$^{fl/fl}$ F1	CTCCTGGGTCATTAGTGATTGCAT	AAAGATGATTCTAGCAAGCCACTG
Pdk4$^{fl/fl}$ F2	AGATGGAGCAGCTCAAGTTCTTC	GTGGATTCGGACCAGTCTGA
Myh11-cre	TGACCCCATCTCTTCACTCC	AGTCCCTCACATCCTCAGGTT
Primers used for RT-PCR		
PDK4 (mouse)	AGGGAGGTCGAGCTGTTCTC	GGAGTGTTCACTAAGCGGTCA
PDK4 (human)	TTTCCAGACCAACCAATTCACA	TGCCCGCATTGCATTCTTA
ACTA2 (mouse)	GGCATCCACGAAACCACCTA	TTCCTGACCACTAGAGGGGG
CNN1 (mouse)	GGCAGATCTTTGAGCCAGGT	GGCCCCAAGACTCCAATGAT
SM22α (mouse)	TCCCCAAATATGGAGCCTGT	CACAGCCAAACTGCCCAAAG
KLF4 (mouse)	GTGCCCCGACTAACCGTTG	GAGAGGGGACTTGTGACTGC
KLF5 (mouse)	CAGGTACGCGCTCTCTTAGG	GGGCATGTCTAGATCCGGTG
Tfam (mouse)	CCGTATTGCGTGAGACGAAC	TCGTTTCACACTTCGACGGA
Tfb1m (mouse)	CCCACCATTCGGGAGATCAT	TAGAACCCGCAGCTTTCTGG
Ogdh (mouse)	TGAGAGTTTGGAGCCCGGA	GCAAGATCAAGTTTGTCTGTGGA

Pgc1α (mouse)	ATTTCAGCTGCCTTATTGG TTTCG	AGCAGCACACTGGTTGGAA G
Hif1α (mouse)	CTTGACAAGCTAGCCGGAG G	AATATGGCCCGTGCAGTGA A
Slc2a1 (mouse)	AGGGCCTAAGGTCACATG AAG	AAACAGCGACACCACAGTG A
Myc (mouse)	GCTTGGCGGGAAAAAGAA GG	CGACCGCAACATAGGATGG A
Pgk1 (mouse)	GATGCTTTCCGAGCCTCAC T	TTGGCCAGTCTTGGCATTCT
IL-1β (mouse)	TGCCACCTTTTGACAGTGA TG	TTCTTGTGACCCTGAGCGA C
IL-18 (mouse)	CCTTTGAGGCATCCAGGAC A	GTCTGGTCTGGGGTTCACT G
TNF-α (mouse)	ACCCTCACACTCACAAACC A	ACCCTGAGCCATAATCCCC T
IL-6 (mouse)	GCCTTCTTGGGACTGATGC T	TGTGACTCCAGCTTATCTCT TGG
β-actin (mouse)	GTTGGAGCAAACATCCCCC A	CGCGACCATCCTCCTCTTA G
mtDNA ATP-6	ACCAATAGCCCTGGCCGTA C	GGTGGCGCTTCCAATTAGG T
nDNA GAPDH	CGGGGCTCTCCAGAACATC	ATGACCTTGCCCACAGCCT

Supplementary Table 4. Design of the animal experiments.

Mouse group	substrain	Sex	Age (week)	Number (prior to experiment)	Number (after termination)	Littermates (Yes/No)
wild-type + saline	C57BL/6J	male	8	6	6	No
wild-type + *PCSK9*^{DY}/Ang II	C57BL/6J	male	8	6	5	No
PDK4 Wild-type (*Pdk4*$^{fl/fl}$) + *PCSK9*^{DY}/Ang II	C57BL/6J	male	8	22	18	No
PDK4 VSMC-knockout (*Pdk4*^{SMKO}) + *PCSK9*^{DY}/Ang II	C57BL/6J	male	8	22	22	No
wild-type + AAV-NC+ *PCSK9*^{DY}/Ang II	C57BL/6J	male	8	21	18	No
wild-type + AAV-*PDK4* + *PCSK9*^{DY}/Ang II	C57BL/6J	male	8	21	12	No
PDK4 Wild-type (*Pdk4*$^{fl/fl}$) + *PCSK9*^{DY}/Ang II 3d	C57BL/6J	male	8	16	16	No
PDK4 VSMC-knockout (*Pdk4*^{SMKO}) + *PCSK9*^{DY}/Ang II 3d	C57BL/6J	male	8	16	16	No
wild-type + AAV-*PCSK9*^{DY}/Ang II + saline	C57BL/6J	male	8	22	16	No
wild-type + AAV- +*PCSK9*^{DY}/Ang II +DCA	C57BL/6J	male	8	22	21	No
wild-type + AAV-*PDK4* + *PCSK9*^{DY}/Ang II + saline	C57BL/6J	male	8	22	13	No
wild-type + AAV-*PDK4* + *PCSK9*^{DY}/Ang II + MCC950	C57BL/6J	male	8	22	20	No

Supplementary Table 5. Gene sets used in this study.

Pathway-related gene sets					
Pyroptosis					
Gsdmd	*Adora2a*	*Adora3*	*Smim1*	*Cd274*	*Icam1*
Casp1	*Prkaca*	*Osm*	*Tet2*	*Setd7*	*Stk4*
Casp4	*Tirap*	*Pecam1*	*Ctsl*	*Fgf21*	*Gsk3b*
Gsdmc4	*Trim24*	*Mettl14*	*Mlkl*	*Ikzf1*	*Ptgs2*
Nlrp1b	*Naip2*	*Bak1*	*Ptpn11*	*Bsg*	*Mst1*
Il1b	*Mefv*	*Bax*	*Mapk14*	*Cebpb*	*Prf1*
Gsdma	*Dhx9*	*Scaf11*	*Apoe*	*Tfam*	*Prmt5*
Aim2	*Stat3*	*Gja1*	*Anxa1*	*Trim21*	*Elavl1*
Casp8	*Sirt1*	*Snip1*	*Sdhb*	*Nlrx1*	*Mpeg1*
Gzma	*Ctsg*	*Prdm1*	*Chmp2a*	*Usf2*	*Abl1*
Pycard	*Nek7*	*Vdr*	*Chmp2b*	*Slc30a7*	*Epha2*
Il18	*Trem2*	*Ager*	*Chmp3*	*Malt1*	*Hdac6*
Zbp1	*Foxo3*	*Ikbke*	*Chmp4b*	*Tlr2*	*Tlr3*
Casp3	*Trp53*	*Pkm*	*Chmp4c*	*Cycs*	*Sqstm1*
Nlrc4	*Nfe2l2*	*Ifi204*	*Chmp7*	*Il1a*	*Cdk9*
Casp6	*Txnip*	*Ifi203*	*Eef2k*	*Irf1*	*Irf3*
Zdhhc1	*Ddx3x*	*Mndal*	*Fpr2*	*Irf2*	*Ucp1*
Pten	*Sesn2*	*Ifi205*	*Htra1*	*Trp63*	*Trem1*
Adora1	*Elane*	*Crtac1*	*Pak2*	*Casp9*	*Tslp*
Adora2b	*Ubr2*	*Sez6l2*	*Ndufa13*	*Gpx4*	*Il6*
Nod2	*Plcg1*				

OXPHOS					
Cox17	*Cox5b*	*Cox6b1*	*Cox7a2l*	*Cox7b*	*mt-Atp6*
mt-Nd2	*mt-Nd4*	*Ndufa4l2*	*Ndufb3*	*Ndufb6*	*Ndufc1*
Ndufc2					
ATP.synthesis					
Lrpprc	*Etfdh*	*mt-Nd6*	*Ndufb10*	*Atp5f1d*	*Surf1*
Trap1	*Tmem186*	*Ndufa1*	*Ndufc1*	*Timmdc1*	*Ucp1*
Atp5pd	*Ndufaf3*	*Ndufa2*	*Ndufc2*	*Atp5f1e*	*Ucp2*
Atp5mg	*Uqcrq*	*Ndufa3*	*Ndufs1*	*Atp5pb*	*Ucp3*
Uqcr11	*Dmac2l*	*Ndufa4*	*Ndufs2*	*Atp5mc1*	*Uqcrb*
Cox20	*Cox18*	*Ndufa5*	*Ndufs3*	*Atp5mc2*	*Uqcrc1*
Ndufa11	*Acad9*	*Ndufa6*	*Ndufv1*	*Atp5mc3*	*Uqcrc2*
Cox4i1	*Ndufaf4*	*Ndufa7*	*Ndufs4*	*Atp5me*	*Uqcrfs1*
Cox5b	*Uqcr10*	*Ndufa8*	*Ndufs5*	*Atp5pf*	*Uqcrh*
Cox6a1	*Ndufs7*	*Ndufa9*	*Ndufs6*	*Atp5po*	*Ndufaf5*
Cox6b1	*mt-Atp6*	*Ndufa10*	*Ndufs8*	*Cycs*	*Coq10b*
Cox6c	*mt-Atp8*	*Ndufab1*	*Ndufv2*	*Ndufb11*	*Nubpl*
Cox7b	*mt-Co1*	*Ndufb1*	*Ndufv3*	*Ndufaf7*	*Cox14*
Cox7c	*mt-Co2*	*Ndufb2*	*Atp5f1a*	*Coa1*	*Slc25a14*
Cox8a	*mt-Co3*	*Ndufb3*	*Atp5f1b*	*Tmem126b*	*Cox19*
Cox11	*mt-Cyb*	*Ndufb4*	*Atp5f1c*	*Ndufa12*	*Cox7a2l*
Ndufaf6	*mt-Nd1*	*Ndufb5*	*Ndufa13*	*Sco1*	*Ndufaf2*
Pm20d1	*mt-Nd2*	*Ndufb6*	*Ndufaf1*	*Sdha*	*Coq10a*
Cyc1	*mt-Nd3*	*Ndufb7*	*Taco1*	*Sdhb*	*Cox5a*

Etfa	*mt-Nd4*	*Ndufb8*	*Cox16*	*Sdhc*	*Slc25a27*
Etfb	*mt-Nd5*	*Ndufb9*	*Ecsit*	*Sdhd*	*Atp5mf*
Sco2					
Complex.I					
Ndufaf6	*mt-Nd5*	*Ndufa10*	*Ndufb8*	*Ndufs4*	*Ndufb11*
Tmem186	*mt-Nd6*	*Ndufab1*	*Ndufb9*	*Ndufs5*	*Tmem70*
Ndufaf3	*Ndufa1*	*Ndufb1*	*Ndufb10*	*Ndufs6*	*Dmac2*
Acad9	*Ndufa2*	*Ndufb2*	*Ndufc1*	*Ndufv2*	*Ndufaf7*
Ndufaf4	*Ndufa3*	*Ndufb3*	*Ndufc2*	*Ndufv3*	*Foxred1*
mt-Nd1	*Ndufa5*	*Ndufb4*	*Ndufs1*	*Ndufa13*	*Coa1*
mt-Nd2	*Ndufa6*	*Ndufb5*	*Ndufs2*	*Ndufaf1*	*Tmem126b*
mt-Nd4	*Ndufa7*	*Ndufb6*	*Ndufs3*	*Ecsit*	*Ndufa12*
mt-Nd4l	*Ndufa8*	*Ndufb7*	*Ndufv1*	*Timmdc1*	*Nubpl*
Dmac1	*Ndufaf2*				
Complex.II					
Sdhaf4	*Sdhaf2*	*Sdhaf3*	*Sdha*	*Sdhb*	*Sdhc*
Sdhd	*Sdhaf1*				
Complex.III					
Uqcr11	*Cyc1*	*Uqcrq*	*Uqcr10*	*mt-Cyb*	*Ttc19*
Uqcc1	*Bcs1l*	*Uqcrb*	*Uqcrc1*	*Uqcrc2*	*Uqcrfs1*
Uqcrh	*Uqcc3*	*Uqcc2*	*Lyrm7*		
Complex.IV					
Pet100	*Cox6a1*	*Cox8a*	*Higd1a*	*mt-Co2*	*Tmem177*
Pet117	*Cox6b1*	*Cox10*	*Cox18*	*mt-Co3*	*Cox14*

Cox17	*Cox6c*	*Cox11*	*Coa3*	*Ndufa4*	*Cox19*
Cox20	*Cox7a2*	*Cox15*	*Coa6*	*Taco1*	*Cox5a*
Cox4i1	*Cox7b*	*Cmc1*	*Smim20*	*Cox16*	*Sco2*
Cox5b	*Cox7c*	*Pnkd*	*mt-Co1*	*Surf1*	
Glycolysis					
Abcb6	*Cyb5a*	*Hs2st1*	*Pkm*	*Acss1*	*Nup160*
Adora2b	*Dcn*	*Hs6st2*	*Pkp2*	*Acss2*	*Nup188*
Agl	*Ddit4*	*Hspa5*	*Plod1*	*Adh1*	*Nup205*
Agrn	*Depdc1a*	*Idh1*	*Plod2*	*Adh4*	*Nup210*
Ak3	*Dld*	*Idua*	*Pmm2*	*Adh5*	*Nup214*
Ak4	*Dpysl4*	*Ier3*	*Polr3k*	*Adh7*	*Nup35*
Akr1a1	*Dsc2*	*Igfbp3*	*Ppfia4*	*Aldh1a3*	*Nup37*
Aldh7a1	*Ecd*	*Il13ra1*	*Ppia*	*Aldh1b1*	*Nupl2*
Aldh9a1	*Efna3*	*Irs2*	*Ppp2cb*	*Aldh2*	*Nup43*
Aldoa	*Egfr*	*Isg20*	*Prps1*	*Aldh3a1*	*Nup50*
Aldob	*Egln3*	*Kdelr3*	*Psmc4*	*Aldh3a2*	*Nup54*
Alg1	*Elf3*	*Kif20a*	*Pygb*	*Aldh3b1*	*Nupl1*
Ang	*Eno1*	*Kif2a*	*Pygl*	*Aldh3b3*	*Nup62*
Angptl4	*Eno2*	*Lct*	*Qsox1*	*Aldoc*	*Nup85*
Ankzf1	*Ero1a*	*Ldha*	*Rars*	*Bpgm*	*Nup88*
Arpp19	*Ext1*	*Ldhc*	*Rbck1*	*Dlat*	*Nup93*
Artn	*Ext2*	*Lhpp*	*Rpe*	*Eno3*	*Nup98*
Aurka	*Fam162a*	*Lhx9*	*Rragd*	*Fbp1*	*Pfkfb3*
B3galt6	*Fbp2*	*Mdh1*	*Sap30*	*G6pc*	*Pfkfb4*

B3gat1	*Fkbp4*	*Mdh2*	*Sdc1*	*G6pc2*	*Pgm2l1*
B3gat3	*Fut8*	*Me1*	*Sdc2*	*Galm*	*Pgp*
B3gnt3	*G6pdx*	*Me2*	*Sdc3*	*Gapdh*	*Pom121*
B4galt1	*Gal3st1*	*Med24*	*Sdhc*	*Gck*	*Ppp2ca*
B4galt2	*Gale*	*Mertk*	*Slc16a3*	*Gpi1*	*Ppp2r1a*
B4galt4	*Galk1*	*Met*	*Slc25a10*	*Hk1*	*Ppp2r1b*
B4galt7	*Galk2*	*Mif*	*Slc25a13*	*Hk3*	*Ppp2r5d*
Bik	*Gapdhs*	*Miox*	*Slc35a3*	*Ldhal6b*	*Prkaca*
Bpnt1	*Gclc*	*Mpi*	*Slc37a4*	*Ldhb*	*Prkacb*
Cacna1h	*Gfpt1*	*Mxi1*	*Sod1*	*Pck1*	*Rae1*
Capn5	*Gfus*	*Nanp*	*Sox9*	*Pck2*	*Ranbp2*
Casp6	*Glce*	*Nasp*	*Spag4*	*Pdha1*	*Sec13*
Cd44	*Glrx*	*Ndst3*	*Srd5a3*	*Pdha2*	*Seh1l*
Cdk1	*Gmppa*	*Ndufv3*	*Stc1*	*Pdhb*	*Tpr*
Cenpa	*Gmppb*	*Nol3*	*Stc2*	*Pfkl*	*Slc2a1*
Chpf	*Gne*	*Nsdhl*	*Stmn1*	*Pfkm*	*Mpc1*
Chpf2	*Gnpda1*	*Nt5e*	*Taldo1*	*Pgk2*	*Mpc2*
Chst1	*Got1*	*P4ha1*	*Tff3*	*Pgm1*	*Slc2a2*
Chst12	*Got2*	*P4ha2*	*Tgfa*	*Pklr*	*Slc2a3*
Chst2	*Gpc1*	*Pam*	*Tgfbi*	*Bid*	*Slc2a4*
Chst4	*Gpc3*	*Paxip1*	*Tktl1*	*Cd4*	*Slc2a5*
Chst5	*Gpc4*	*Pcx*	*Tpbg*	*Pfkfb2*	*Prkaa1*
Cited2	*Gpr87*	*Pdk3*	*Tpi1*	*Aaas*	*Rb1*
Cldn3	*Gusb*	*Pfkfb1*	*Tpst1*	*Adpgk*	*Trp53*

Cldn9	*Gys1*	*Pfkp*	*Txn1*	*Gckr*	*Gpat3*
Cln6	*Gys2*	*Pgam1*	*Ugp2*	*Gnpda2*	*Gpd1*
Cog2	*Hax1*	*Pgam2*	*Vcan*	*Ndc1*	*Hif1a*
Col5a1	*Hdlbp*	*Pgk1*	*Vegfa*	*Nup107*	*Pparg*
Copb2	*Hk2*	*Pgls*	*Vldlr*	*Nup133*	
Cth	*Hmmr*	*Pgm2*	*Xylt2*	*Nup153*	
Cxcr4	*Homer1*	*Phka2*	*Zfp292*	*Nup155*	

Uncropped Western blot images

Supplementary Figure 1G

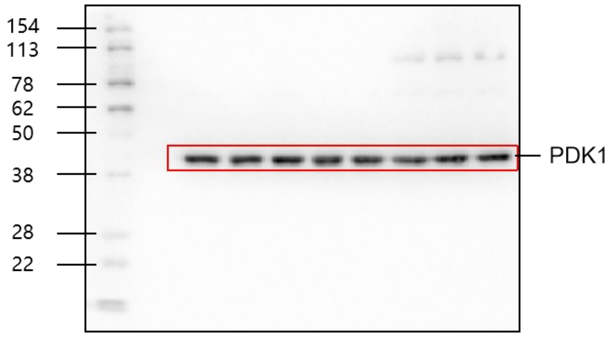

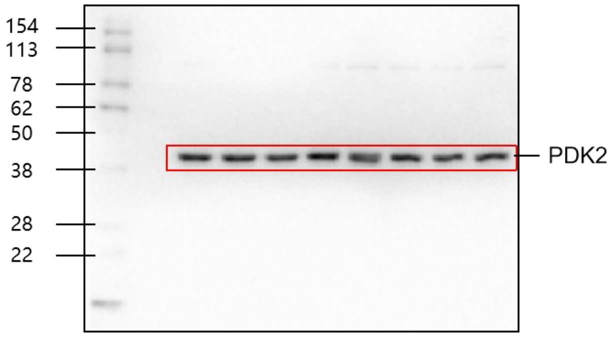

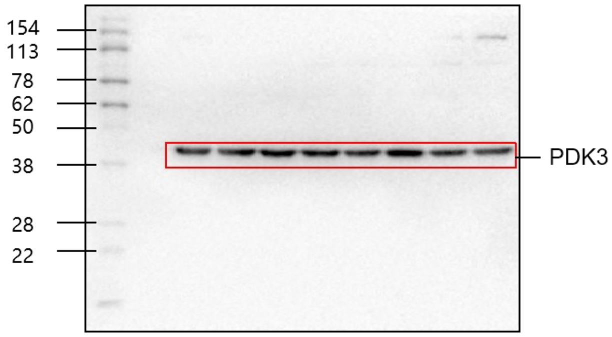

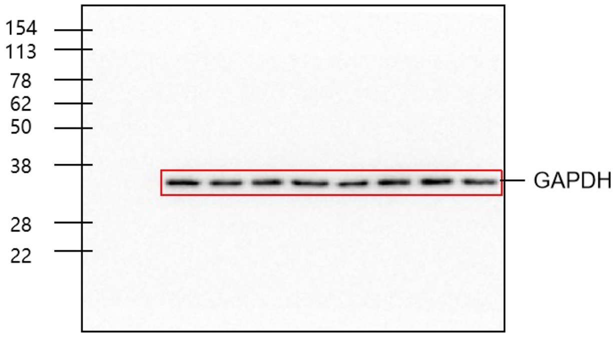

Supplementary Figure 1H

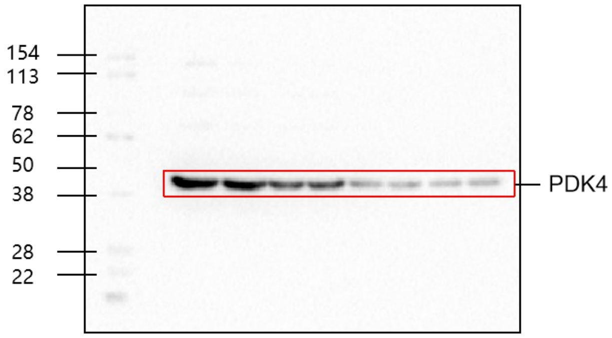

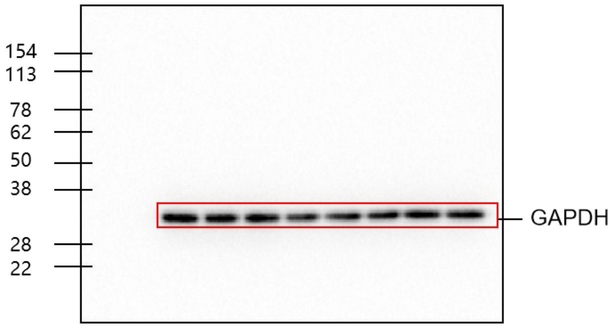

Supplementary Figure 2C

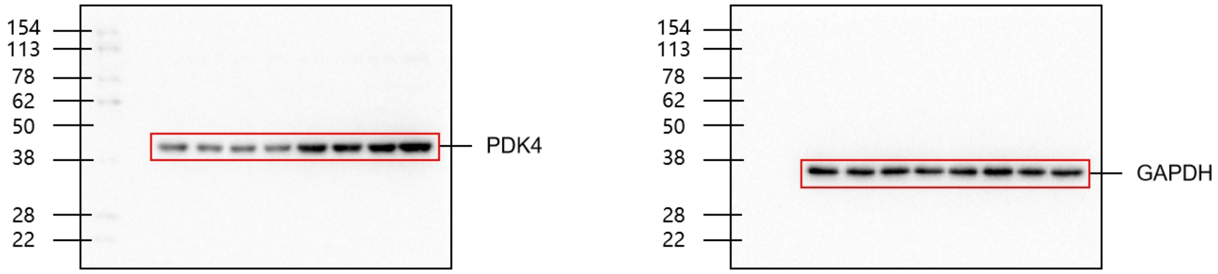

Supplementary Figure 2E

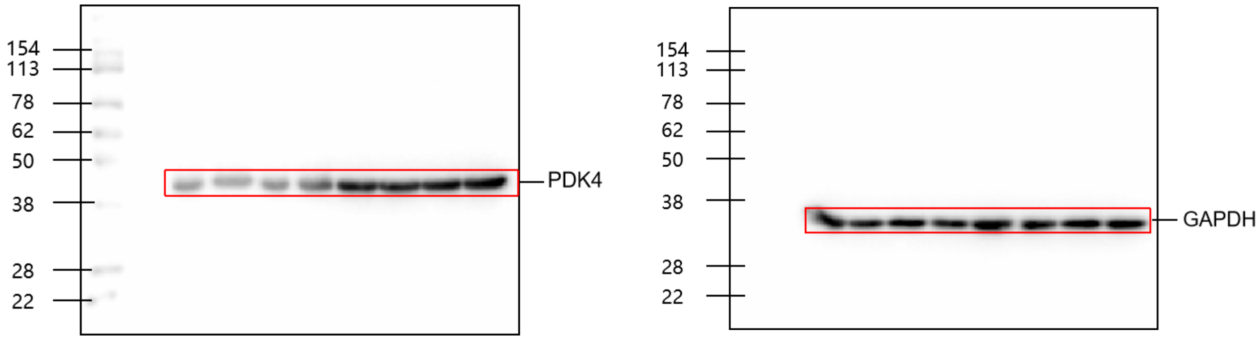

Supplementary Figure 2H

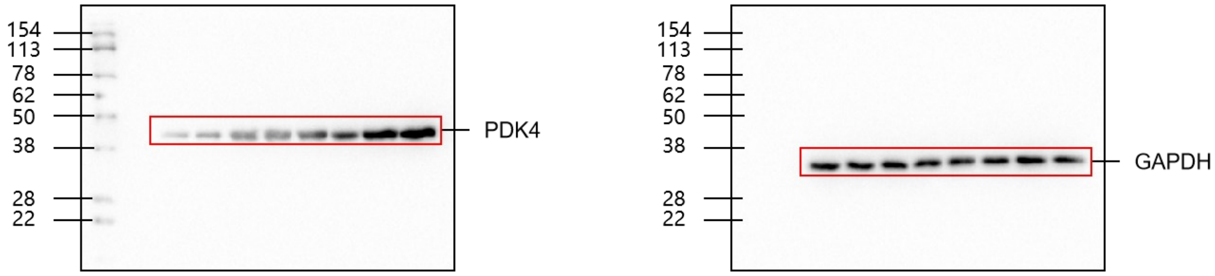

Supplementary Figure 3C

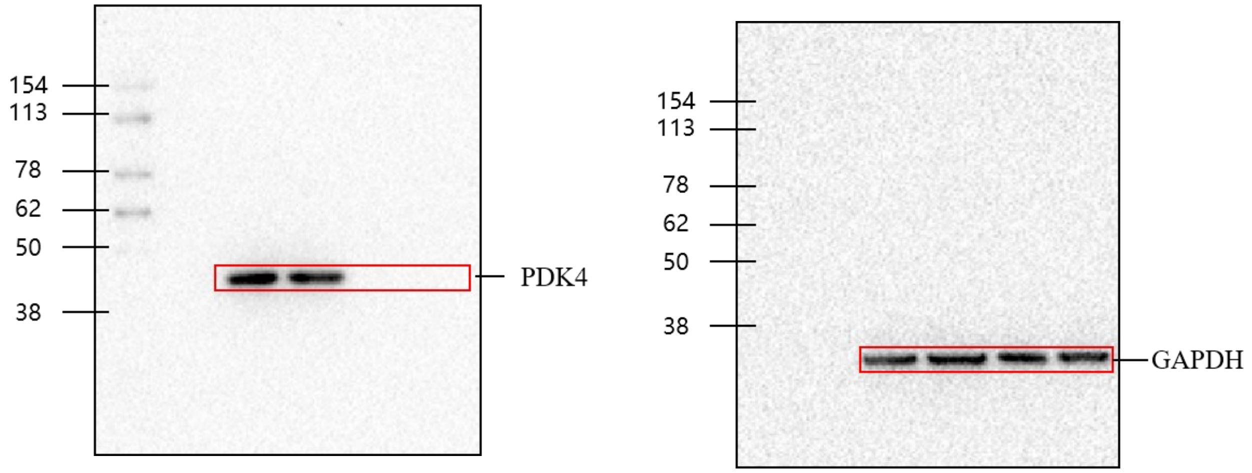

Supplementary Figure 4B

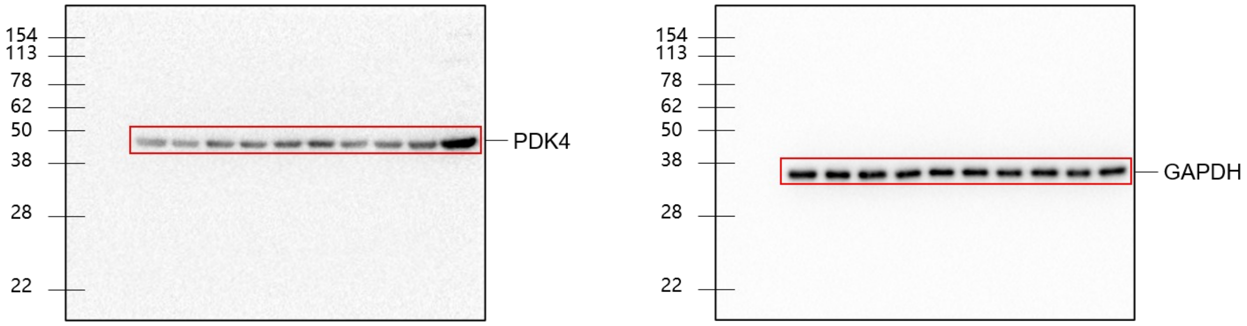

Supplementary Figure 4D

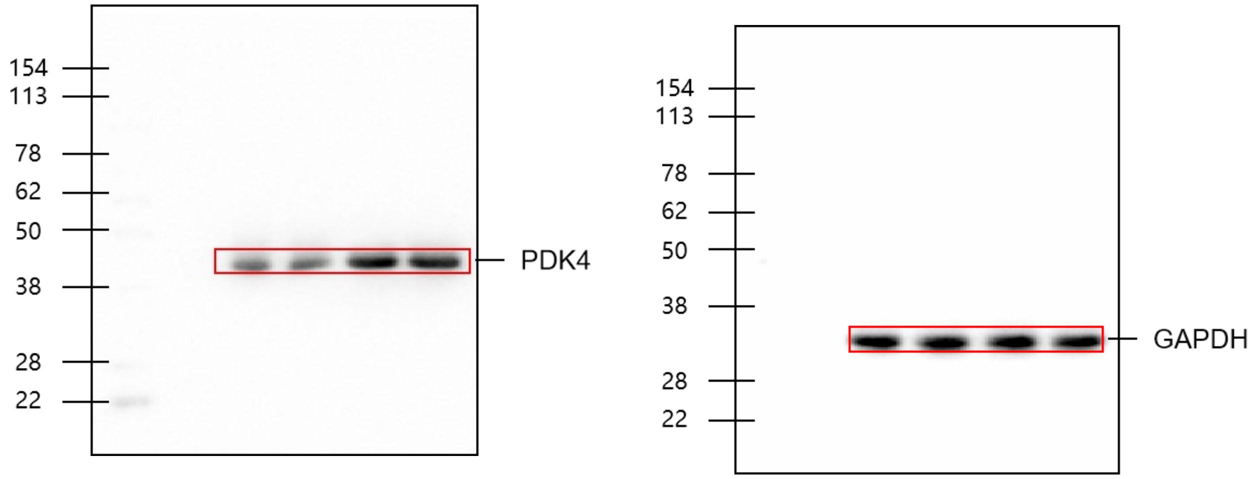

Supplementary Figure 5J

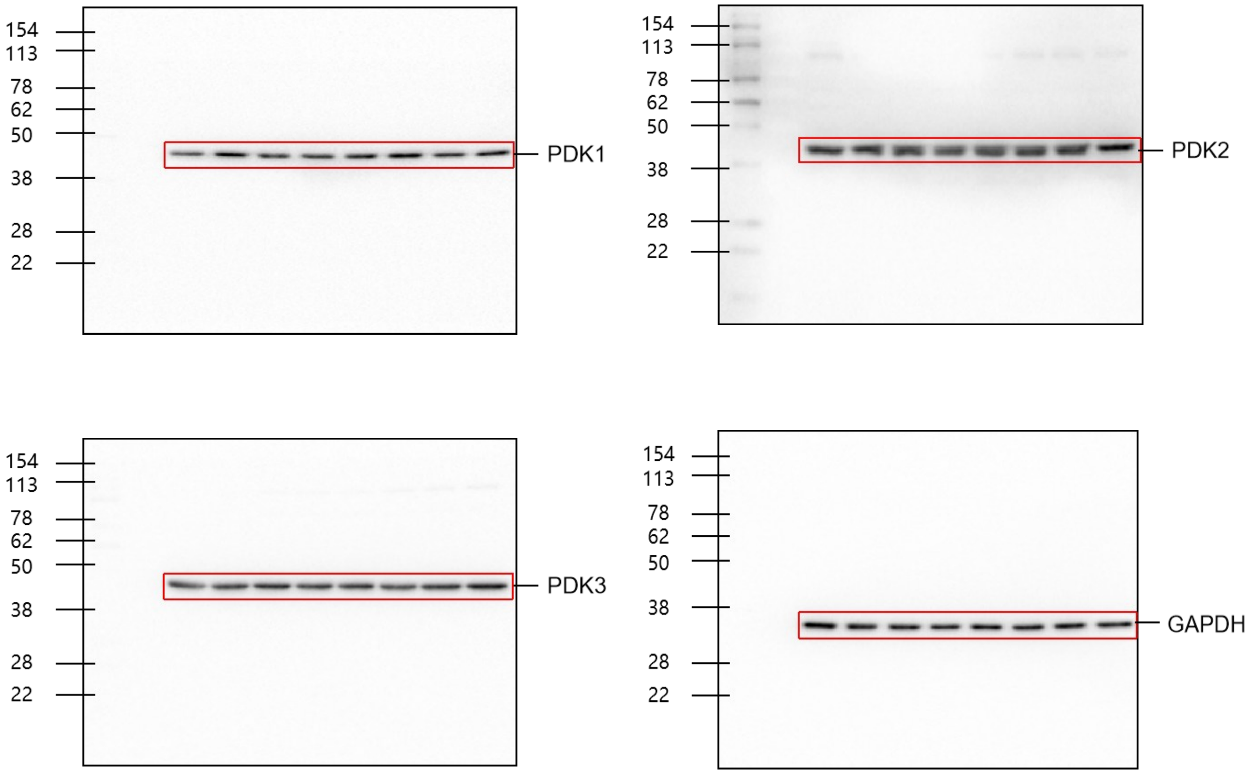

Supplementary Figure 6G

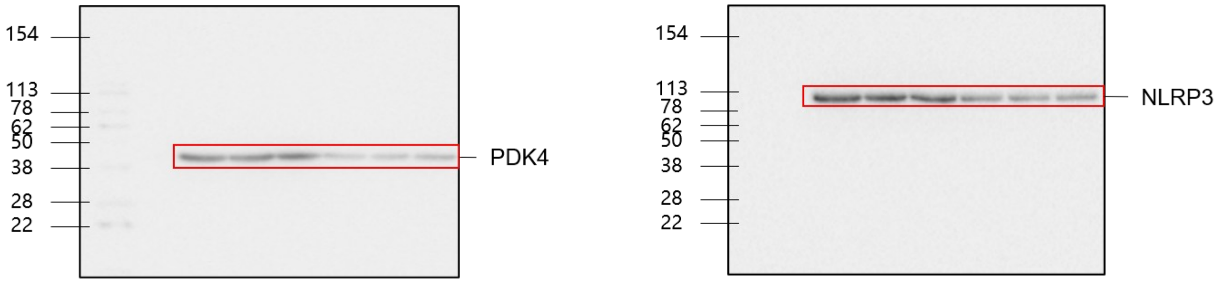

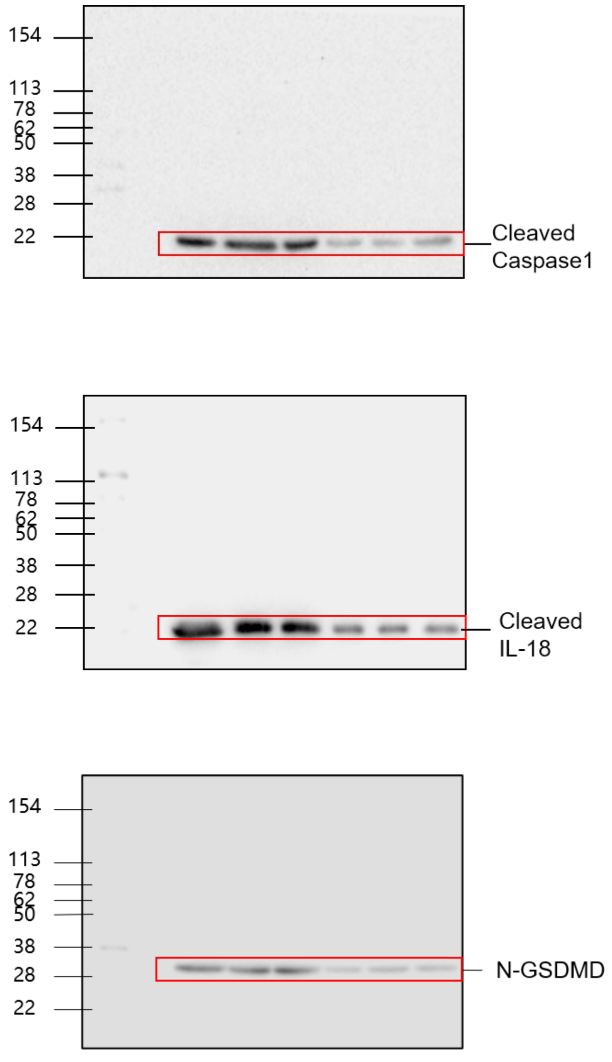

Supplementary Figure 6H

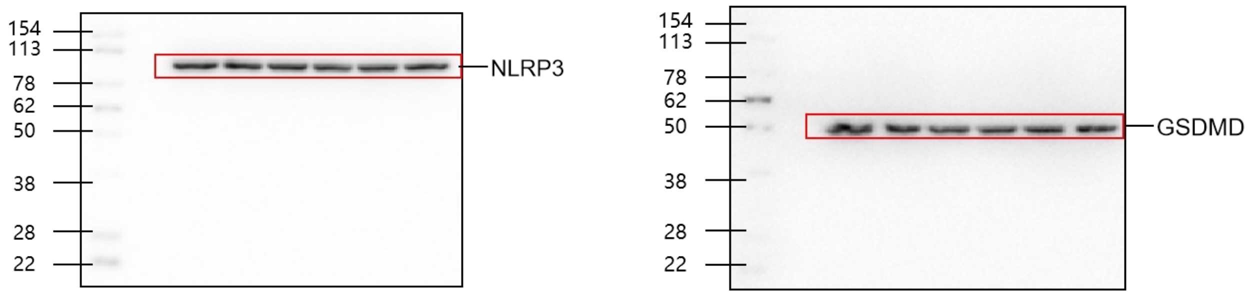

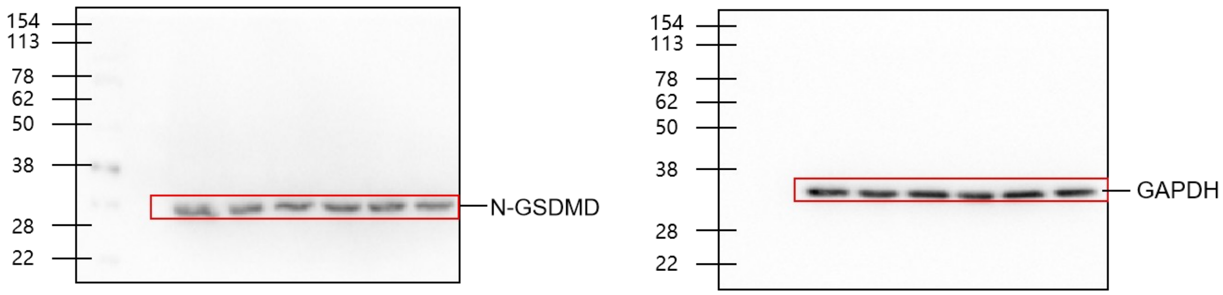

Supplementary Figure 6I

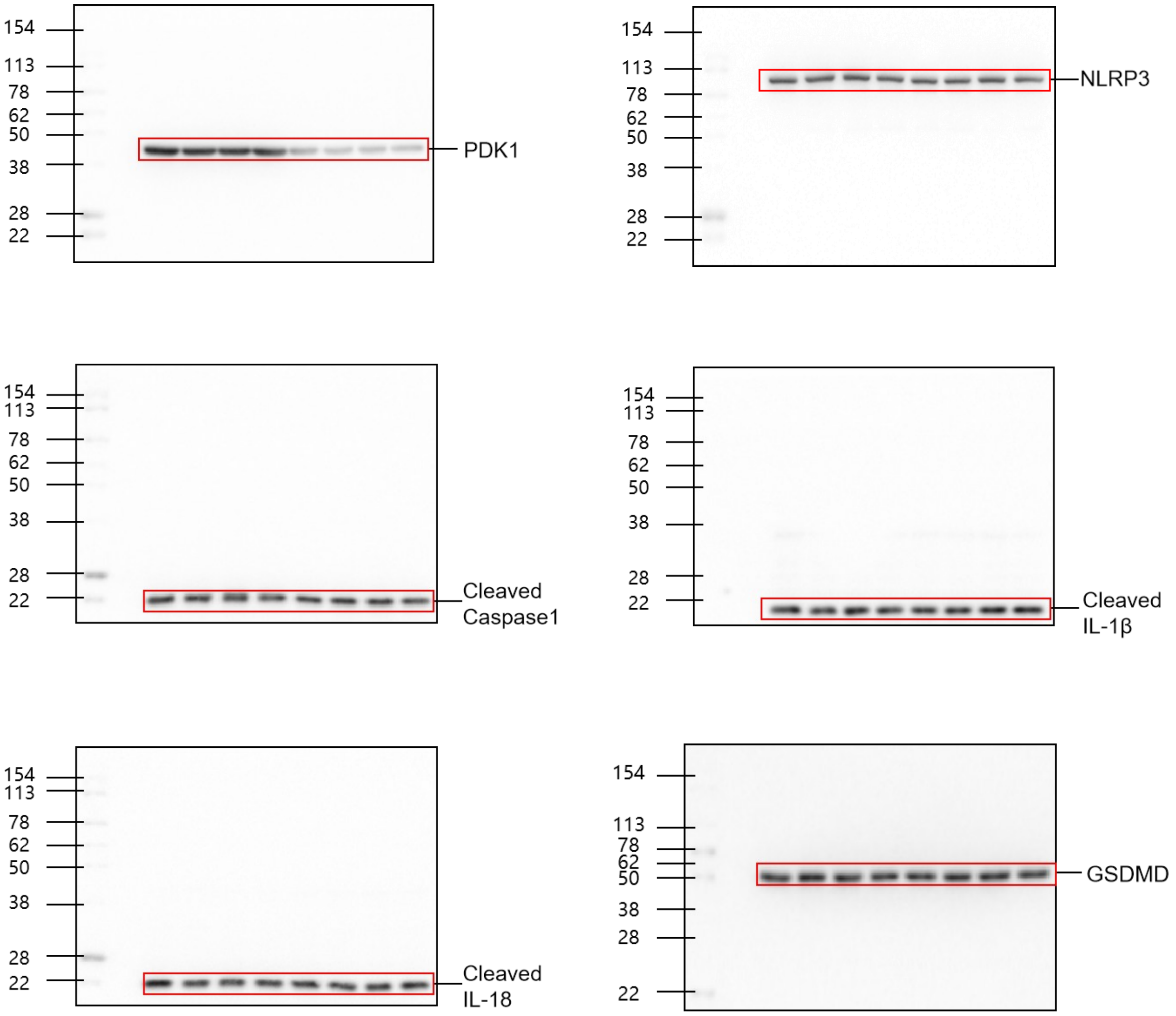

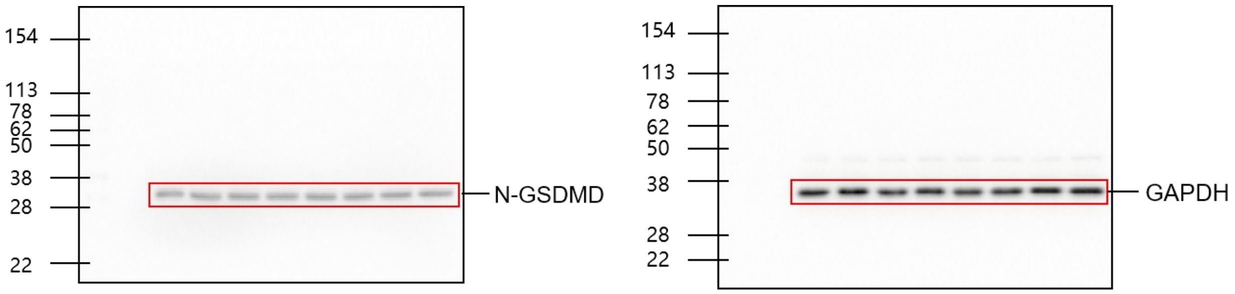

Supplementary Figure 7A

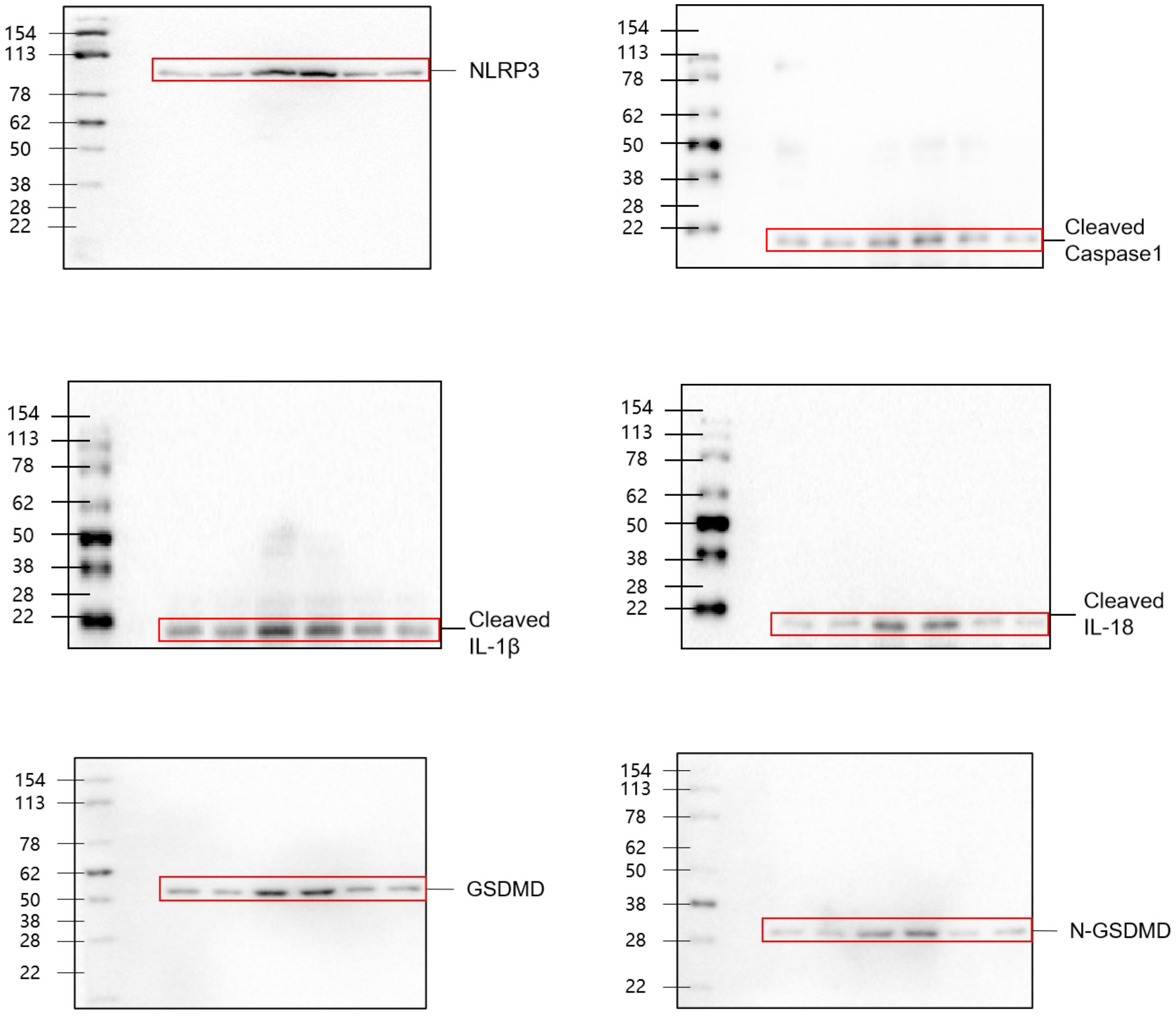

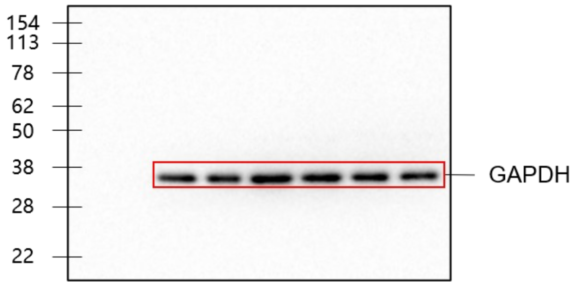

Supplementary Figure 7B

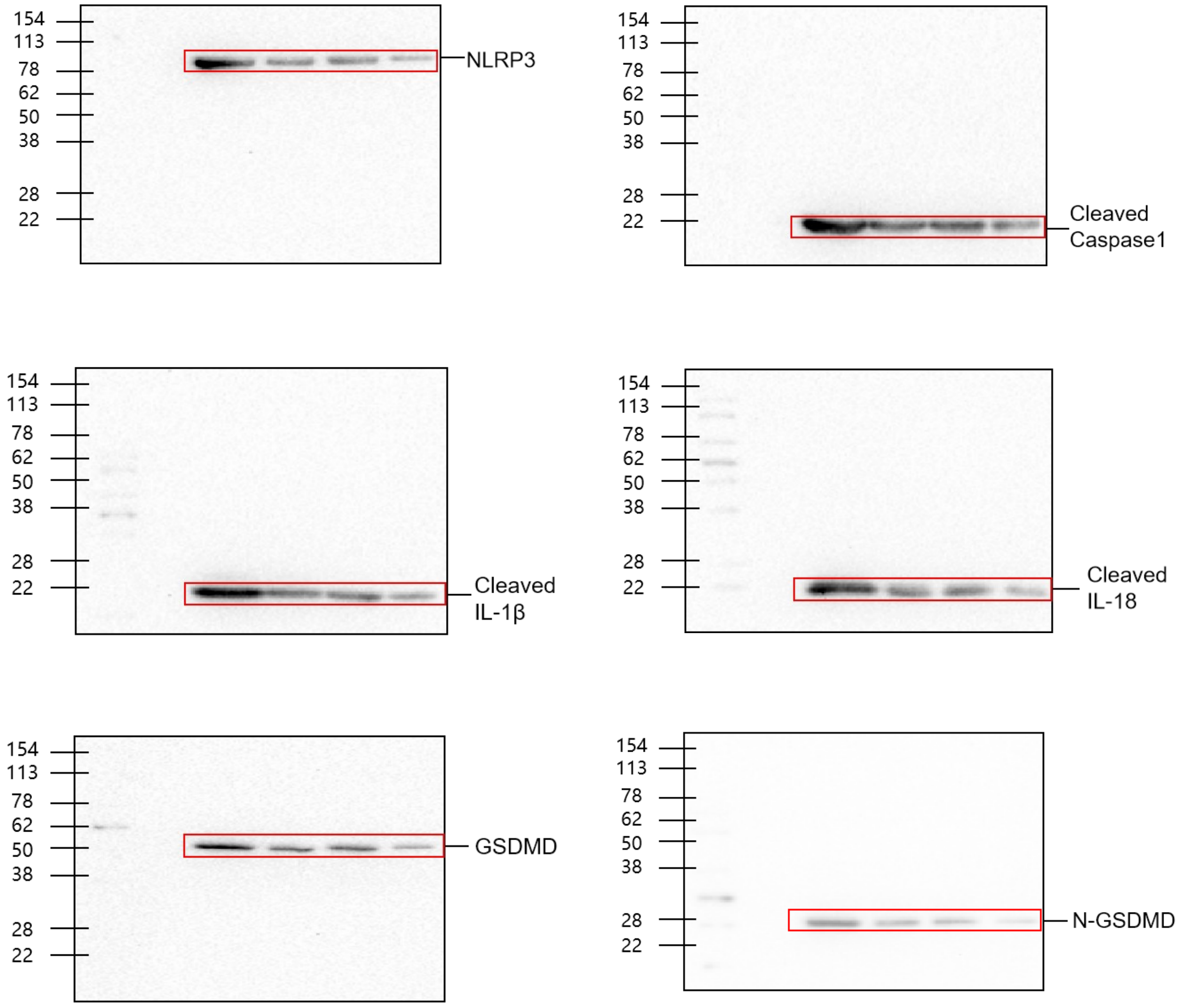

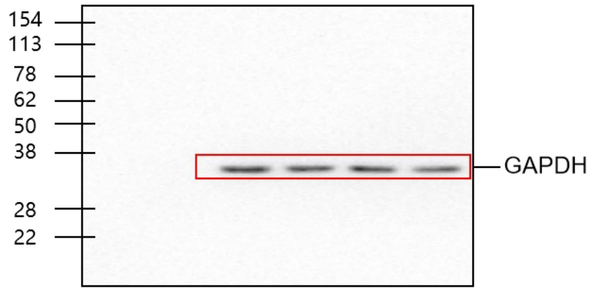

Supplementary Figure 9F

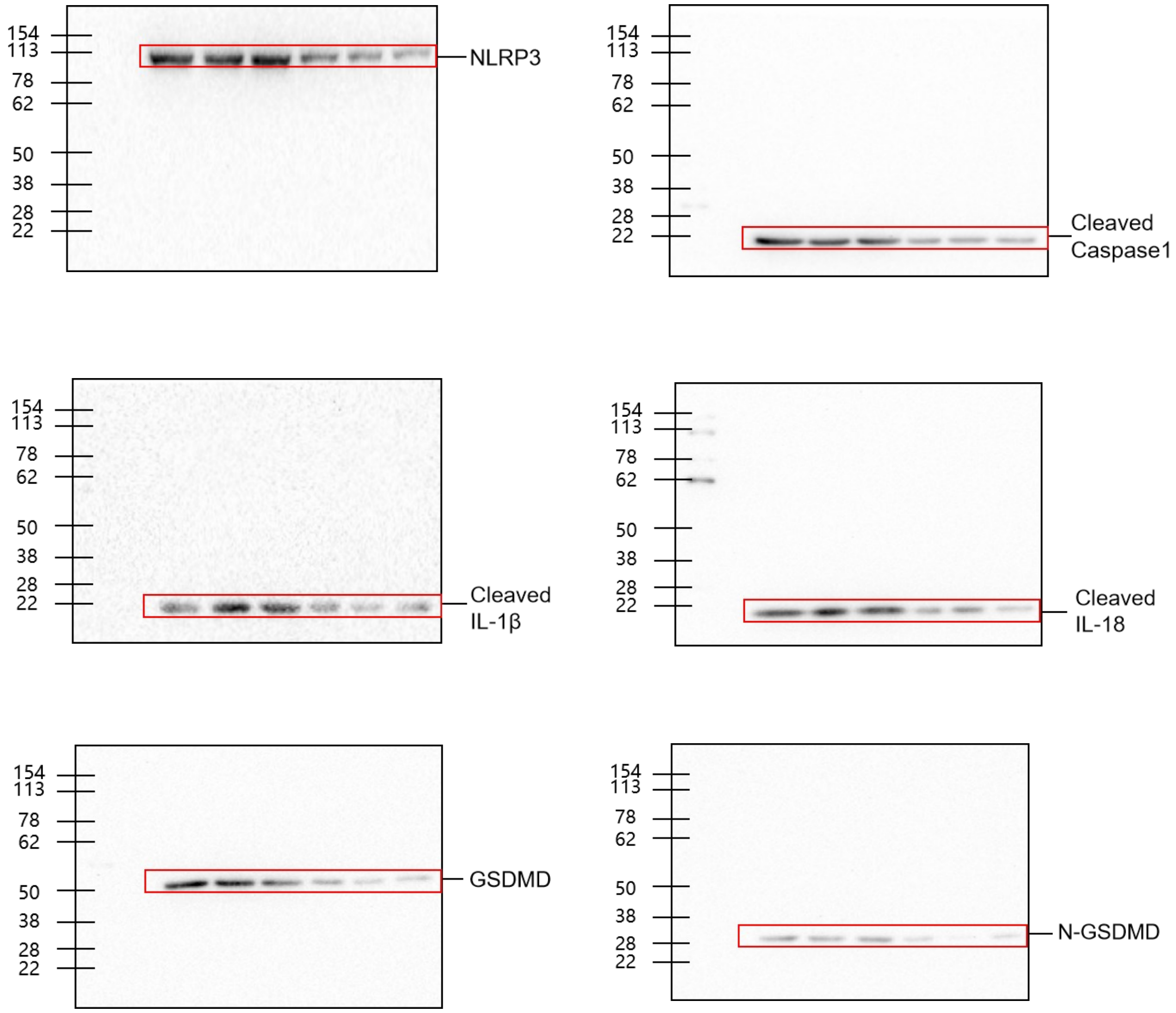

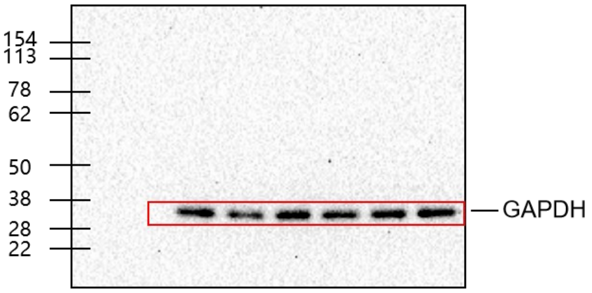

Supplementary Figure 9J

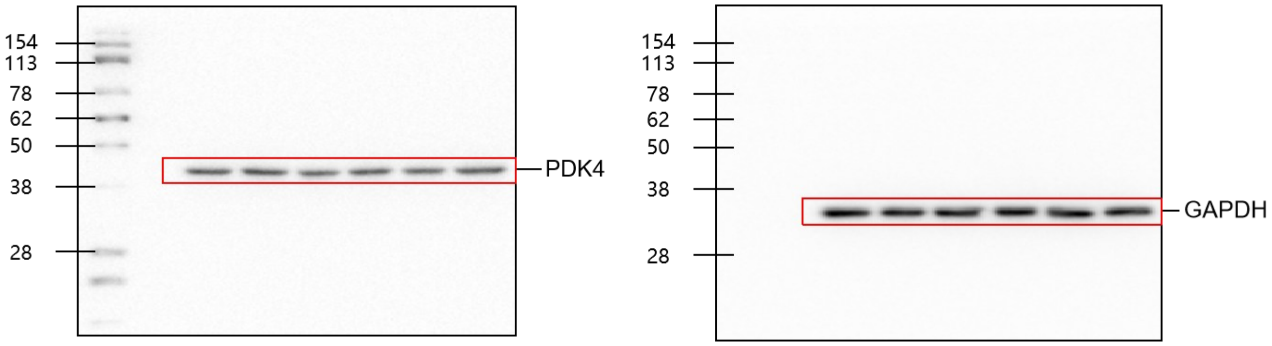

Supplementary Figure 9K

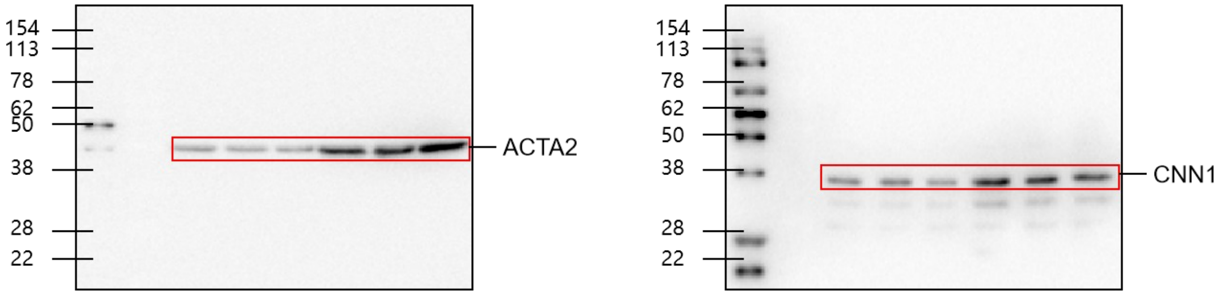

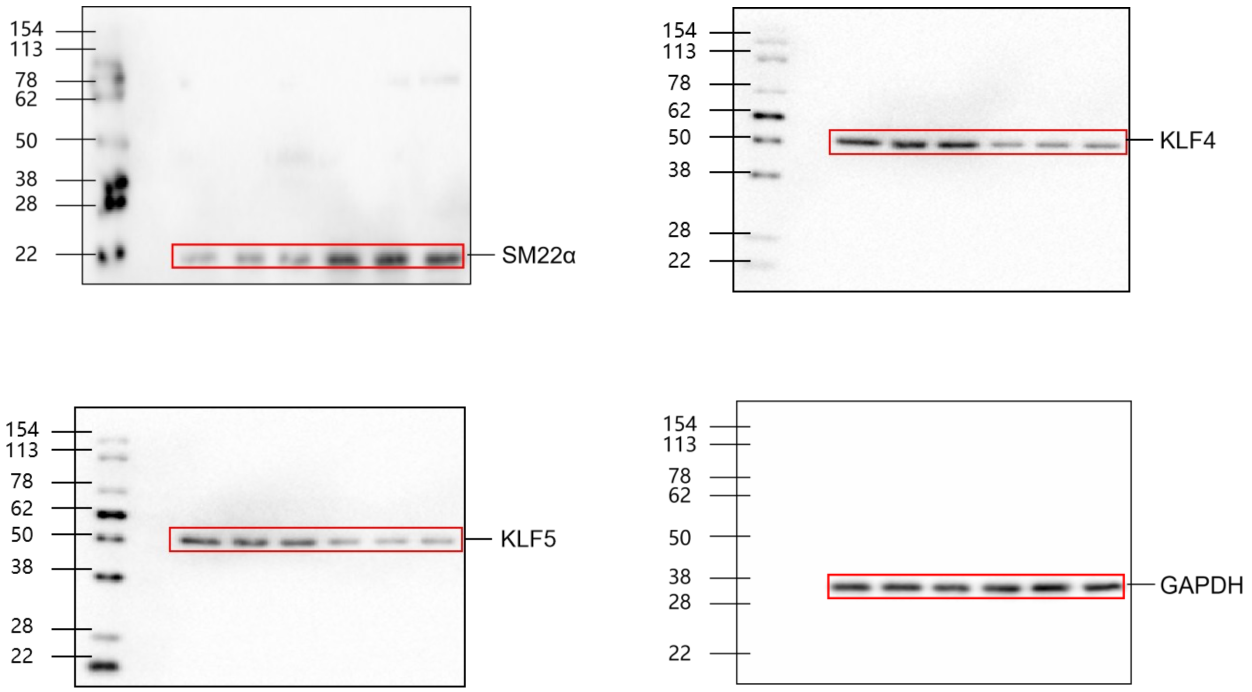
154
113
78
62
50
38
28
22
SM22α
KLF4
KLF5
GAPDH
