# Supplementary material for: PDK4 drives abdominal aortic aneurysm by promoting smooth muscle cell metabolic reprogramming and NLRP3-mediated pyroptosis
Source: Nat Commun. 2026 Apr 11;17:5086. doi: 10.1038/s41467-026-71610-w (PMC13246965; doi:10.1038/s41467-026-71610-w)
Supplement: Supplementary file 3 — Reporting Summary [file 41467_2026_71610_MOESM3_ESM.pdf]

nature portfolio

Corresponding author(s): Shaoyi Zheng, Jiaguo Zhou, Qinbao Peng, Xiu Liu

Last updated by author(s): Mar 18, 2026

Reporting Summary

Nature Portfolio wishes to improve the reproducibility of the work that we publish. This form provides structure for consistency and transparency in reporting. For further information on Nature Portfolio policies, see our Editorial Policies and the Editorial Policy Checklist.

Statistics

For all statistical analyses, confirm that the following items are present in the figure legend, table legend, main text, or Methods section.

n/a	Confirmed	
☐	☒	The exact sample size (*n*) for each experimental group/condition, given as a discrete number and unit of measurement
☐	☒	A statement on whether measurements were taken from distinct samples or whether the same sample was measured repeatedly
☐	☒	The statistical test(s) used AND whether they are one- or two-sided<br>*Only common tests should be described solely by name; describe more complex techniques in the Methods section.*
☒	☐	A description of all covariates tested
☐	☒	A description of any assumptions or corrections, such as tests of normality and adjustment for multiple comparisons
☐	☒	A full description of the statistical parameters including central tendency (e.g. means) or other basic estimates (e.g. regression coefficient) AND variation (e.g. standard deviation) or associated estimates of uncertainty (e.g. confidence intervals)
☐	☒	For null hypothesis testing, the test statistic (e.g. *F*, *t*, *r*) with confidence intervals, effect sizes, degrees of freedom and *P* value noted<br>*Give P values as exact values whenever suitable.*
☒	☐	For Bayesian analysis, information on the choice of priors and Markov chain Monte Carlo settings
☒	☐	For hierarchical and complex designs, identification of the appropriate level for tests and full reporting of outcomes
☒	☐	Estimates of effect sizes (e.g. Cohen's *d*, Pearson's *r*), indicating how they were calculated

Our web collection on statistics for biologists contains articles on many of the points above.

Software and code

Policy information about availability of computer code

Data collection	qPCR data were collected using a Light Cycler 480 real-time PCR instrument (Roche).<br>Western Blot images were acquired with a ChemiDoc XRS+ ultra-sensitive chemiluminescence gel imaging system (Bio-Rad).<br>Immunofluorescence images were captured on an LSM 980 fluorescence confocal microscope (Carl Zeiss).<br>Hematoxylin and Eosin and Verhoeff-van Gieson staining images were obtained using an Olympus BX63 digital camera microscope.<br>Ultrasonic imaging was performed with a Vevo 2100 ultrasound system.<br>ELISA assay results were measured by a Thermo Scientific microplate reader.<br>Oxygen consumption rate and Proton efflux rate measurements were acquired with Wave software (Agilent).<br>SNP genotyping sequencing was conducted on an ABI 3730xl sequencer.
Data analysis	Western Blot and immunofluorescence images were analyzed using ImageJ (version 1.8.0).<br>Single-cell RNA sequencing data were processed with R (version 4.4.1) and RStudio.<br>Statistical analyses were performed with GraphPad Prism (version 9.0).<br>Oxygen consumption rate and Proton efflux rate measurements were analyzed using Wave software (Agilent).<br>SNP genotyping data analysis was performed with SnapGene (version 4.2.4).

For manuscripts utilizing custom algorithms or software that are central to the research but not yet described in published literature, software must be made available to editors and reviewers. We strongly encourage code deposition in a community repository (e.g. GitHub). See the Nature Portfolio guidelines for submitting code & software for further information.

Data

Policy information about availability of data

All manuscripts must include a data availability statement. This statement should provide the following information, where applicable:

- Accession codes, unique identifiers, or web links for publicly available datasets
- A description of any restrictions on data availability
- For clinical datasets or third party data, please ensure that the statement adheres to our policy

The RNA sequencing data generated in this study have been deposited in the Gene Expression Omnibus (GEO) database under accession code GSE269546 [https://www.ncbi.nlm.nih.gov/geo/query/acc.cgi?acc=GSE269546] and GSE305105 [https://www.ncbi.nlm.nih.gov/geo/query/acc.cgi?acc=GSE305105]. Previously published datasets analyzed in this manuscript are also available from GEO under accession code GSE239620 [https://www.ncbi.nlm.nih.gov/geo/query/acc.cgi?acc=GSE239620]. All data supporting the findings are included in the main text, the supplementary materials, or the Source Data file. Source data are provided with this paper.

Research involving human participants, their data, or biological material

Policy information about studies with human participants or human data. See also policy information about sex, gender (identity/presentation), and sexual orientation and race, ethnicity and racism.

Reporting on sex and gender	Sex was considered in the study design through the inclusion of both male and female participants. All participants were human patients undergoing aortic procedures. The cohort included patients diagnosed with Abdominal Aortic Aneurysm (AAA) (abdominal aortic diameter ≥ 55 mm) and matched controls (adjacent non-aneurysmal aortic segments from the samepatients) (n=8). Data on sex were not disaggregated in this study. The collected AAA dataset includes both male and female participants, but analyses were not stratified by sex. Sex-disaggregated individual-level data for all human participants have been provided in the Source Data file. Patient characteristics are reported in Supplementary Table 2.
Reporting on race, ethnicity, or other socially relevant groupings	The present study did not incorporate race, ethnicity, or other socially-defined demographic categories in its design or analysis.
Population characteristics	The mean age of the abdominal aortic aneurysm (AAA) group was 63.5 ± 4.504 years. This cohort consisted of 7 male patients (87.5%), with a mean body mass index of 25.03 ± 1.061 kg/m² and maximum aortic diameter of 6.3 ± 1.697 cm. Comorbidities included hypertension (75.0%), smoking history (37.5%), hyperlipidemia (37.5%), and diabetes mellitus (12.5%). Patient characteristics are reported in Table 1.
Recruitment	Human aortic samples were collected from the Nanfang Hospital of Southern Medical University using protocols approved by the hospital's institutional review board. Human AAA samples were obtained from patients diagnosed with AAA according to the 2022 ACC/AHA Guidelines for the Diagnosis and Management of Aortic Diseases and who underwent aortic repair, with an abdominal aortic diameter ≥ 55 mm. Control aortic samples were collected from adjacent non-aneurysmal segments of the same patient.
Ethics oversight	All human samples were used with the approval of the Ethical Committee of Nanfang Hospital (approval number: NFEC-2023-476), and written informed consent was obtained from all participants. Participants were not compensated for the use of discarded tissue samples or clinical data. Participant sex was determined based on self-report.

Note that full information on the approval of the study protocol must also be provided in the manuscript.

Field-specific reporting

Please select the one below that is the best fit for your research. If you are not sure, read the appropriate sections before making your selection.

☒ Life sciences ☐ Behavioural & social sciences ☐ Ecological, evolutionary & environmental sciences

For a reference copy of the document with all sections, see nature.com/documents/nr-reporting-summary-flat.pdf

Life sciences study design

All studies must disclose on these points even when the disclosure is negative.

Sample size	No sample size calculation was performed. Experimental group sizes were determined based on established literature references and observed variability from prior laboratory studies. Total sample numbers (n) for all experimental groups, including in vivo and in vitro investigations, are detailed in respective figure legends.
Data exclusions	Mice that died from aortic rupture were not included in the aortic diameter measurements but were considered in the analysis of AAA incidence. Mice that died from aortic rupture were not included in the subsequent statistical analyses of aortic diameter, serum analysis, elastin degradation.
Replication	Experimental reproducibility was confirmed through multiple independent replicates, as indicated by sample size (n) values throughout

Replication	figures.
Randomization	Randomization protocols were implemented for experimental cohort assignment.
Blinding	For all animal and cell studies, investigators were blinded to group assignments during data collection and analysis, with sample processing order randomized. Blinding was not feasible during acquisition of human aortic tissues and clinical data as group allocation required consideration of inherent pathological characteristics.

Reporting for specific materials, systems and methods

We require information from authors about some types of materials, experimental systems and methods used in many studies. Here, indicate whether each material, system or method listed is relevant to your study. If you are not sure if a list item applies to your research, read the appropriate section before selecting a response.

Materials & experimental systems

n/a	Involved in the study
☐	☒ Antibodies
☐	☒ Eukaryotic cell lines
☒	☐ Palaeontology and archaeology
☐	☒ Animals and other organisms
☒	☐ Clinical data
☒	☐ Dual use research of concern
☒	☐ Plants

Methods

n/a	Involved in the study
☒	☐ ChIP-seq
☒	☐ Flow cytometry
☒	☐ MRI-based neuroimaging

Antibodies

Antibodies used	Western blot:<br>PDK4 (1:2000 dilution, 12949-1-AP, ProteinTech), PDK1 (1:1000 dilution; 18262-1-AP), PDK2 (1:500 dilution; 15647-1-AP), PDK3 (1:500 dilution; 12215-1-AP), ACTA2 (1:20,000 dilution, 67735-1-Ig, ProteinTech), CNN1 (1:2000 dilution, 24855-1-AP, ProteinTech), SM22α (1:5000 dilution, 10493-1-AP, ProteinTech), Alpha Tubulin (1:20000 dilution, 66031-1-Ig, ProteinTech), TXNIP (1:500 dilution, 18243-1-AP, ProteinTech), TRX (1:1000 dilution, 14999-1-AP, ProteinTech), KLF4 (1:1000 dilution, ab215036, Abcam), KLF5 (1:500 dilution, ab137676, Abcam), GAPDH (1:500 dilution, ab8245, Abcam), NLRP3 (1:100 dilution, ab263899, Abcam), p-PDHE1α (1:1000 dilution, ab177461, Abcam), PDHE1α (1:1000 dilution, ab168379, Abcam), Cleaved Caspase1 (1:1000 dilution, #89332, Cell Signaling Technology), Cleaved IL-1β (1:1000 dilution, #63124, Cell Signaling Technology), GSDMD (1:1000 dilution, #39754, Cell Signaling Technology), N-GSDMD (1:1000 dilution, #10137, Cell Signaling Technology), Cleaved IL-18 (1:1000 dilution, A24057, Abclonal).<br><br>Immunofluorescence:<br>PDK4 (1:50 dilution, 12949-1-AP, ProteinTech), ACTA2 (1:100 dilution, 67735-1-Ig, ProteinTech), CNN1 (1:50 dilution, 24855-1-AP, ProteinTech), SM22α (1:200 dilution, 10493-1-AP, ProteinTech), NLRP3 (1:200 dilution, 30109-1-AP, ProteinTech), GSDMD (1:50 dilution, 20770-1-AP, ProteinTech).<br><br>Immunoprecipitation:<br>TXNIP (18243-1-AP, Proteintech), Rabbit IgG isotype control (30000-0-AP, Proteintech)
Validation	All antibodies were commercially sourced and manufacturer-validated.

Eukaryotic cell lines

Policy information about cell lines and Sex and Gender in Research

Cell line source(s)	Human aortic smooth muscle cells (HASMCs) were acquired from Procell (CP-H081). Human umbilical vein endothelial cells (HUVECs) and AC16 cells were acquired from Cell Bank of the Chinese Academy of Sciences (PSC-01; SCSP-555).
Authentication	Cell line identities were routinely verified through Western blot and immunofluorescence marker analysis.
Mycoplasma contamination	All cell lines were confirmed mycoplasma-negative.
Commonly misidentified lines<br>(See ICLAC register)	No commonly misidentified cell lines were employed.

Animals and other research organisms

Policy information about studies involving animals; ARRIVE guidelines recommended for reporting animal research, and Sex and Gender in Research

Laboratory animals	To generate PDK4 conditional knockout mice, we purchased PDK4-flox (Pdk4fl/fl) mice from Cyagen Biosciences (Suzhou, China)

Laboratory animals	which contain loxP sites flanking exons 4, 5, and 6 of PDK4. Two gRNAs (gRNA1: 5′-CTAGATAGAAATTGTCACAT-3′; gRNA2: 5′-CCTTCTCACAATGTTATCCA-3′) were synthesized and inserted into a gRNA expression vector. This vector was then combined with Cas9 mRNA and single-stranded donor DNAs and microinjected into zygotes from C57BL/6J mice. PCR was used to confirm the successful introduction of the two loxP inserts into the target sites in the founder mice. These founders were subsequently bred with heterozygous Pdk4fl+/- mice to produce homozygous Pdk4fl/fl mice. Myh11-Cre mice [B6.FVB-Tg (Myh11-icre/ERT2)1Soff/J; stock no. 019079] were purchased from Jackson Laboratory (Bar Harbor, ME, USA). Myh11-Cre/Pdk4fl/fl (Pdk4SMKO) mice were generated by crossbreeding Pdk4fl/fl mice with Myh11-Cre mice. To induce the cre-expression, Myh11-Cre/Pdk4fl/fl mice and Pdk4fl/fl control mice were administrated intraperitoneally with tamoxifen (T5648, Sigma-Aldrich) at a dose of 75mg/kg/day for 5 consecutive days. All mice were maintained under a 12-hour light/dark cycle at 23 °C and 50-70% relative humidity. Detailed information on the animal experiments is provided in Supplementary Table 4.
Wild animals	No wild animals were utilized in this research.
Reporting on sex	The study employed male C57BL/6 mice aged 8 weeks. The Myh11-Cre mice [B6.FVB-Tg (Myh11-icre/ERT2)1Soff/J; stock no. 019079] were purchased from Jackson Laboratory (Bar Harbor, ME, USA). In this strain, the Myh11-Cre transgene is a Y-linked inheritance pattern. This Mendelian transmission mechanism ensures that Cre recombinase expression is strictly limited to XY male offspring, as females (XX constitution) inherently lack the Y chromosome required for transgene transmission. Consequently, our vascular smooth muscle cells specific PDK4 knockout model (Pdk4SMKO) exclusively manifests in male cohorts.
Field-collected samples	This study did not employ field-collected samples.
Ethics oversight	All animal protocols were approved by the Animal Research policies of the Southern Medical University Committee in Nanfang Hospital (approval number NFYY-2021-1260) and followed the Guide for the Care and Use of Laboratory Animals of the National Institute of Health in China.

Note that full information on the approval of the study protocol must also be provided in the manuscript.

Plants

Seed stocks	N/A
Novel plant genotypes	N/A
Authentication	N/A
