# Supplementary material for: PDK4 drives abdominal aortic aneurysm by promoting smooth muscle cell metabolic reprogramming and NLRP3-mediated pyroptosis
Source: Nat Commun. 2026 Apr 11;17:5086. doi: 10.1038/s41467-026-71610-w (PMC13246965; doi:10.1038/s41467-026-71610-w)
Supplement: Supplementary file 2 — Peer Review File [file 41467_2026_71610_MOESM2_ESM.pdf]

nature portfolio

Peer Review File

PDK4 drives abdominal aortic aneurysm by promoting smooth muscle cell metabolic reprogramming and NLRP3-mediated pyroptosis

Corresponding Author: Professor Xiu Liu

This file contains all reviewer reports in order by version, followed by all author rebuttals in order by version.

Version 0:

Reviewer comments:

Reviewer #1

(Remarks to the Author)
Zhao et al. presented an interesting study indicating the pivotal role of PDK4 in promoting smooth muscle cell metabolic reprogramming and NLRP3-mediated pyroptosis, which significantly contributes to the progression of abdominal aortic aneurysm (AAA). Although the theme looks promising, the study presents major concerns regarding the novelty of its findings and substantial methodological issues.
1) The role of the PDK/PDH axis in AAA development has been recently revealed (Griepke et al. 2025, Cardiovasc Res), including its modulation of SMCs. This prior publication is not cited in the current work, raising concerns about the novelty of current findings.
2) The study’s reliance on potentially weak single-cell RNA sequencing data to justify focusing on PDK4 contrasts with earlier research identifying PDK1 as a significant isoenzyme in SMCs and AAA formation. These conflicting results should have been considered. Moreover, while the study emphasizes the upregulation of PDK4 using the AngII model, it neglects to address the roles of other PDK isoforms, which could also significantly impact AAA.
3) A major methodological shortfall is the absence of a comparison of PDK4SMKO mice with littermate controls. This omission limits the ability to conclusively attribute observed phenotypic differences to PDK4 deletion, undermining the study’s validity and robustness of the conclusions. Additionally, although the authors used PCSK9-AAV and a Western diet (no info about source and composition is given), they provided no cholesterol data, which is a critical oversight. Hence, it is unclear whether tamoxifen was administered to the Pdk4fl/fl mice or only Cre. This information is crucial for validating the experimental design.
4) Authors use a PDK4-AAV strategy to upregulate PDK4 expression but do not sufficiently explore or verify the specificity of this vector for targeting aortic tissues, nor do they investigate potential off-target effects in other organs.
5) The efficiency of the siRNA used to silence PDK4 in culture experiments was not adequately quantified, casting doubt on the reliability of the gene-silencing effects observed.
6) The UMAP plots (Fig1A) and corresponding graphical data in Figure 1B appear unclear. There are studies that demonstrate how to subcluster SMCs to show their different repertoires, which could have been utilized to delineate better which cells express PDK4.
7) Figures 2G and 2H display stark phenotypic differences between Pdk4fl/fl and Pdk4SMKO mice, with the former showing significant plaque formation absent in the latter. This observation suggests PDK4’s crucial role in atherosclerosis. Is this the explanation for the differences between groups in AAA?
8) Figure 3 shows that AAV-mediated PDK4 overexpression markedly exacerbates intimal lesion formation, an effect not observed with the control AAV-NC. Could the reduction in atherosclerosis be the primary reason for the reduction in AAA here too?
9) Considering the previous points, in the AAA analysis and representative pictures, is the comparison made at similar anatomical levels?

Reviewer #2

(Remarks to the Author)
In this study, Zhao et all investigated the role of cellular metabolism regulator pyruvate dehydrogenase kinase 4 (PDK4) in

the development and progression of abdominal aortic aneurysm (AAA). The authors demonstrate that PDK4 expression is elevated in human AAA tissues and mouse models. Using vascular smooth muscle cell (VSMC)-specific PDK4 knockout mice, they show that PDK4 deletion reduces AAA formation by preventing VSMC the contractile-to-synthetic phenotypic switch, mitochondrial dysfunction, and NLRP3 inflammasome-driven pyroptosis. Mechanistically, PDK4 alters mitochondrial respiration in VSMCs, increases mitochondrial reactive oxygen species (ROS) production, and activates the NLRP3 inflammasome, leading to pyroptosis and vascular inflammation. Conversely, PDK4 overexpression exacerbates AAA progression. The NLRP3 inhibitor MCC950 attenuates AAA development in a PDK4-dependent manner. The study concludes that targeting PDK4 or NLRP3 could offer therapeutic benefits for AAA.
This study addresses an important and clinically relevant question with a strong experimental framework and compelling findings. However, key mechanistic and translational gaps remain. Addressing the following concerns will substantially strengthen the manuscript.

Major Concerns
1. The authors observed elevated PDK4 in AAA tissues. However, the mechanisms underlying this upregulation is not investigated. Although "point mutations in the PDK4 gene" is proposed, but not well supported by data. Without clear understanding of the regulatory mechanisms of PDK4 upregulation, the related therapeutic strategies may lack specificity and fail to correct the root of the problem.
2. The study is quite extensive and includes the effects of PDK4 on VSMC phenotypic switch, mitochondrial dysfunction, and NLRP3 inflammasome. However, the study is very descriptive because of a lack of mechanisms in how these different aspects are connected. The proposed link is not particularly novel
3. Figure 1 is this dataset derived from a single AAA sample without an organ donor control? The authors should clarify whether appropriate controls were included and provide more detailed evidence or analysis to support the claim of cell type-specific PDK4 expression. If no controls were used, this raises concerns about the validity of the conclusions—particularly how the authors define PDK4 as "highly expressed" in SMCs or ECs within AAA based solely on scRNA-seq data.
4. Figure 1I, the authors used AAV-PCSK9DY/Ang II model. It is not clear why authors used SMCs from Apoe-/- mice.
5. Figure 1E, the immunostaining is not convincing, as the tissue morphology appears compromised, with disrupted cell-cell architecture, which raises concerns about tissue preservation and staining reliability.
6. Figure 4A, includes only Pdk4SMKO-AngII and Pdk4fl/fl -AngII groups data, Pdk4SMKO-Vehicle and Pdk4fl/fl - Vehicle mice data should also be presented.
7. The authors utilize KLF4 and KLF5 as markers of synthetic VSMC phenotype, but these are not mentioned in the manuscript. Although the data suggests that PDK4 deficiency in VSMCs prevented the phenotypic switch from a contractile to a synthetic phenotype, the evidence is not sufficient to strongly support the protective role of VSMC-PDK4 deficiency in against AAA development.
8. Figure 4E, IF staining of mouse SR tissues showed that contractile markers ACTA2, CNN1 and SM22a in Pdk4SMKO mice showed significantly higher expression, but they are nearly absent in Pdk4fl/fl mice after AAV-PCSK9DY/Ang II treatment. Given that these are common VSMC markers, complete loss is biologically unlikely. Negative control images saline treated Pdk4SMKO mice and Pdk4fl/fl mice should be included for comparison. The same issue applies to Figures 7D/E and 8G/I, where saline-treated controls are essential to validate the observed expression patterns.
9. Figure 6, the experimental conditions—such as the concentration and duration of Ang II treatment in MASMCs (e.g., 1 µM for 48 h)—should be clearly stated in the main manuscript at the beginning of the relevant section to improve reproducibility and clarity.
10. The study links PDK4-driven mitochondrial ROS are implicated as upstream activators to NLRP3 but does not clarify how ROS triggers NLRP3.
11. While NLRP3 inhibitor MCC950 shows efficacy in mice, pharmacological targeting of PDK4 (PDK4 inhibitors, such as AZD7545, DPI) could be repurposed for AAA?

Minor Comments
1. Please add molecular weight markers to all western blot figures to improve data interpretation.
2. Include a schematic diagram in Figure 1 to illustrate the AAV-PCSK9^D377Y/Ang II model. This should clearly show mouse strain, age, diet, duration of treatment, and timeline of interventions.
3. The study uses male mice exclusively, which is consistent with the higher prevalence of AAA in males. However, sex-specific differences in AAA mechanisms should be briefly discussed or acknowledged.
4. To provide broader context, consider referencing recent single-cell RNA-seq or ATAC-seq datasets in AAA research. This may help support the cell type-specific findings and highlight PDK4's potential relevance in broader transcriptional landscapes.

Reviewer #3

(Remarks to the Author)
The manuscript by Zhao Li and co-workers, showing the effects of PDK4 on smooth muscle cell pyroptosis and the progression of abdominal aortic aneurysm (AAA), as well as its relevant molecular mechanisms. In general, the topic is novel. The authors provide convincing results regarding the promoting effects of PDK4 on abdominal aortic aneurysm formation. However, the link between PDK4-mediated mitochondrial reprogramming and pyroptosis and the progression of AAA are not very clear. The fine molecular mechanisms should be elucidated. There are several issues that should be addressed:
1. In Fig. 1F, PDK4 bands of western blot in the Ang II group is not clear.
2. In Fig. 1H, the suprarenal abdominal aortas from the Ang II group do not seem to be in the same proportion.
3. In Fig. 2, the author found that VSMC-PDK4 deficiency plays a protective role against AAA development. Whether

inhibition of the PDK4 activity, such as DCA could also lead to the same results?
4. The authors performed RNA-seq on MASMCs transfected with Ad-PDK4 or Ad-Con to investigate the mechanisms of PDK4 and VSMC functions in AAA pathogenesis (Figure S6A). Why not use the MASMCs isolated from Pdk4SMKO mice and Pdk4fl/fl mice in response to Ang II treatment to investigate the role of PDK4 in AAA pathogenesis.
5. In Fig. 5A-E, the basal OCR in MASMCs isolated from Pdk4SMKO mice is increased compared with the MASMCs from Pdk4fl/fl mice. In contrast, the basal ECAR in MASMCs isolated from Pdk4SMKO mice is decreased compared with the MASMCs from Pdk4fl/fl mice. These evidences probably suggested that Pdk4, but not Pdk1, Pdk2, and Pdk3, is involved in the mitochondrial respiration in wide-type mice. Whether the expression of the Pdk1, Pdk2, and Pdk3 are compensatory upregulated in Pdk4SMKO mice, and involved in the regulation of mitochondrial metabolism.
6. mitoSOX in the MASMCs isolated from Pdk4SMKO mice and the MASMCs from Pdk4fl/fl mice in the Saline group should also be detected.
7. The OXPHOS is increased in MASMCs from Pdk4SMKO mice compared with MASMCs from Pdk4fl/fl mice in response to Ang II treatment. Whether increased OXPHOS activity could promote mitochondrial ROS production.
8. In Fig. 6, DEG analysis indicated that inflammation and apoptosis-related genes were markedly increased with PDK4 overexpression. The author mentioned that “Previous studies emphasized the crucial role of pyroptosis in AAA. However, several references also reported that apoptosis of SMCs could also result in the development of AAA. The author should provide more comprehensive evidence to illustrate the pyroptosis, but not apoptosis, is mainly involved in the AAA pathogenesis.
9. The authors found that PDK4 promotes glycolysis and reduces the OXPHOS in VASMC, which indicates that PDK4 enzyme plays an important role in mitochondrial respiration. Meanwhile, the authors further found that PDK4 promotes pyroptosis in VSMCs. Therefore, the effect of PDK4 in AAA is dependent on its enzyme activity or function as a regulator of AAA Not related to its enzyme activity ?
10. The PDK4-mediated ROS generation in AAA is only associated with metabolic reprogramming? Whether other factors, such as PDK4-mediated apoptosis or inflammation, could also result in the ROS generation? If so,the alteration of mitochondrial respiration by PDK4 in AAA may not be important.
11. Whether PDK4-mediated ROS generation could result in AAA directly，rather than through NLRP3-mediated pyroptosis in VSMCs ?
12. PDK4 deletion in VSMCs reduces mitochondrial ROS production and suppresses VSMC pyroptosis. Is there a parallel or causal relationship between ROS and pyroptosis ?
13. The association between pyroptosis and AAA in this article could be explained more clearly. Inhibition of NLRP3 inflammasome activation did not fully explain that PDK4-mediated pyroptosis promotes the AAA progression.

Version 1:

Reviewer comments:

Reviewer #1

(Remarks to the Author)
The revised manuscript by Zhao et al. has improved substantially, and the authors have addressed most of the previous concerns. The inclusion of additional validation experiments and a more detailed description of the experimental design is appreciated. That said, several important issues remain that should be carefully considered.

1) The revised version appropriately cites prior work on the PDK/PDH axis (Griepke et al., 2025) and specifies that their approach, using VSMC-specific genetic models and a different AAA model, provides a more cell-specific and mechanistic perspective. However, although the authors argue in their reply that the identification of PDK4 rather than PDK1 may partly reflect model differences (PPE vs. AAV-PCSK9/Ang II), this point has not been explored or discussed in the revised manuscript. Acknowledging such model-dependent factors would help contextualize their findings and prevent overstating the specificity of PDK4 to AAA. Thus, the claim that this work "significantly advances the field" should be moderated.

2) The authors identify PDK4 as the dominant isoform in AAA, but this conclusion should be tempered. Griepke et al., 2025 showed, using three independent human datasets and one murine dataset, that PDK1 was a dominantly expressed isoform in AAA. Hence, the current study's reliance on a single scRNA-seq dataset does not seem sufficient to disregard these prior findings. Moreover, as the authors have not experimentally excluded possible roles of other isoforms in their own system, the overall message of the manuscript aligns with a broader PDK/PDH axis role rather than a specific PDK4-driven mechanism.

3) The new Oil Red O data (included in the rebuttal letter) showing comparable plaque burden between genotypes are important and should be fully integrated into the manuscript.

4) Finally, a major concern remains, as the SMC-specific genetic targeting was conducted without the use of littermate controls. The explanation provided is understandable from a logistical standpoint but not sufficient to exclude potential effects of background genetic variability, which can substantially influence vascular phenotypes. In the absence of littermate comparisons, the authors should provide evidence of genetic background equivalence, for example, through verification such as microsatellite or SNP genotyping.

Reviewer #2

(Remarks to the Author)
The authors have made substantial revisions and addressed majority of previous concerns through additional experiments and improved figure presentation. Authors have included additional single-cell RNA-seq analysis and new experimental evidence, which together provide more mechanistic link. The inclusion of pharmacological inhibition data using DCA further supports the conclusion that PDK4 enzymatic activity contributes to AAA progression. Overall, the manuscript has been improved significantly and the conclusions are supported by solid evidence.
Minor Comments:
1. Add a scale bar to Figure 1L.
2. The term “prevent AAA” may overstate the therapeutic effect. Consider consistently using terms such as “attenuate” or “reduce severity” to better reflect pharmacodynamic accuracy.
3. The font size in the schematic illustration (Figure 10I) is relatively small; increasing the font size will improve readability.

Reviewer #3

(Remarks to the Author)
The manuscript written by Zhao Li and co-workers, showing the effects of PDK4 on smooth muscle cell pyroptosis and the progression of abdominal aortic aneurysm (AAA), as well as its relevant molecular mechanisms.
Overall, this paper has certain innovativeness. The author and his colleagues have made careful revisions to the paper. The questions raised by the reviewers have been responded to one by one. It can be considered that the author has completed the basic revision tasks.
However, the link between PDK4-mediated mitochondrial reprogramming and pyroptosis and the progression of AAA is to be investigated furtherly.

Version 2:

Reviewer comments:

Reviewer #1

(Remarks to the Author)
The authors have responded appropriately to my previous comments, and the tone of the Conclusions has been notably improved. However, during the revision process, a new high impact study has been published demonstrating that VSMC-specific ablation of Pdk1 in the PCSK9-AAV/hyperlipidemia plus Ang II model confers protection against AAA (Liu et al., Circulation, PMID: 41487086), suggesting a mechanistic contribution distinct from that proposed here and in other studies.

On page 23, the authors state: “This divergence is likely influenced by the intrinsic pathophysiological differences between the two models…” and further argue that model-dependent inflammatory, metabolic, and hemodynamic contexts may preferentially engage different PDK isoforms. While this interpretation is reasonable, the recent evidence directly implicating PDK1 in VSMC-driven AAA development indicates that these findings should be more thoroughly integrated into the discussion.

Therefore, it would be important for the authors not only to cite this new study but also to reconcile it conceptually with their current framework. Rather than emphasizing individual PDK isoforms in isolation, the authors may consider framing alterations as changes in the PDK/PDH axis as the central mechanistic theme. Such an approach would better accommodate the emerging evidence that both PDK1 (in two studies) and PDK4 could be functionally relevant, influencing immunity, VSMCs and vascular disease.

I have no other comments.

Open Access This Peer Review File is licensed under a Creative Commons Attribution 4.0 International License, which permits use, sharing, adaptation, distribution and reproduction in any medium or format, as long as you give appropriate credit to the original author(s) and the source, provide a link to the Creative Commons license, and indicate if changes were made.
In cases where reviewers are anonymous, credit should be given to 'Anonymous Referee' and the source.
The images or other third party material in this Peer Review File are included in the article's Creative Commons license, unless indicated otherwise in a credit line to the material. If material is not included in the article's Creative Commons license and your intended use is not permitted by statutory regulation or exceeds the permitted use, you will need to obtain permission directly from the copyright holder.
To view a copy of this license, visit https://creativecommons.org/licenses/by/4.0/

Response to reviewer #1

Reviewer #1: Zhao et al. presented an interesting study indicating the pivotal role of PDK4 in promoting smooth muscle cell metabolic reprogramming and NLRP3-mediated pyroptosis, which significantly contributes to the progression of abdominal aortic aneurysm (AAA). Although the theme looks promising, the study presents major concerns regarding the novelty of its findings and substantial methodological issues.
1) The role of the PDK/PDH axis in AAA development has been recently revealed (Griepke et al. 2025, *Cardiovasc Res*), including its modulation of SMCs. This prior publication is not cited in the current work, raising concerns about the novelty of current findings.

Response:
We sincerely appreciate the reviewer's insightful comment. While Griepke et al. (2025) provided valuable contributions to understanding the PDK/PDH axis in abdominal aortic aneurysm (AAA), our study significantly advances the field in several key aspects, as outlined below:

1.Cell-type-specific genetic manipulation and pharmacological inhibition
Griepke et al. used dichloroacetate (DCA), a pan-PDK inhibitor, which lacks isoform and cell-type specificity. In contrast, our study employed vascular smooth muscle cell (VSMC)-specific PDK4 knockout ($Pdk4^{SMKO}$) and VSMC-specific PDK4 overexpression mice (AAV-PDK4), enabling precise dissection of PDK4's role in VSMCs. This approach avoids confounding effects from other cell types (e.g., macrophages) and PDK isoforms (PDK1–3), offering mechanistic clarity.

2.Different AAA Model
Griepke et al. utilized the porcine pancreatic elastase (PPE)-induced AAA model, which involves acute elastic lamellae fragmentation and medial leukocyte infiltration, triggering rapid aneurysm expansion (300–400% diameter increase within a week) via intraluminal thrombus (ILT)-mediated protease release (MMP-2/9, plasmin) and neutrophil activation. This progresses through two phases: initial elastic recoil loss from direct elastase damage, followed by leukocyte-driven inflammation and clot formation. By weeks 2–3, stabilization occurs as the ILT fibroses. Key limitations include high variability in dilation/rupture rates, dependence on surgical skill and elastase batch purity, and potential confounding effects from gelatinase contaminants in PPE preparations.
Our study adopted the AAV-$PCSK9^{DY}$/Ang II model combined with a Western diet, which recapitulates two major AAA risk factors: hyperlipidemia and hypertension. This model is widely used in previous studies ***(1. The Nonproteolytic Intracellular Domain of Membrane-Type 1 Matrix Metalloproteinase Coordinately Modulates Abdominal Aortic Aneurysm and Atherosclerosis in Mice-Brief Report. Arteriosclerosis, Thrombosis, and Vascular Biology. Published online 8 September, 2022.***

doi:10.1161/ATVBAHA.122.317686. 2. Hypercholesterolemia Induced by a PCSK9 Gain-of-Function Mutation Augments Angiotensin II-Induced Abdominal Aortic Aneurysms in C57BL/6 Mice-Brief Report. Arteriosclerosis, Thrombosis, and Vascular Biology. Published online 28 July, 2016. doi:10.1161/ATVBAHA.116.307613. 3. KLF11 protects against abdominal aortic aneurysm through inhibition of endothelial cell dysfunction. JCI Insight. Published online 8 March, 2021. doi: 10.1172/jci.insight.141673.*)**. The Ang II-induced AAA model initiates with intimal tears that allow blood penetration into the aortic wall, forming intramural hematomas and leading to external dilation. This process is characterized by early medial phagocyte accumulation, elastic fiber degradation, and T/B-lymphocyte infiltration. Ang II triggers region-specific VSMCs responses—hyperplasia in the ascending aorta and hypertrophy elsewhere—highlighting heterogeneous aortic remodeling. Additionally, Ang II activates endothelial cells via nitric oxide signaling and NF-κB nuclear translocation, upregulating adhesion molecules and promoting monocyte/macrophage recruitment, further exacerbating inflammation and vascular dysfunction ***(Translational Relevance and Recent Advances of Animal Models of Abdominal Aortic Aneurysm. Arteriosclerosis, Thrombosis, and Vascular Biology. Published online 5 January, 2017. doi:10.1161/ATVBAHA.116.308534.).

3.Identified PDK4 as the dominant isoform

Our reanalysis of mouse AAA single-cell RNA-seq data (GSE239620) identified PDK4 as the most upregulated isoform in VSMCs. Moreover, PDK4 (but not PDK1-3) showed the most significant differential expression in AAA tissues compared to control tissues (**Figure 1A**). The most pronounced differential expression of PDK4 between AAA and control groups was specifically observed in VSMCs (**Figure 1B**). Moreover, the results indicated that PDK4, but not PDK1, PDK2 or PDK3, exhibited the most significant differential expression in VSMCs in AAA tissues (**Figure 1C**). Western blot showed that there were no significant changes of PDK1, PDK2, or PDK3 in AAA tissues from mice treated with AAV-$PCSK9^{DY}$/Ang II for 28 days compared to those treated with AAV-$PCSK9^{DY}$/saline for 28 days and PDK4 was most highly expressed in VSMCs (**Figure S1G-S1H**). This selective upregulation suggests that PDK4 may play a distinct role in AAA pathogenesis. Given the essential role of PDK family members in cellular metabolism, global PDK inhibition would likely cause significant metabolic derangements. In contrast, specifically targeting the PDK4 isoform, which shows selective upregulation in AAA, may offer a more tailored therapeutic intervention with an improved safety profile. By focusing on this specific isoform, we can develop targeted inhibition approaches that minimize off-target effects while potentially achieving better therapeutic efficacy against AAA progression. Moreover, we demonstrated that PDK4 expression is upregulated in AAA tissues in humans and mice (**Figure 1E-1K**). PDK4 deletion in VSMCs effectively suppressed AAA development (**Figure 2**). Our study provides an isoform-specific resolution by identifying PDK4 as the dominant, differing from the pan-PDK inhibitory approach employed by Griepke et al., which targeted all PDK isoforms indiscriminately. Whereas their broad inhibition

strategy may have obscured the unique role of individual isoforms, our findings clearly demonstrate that PDK4, but not PDK1, PDK2, or PDK3, is significantly upregulated in VSMCs in AAA. Functional studies further confirmed that PDK4 deletion in VSMCs effectively suppressed AAA development. Thus, our work highlights the limitation of pan-PDK inhibition and advocates for PDK4-specific targeting as a more precise and potentially safer therapeutic strategy for AAA.

4.Novel mechanistic insights: PDK4/ROS/NLRP3/Pyroptosis Axis
Our study establishes that PDK4 promoted metabolic reprogramming in VSMCs, leading to mitochondrial respiration alterations, activation of the NLRP3 inflammasome, and pyroptosis, which worsened inflammation and AAA progression. These findings reveal a novel PDK4-NLRP3-pyroptosis axis in AAA pathogenesis that was not investigated in the study by Griepke et al., which primarily examined neutrophil infiltration and activation and VSMC phenotype changes without exploring the specific roles of PDK/PDH in programmed cell death pathways or metabolic reprogramming of VSMCs.

5.Therapeutic validation with MCC950 or DCA
Our study further validated the PDK4/NLRP3 axis as a therapeutically targetable pathway by demonstrating that both MCC950 (a selective NLRP3 inhibitor) and DCA (a pan-PDK inhibitor) significantly attenuated PDK4-driven AAA progression in AAV-*PCSK9*^{DY}/Ang II mouse models (**Figures 9 and 10**). In contrast, Griepke et al.'s study, which lacked VSMC-specific genetic manipulations (e.g., conditional knockout or overexpression), did not include such experiments to confirm the mechanistic link between PDK isoforms and AAA pathogenesis. Our use of both pharmacological and genetic approaches thus provides a more rigorous validation of this target. Please refer to the revised manuscript on **line 91-98, Page 5 and line 367-382, Page 17-18**.

2) The study’s reliance on potentially weak single-cell RNA sequencing data to justify focusing on PDK4 contrasts with earlier research identifying PDK1 as a significant isoenzyme in SMCs and AAA formation. These conflicting results should have been considered. Moreover, while the study emphasizes the upregulation of PDK4 using the AngII model, it neglects to address the roles of other PDK isoforms, which could also significantly impact AAA.

Response:
We appreciate the reviewer’s insightful comment regarding the potential discrepancy between our focus on PDK4 and prior studies emphasizing PDK1 in VSMCs and AAA pathogenesis. We agree that these differences warrant clarification, and we highlight three key factors that may contribute to the conflicting results:

1.Specific functional validation of PDK4 in AAA pathogenesis overcomes the limitations of Pan-PDK analyses
Griepke et al. demonstrated elevated PDH-pSer293 levels in human AAA tissues. This

modification is biochemically governed primarily by PDK2, which exhibit superior catalytic efficiency toward Ser293 (catalytic efficiency: PDK2 > PDK4 ≈ PDK1 > PDK3) (***Therapeutic Targeting of the Pyruvate Dehydrogenase Complex/Pyruvate Dehydrogenase Kinase (PDC/PDK) Axis in Cancer. Journal of the National Cancer Institute. Published online 1 November, 2017. doi: 10.1093/jnci/djx071.***). In contrast, PDK1 shows lower efficiency at Ser293, as its primary phosphorylation site on PDH is Ser232 (***Pyruvate dehydrogenase kinase regulates vascular inflammation in atherosclerosis and increases cardiovascular risk. Cardiovascular Research. Published online 4 July, 2023. doi:10.1093/cvr/cvad038.***). Furthermore, Griepke et al. did not experimentally validate PDK2/4 contributions in AAA models, and did not utilize VSMC-specific knockout of PDK isoforms to assess their causal roles in AAA pathogenesis *in vivo*. Instead, they relied solely on dichloroacetate (DCA), a pan-PDK inhibitor that non-selectively targets all isoforms, in the PPE-induced AAA model. Consequently, the claim of PDK1's primacy in AAA pathogenesis appears inadequately supported by phosphosite-specific biochemical evidence and lacks isoform-resolved functional validation.

2.Divergent AAA models underlying mechanistic differences
Griepke et al. employed the porcine pancreatic elastase (PPE)-induced AAA model, which primarily triggers inflammation and elastin degradation, whereas our study utilized the AAV-*PCSK9*^{DY}/Ang II model combined with a Western diet—a system that recapitulates key features of human AAA pathophysiology, including hyperlipidemia, hypertension, and progressive aneurysm formation. These distinct models likely differentially regulate metabolic pathways (e.g., PDK1 vs. PDK4) due to variations in hemodynamic stress, lipid metabolism, and inflammatory responses, potentially explaining the divergent emphasis on specific isoenzymes across studies.

3.Fundamental differences in data sources and analytical methodologies
The discrepancy between our findings and those of Griepke et al. may stem from fundamental differences in the underlying datasets and analytical focus. Griepke et al.'s conclusions integrated bulk transcriptomic data from human AAA and control aortic tissues (microarray: Biros et al., Lindquist Liljeqvist et al.; RNA-seq: Temprano-Sagrera et al.), which reflect average gene expression across heterogeneous cell types within the whole tissue. In contrast, our analysis leveraged single-cell RNA-seq data from mouse AAA and control aortic tissues (GEO: GSE239620; Sheng Le et al.), which allowed us to focus specifically on VSMCs. This shift in resolution from whole-tissue to a specific cell type coupled with differences in species and technical methodology, likely explains the distinct outcomes and underscores the enhanced specificity of our approach in identifying PDK4's role in AAA pathogenesis.

Moreover, to address potential compensatory roles of other PDK isoforms, we performed Western blot analysis of PDK1, PDK2, PDK3 in aortic tissues from AAV-*PCSK9*^{DY}/Ang II mouse models. Critically, no significant changes in PDK1, PDK2 or PDK3 protein levels were observed in AAA tissues compared to those treated with

AAV-*PCSK9*^{DY}/saline (**Figure S1G**). Similarly, the western blot results showed that there were no significant differences in the levels of PDK1, PDK2, PDK3 in Vehicle or Ang II-treated mouse aortic smooth muscle cells (MASMCs) from *Pdk4*^{SMKO} mice compared to those from *Pdk4*$^{fl/fl}$ mice (**Figure S5J**). The detailed results have been included in the revised manuscript **(lines 99-102, page 5 and lines 240-245, page 11-12**). This confirms that PDK4 upregulation is isoform-specific in our experimental systems and suggests that other PDKs do not compensatorily contribute to AAA pathogenesis under these conditions.

3) A major methodological shortfall is the absence of a comparison of PDK4SMKO mice with littermate controls. This omission limits the ability to conclusively attribute observed phenotypic differences to PDK4 deletion, undermining the study's validity and robustness of the conclusions. Additionally, although the authors used PCSK9-AAV and a Western diet (no info about source and composition is given), they provided no cholesterol data, which is a critical oversight. Hence, it is unclear whether tamoxifen was administered to the Pdk4fl/fl mice or only Cre. This information is crucial for validating the experimental design.

Response:

We appreciate the reviewer's insightful comment. While we acknowledge that littermate comparisons are ideal, the large-scale design of our study (requiring multiple parallel cohorts for phenotypic and mechanistic analyses) made it logistically impractical to obtain sufficient littermate controls (*Pdk4*^{SMKO} and *Pdk4*$^{fl/fl}$ mice). To ensure rigorous comparability, all *Pdk4*^{SMKO} and *Pdk4*$^{fl/fl}$ mice were strictly matched for age, blood pressure, body weight, and plasma lipid levels, and maintained under identical housing conditions. Detailed data and analysis can be found in the **Figure S3E-H**. Additionally, as described in the Methods section (**Page 29, Lines 623-625**), the Western diet used in our study (D12492; Jiangsu Xietong Pharmaceutical Bio-engineering) contained 40% kcal fat, 10.5% kcal sucrose, and 1.25% cholesterol, a formulation well-established for inducing hyperlipidemia in mouse models. Regarding the AAV-*PCSK9*^{DY} system, we employed the pAAV/D374Y-hPCSK9 (*PCSK9*^{DY}) plasmid (Addgene #58379, kindly provided by Dr. Bentzon), which was packaged into AAV8 via co-transfection with helper plasmids in HEK293T cells. Viral titers were quantified by PCR, and mice received a single tail-vein injection of 2×10^{11} vector genomes/mouse (**Page 30, Lines 651-657**). To clarify, both *Pdk4*^{SMKO} and *Pdk4*$^{fl/fl}$ mice received tamoxifen injections (75 mg/kg/day, intraperitoneally, for five consecutive days) to activate Cre recombinase in the experimental group while maintaining identical treatment conditions across genotypes. This design controls for potential off-target effects of tamoxifen itself, ensuring that the observed phenotypes are specifically attributable to PDK4 deletion in VSMCs. This important detail has now been explicitly added to the revised Methods section. (**Page 28, Lines 603**).

4) Authors use a PDK4-AAV strategy to upregulate PDK4 expression but do not

sufficiently explore or verify the specificity of this vector for targeting aortic tissues, nor do they investigate potential off-target effects in other organs.

Response:
We appreciate the reviewer's concern regarding the specificity of AAV-*PDK4* targeting. As demonstrated in **Figure S4C-D**, Western blot and immunofluorescence analyses confirmed that PDK4 expression was significantly upregulated in VSMCs of mice injected with AAV-*PDK4* compared to AAV-NC controls. Furthermore, since our AAV9 vector carries a GFP tag, we performed additional validation to confirm its specific transduction in aortic tissues. As shown in **Figure S4A**, immunofluorescence analyses demonstrated successful transduction of GFP-labeled AAV9 into aortic tissues in mice injected with either AAV-*PDK4* or AAV-NC, thereby validating the efficacy and specificity of our delivery system for aortic tissue. Furthermore, to address potential off-target effects, we systematically examined PDK4 expression in liver, lung, heart, kidney and aorta by Western blot. The results showed that PDK4 levels were selectively elevated in the aorta of AAV-*PDK4*-treated mice, with no detectable changes in other organs compared to AAV-NC controls (**Figure S4B**). These data support the vascular specificity of our AAV-*PDK4* strategy and argue against significant off-target effects in non-aortic tissues. Please refer to the revised manuscript on **line 152-162, Page 7-8**.

5) The efficiency of the siRNA used to silence PDK4 in culture experiments was not adequately quantified, casting doubt on the reliability of the gene-silencing effects observed.

Response:
We appreciate the reviewer's concern regarding the quantification of PDK4 silencing efficiency. As shown in **Figure S6G,** Western blot analysis confirmed that siR-*PDK4* achieved approximately 74% knockdown efficiency compared to the scrambled siRNA control (siR-Con), validating the effectiveness of our gene-silencing approach. This result demonstrates robust and reproducible PDK4 silencing in our cultured cells, supporting the reliability of subsequent functional assays.

6) The UMAP plots (Fig1A) and corresponding graphical data in Figure 1B appear unclear. There are studies that demonstrate how to subcluster SMCs to show their different repertoires, which could have been utilized to delineate better which cells express PDK4.

Response:
We thank the reviewer for this insightful suggestion. Following re-analysis of the single-cell RNA-seq data from mouse AAA and control aortic tissues (GEO: GSE239620; Sheng Le et al.), the results showed that PDK4 expression is predominantly highest in VSMCs, followed by fibroblasts and endothelial cells (**Figure S1C-S1D**). The most pronounced differential expression of PDK4 between AAA and control groups was specifically observed in VSMCs (**Figure 1B**). Further subclustering

of VSMCs identified six distinct subpopulations: Contractile_VSMC, Fibroblast-like_VSMC, Immune-like_VSMC1, Immune-like_VSMC2, Proliferating_VSMC and Stressed_VSMC (**Figure S1E-S1F**). PDK4 expression was predominantly elevated in the Fibroblast-like_VSMC subpopulation (**Figure 1D**). Additionally, the re-analyzed t-SNE plots and associated graphical data now provide the clearest possible resolution of these expression patterns. Please refer to the revised manuscript on **line 86-99, Page 4-5**.

7) Figures 2G and 2H display stark phenotypic differences between Pdk4fl/fl and Pdk4SMKO mice, with the former showing significant plaque formation absent in the latter. This observation suggests PDK4's crucial role in atherosclerosis. Is this the explanation for the differences between groups in AAA?

Response:

We sincerely appreciate the reviewer's attention to this detail. To clarify, PDK4 deletion in VSMCs does not significantly affect atherosclerosis (AS) plaque formation, and thus cannot explain the observed differences in AAA progression between groups. Our results show: 1. As shown in **Figure 2B**, no morphological differences in AS plaques between *Pdk4*^{SMKO} and *Pdk4*$^{fl/fl}$ mice. 2. Oil Red O staining of aortic roots revealed comparable plaque areas in *Pdk4*^{SMKO} and *Pdk4*$^{fl/fl}$ mice (**Fig. A**). 3. The initial representative image of *Pdk4*$^{fl/fl}$ mice (showing plaques) was replaced to avoid misinterpretation, as plaque presence was incidental and not PDK4-dependent. Please refer to the revised **Figure 2G-H**.

The AAA phenotype differences are therefore independent of atherosclerosis, driven instead by PDK4-mediated mitochondrial ROS-NLRP3-pyroptosis axis in VSMCs. We thank the reviewer for prompting this clarification.

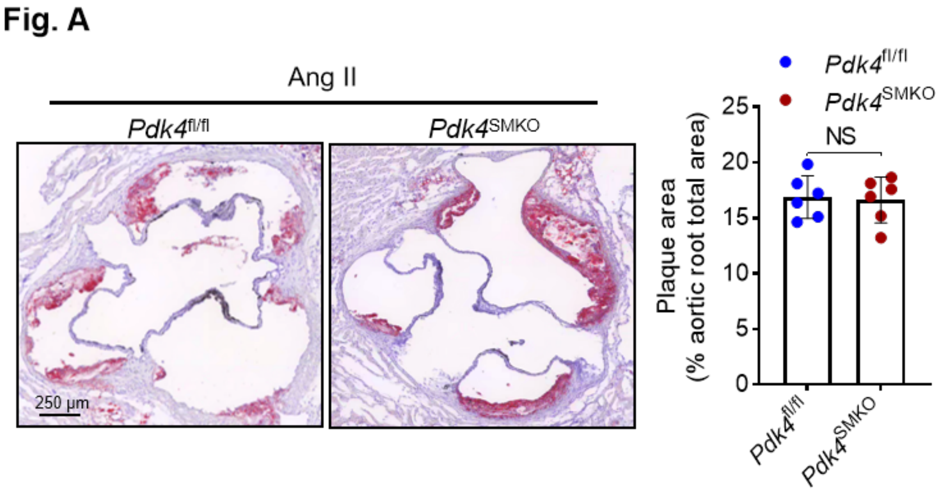

Fig. A Representative Oil Red O-stained aortic root sections and quantification in *Pdk4*$^{fl/fl}$ and *Pdk4*^{SMKO} mice (n=6). Unpaired Student's *t*-test was used. NS, nonsignificant.

8) Figure 3 shows that AAV-mediated PDK4 overexpression markedly exacerbates

intimal lesion formation, an effect not observed with the control AAV-NC. Could the reduction in atherosclerosis be the primary reason for the reduction in AAA here too?

Response:

We sincerely appreciate the reviewer's insightful comment. To clarify, PDK4 overexpression in VSMCs does not exacerbate AS pathogenesis, and thus cannot account for the observed AAA progression differences. Our results demonstrate: 1. As shown in **Figure 3B**, no substantial AS plaque difference between AAV-*PDK4* and AAV-NC mice. 2. Oil Red O staining of aortic roots revealed comparable plaque areas in AAV-*PDK4* and AAV-NC mice (**Fig. B**). 3. The initial representative "intimal lesion" in AAV-*PDK4* group was identified as a thrombus (non-plaque structure) via Oil Red O staining, and we have updated the representative figure to avoid misinterpretation. Please refer to the revised **Figure 3G-H**. Thank you for highlighting this critical point.

Fig. B

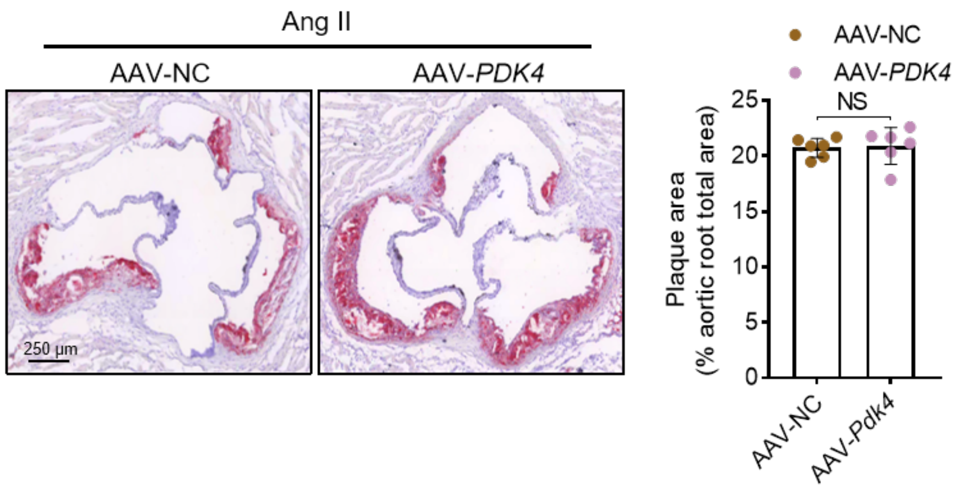

Fig. B Representative Oil Red O-stained aortic root sections and quantification in AAV-NC and AAV-*PDK4* mice (n=6). Unpaired Student's t-test was used. NS, nonsignificant.

9) Considering the previous points, in the AAA analysis and representative pictures, is the comparison made at similar anatomical levels?

Response:

We appreciate the reviewer's attention to methodological consistency in our AAA analyses. To ensure anatomical comparability, all measurements and representative images were specifically obtained from the suprarenal abdominal aorta in both *Pdk4*$^{fl/fl}$ and *Pdk4*^{SMKO} or AAV-NC and AAV-*PDK4* mice. This standardized approach, described in each corresponding figure legend, guarantees that all comparisons were performed at identical anatomical levels.

Response to reviewer #2

Reviewer #2: In this study, Zhao et all investigated the role of cellular metabolism regulator pyruvate dehydrogenase kinase 4 (PDK4) in the development and progression of abdominal aortic aneurysm (AAA). The authors demonstrate that PDK4 expression is elevated in human AAA tissues and mouse models. Using vascular smooth muscle cell (VSMC)-specific PDK4 knockout mice, they show that PDK4 deletion reduces AAA formation by preventing VSMC the contractile-to-synthetic phenotypic switch, mitochondrial dysfunction, and NLRP3 inflammasome-driven pyroptosis. Mechanistically, PDK4 alters mitochondrial respiration in VSMCs, increases mitochondrial reactive oxygen species (ROS) production, and activates the NLRP3 inflammasome, leading to pyroptosis and vascular inflammation. Conversely, PDK4 overexpression exacerbates AAA progression. The NLRP3 inhibitor MCC950 attenuates AAA development in a PDK4-dependent manner. The study concludes that targeting PDK4 or NLRP3 could offer therapeutic benefits for AAA.
This study addresses an important and clinically relevant question with a strong experimental framework and compelling findings. However, key mechanistic and translational gaps remain. Addressing the following concerns will substantially strengthen the manuscript.

Major Concerns
1. The authors observed elevated PDK4 in AAA tissues. However, the mechanisms underlying this upregulation is not investigated. Although "point mutations in the PDK4 gene" is proposed, but not well supported by data. Without clear understanding of the regulatory mechanisms of PDK4 upregulation, the related therapeutic strategies may lack specificity and fail to correct the root of the problem.

Response:
We appreciate the reviewer's valid concern regarding the mechanisms underlying PDK4 upregulation in AAA. To address this potential mechanism directly, we performed searches for PDK4-associated single nucleotide polymorphisms (SNPs) using publicly available GWAS data and the FinnGen biobank database, specifically focusing on associations with AAA. However, our analysis did not identify significant AAA-associated SNPs within the PDK4 gene locus (as shown in the figure below). This suggests that common genetic variants in PDK4 itself are unlikely to be a primary driver of its elevated expression in AAA in the cohorts analyzed thus far.

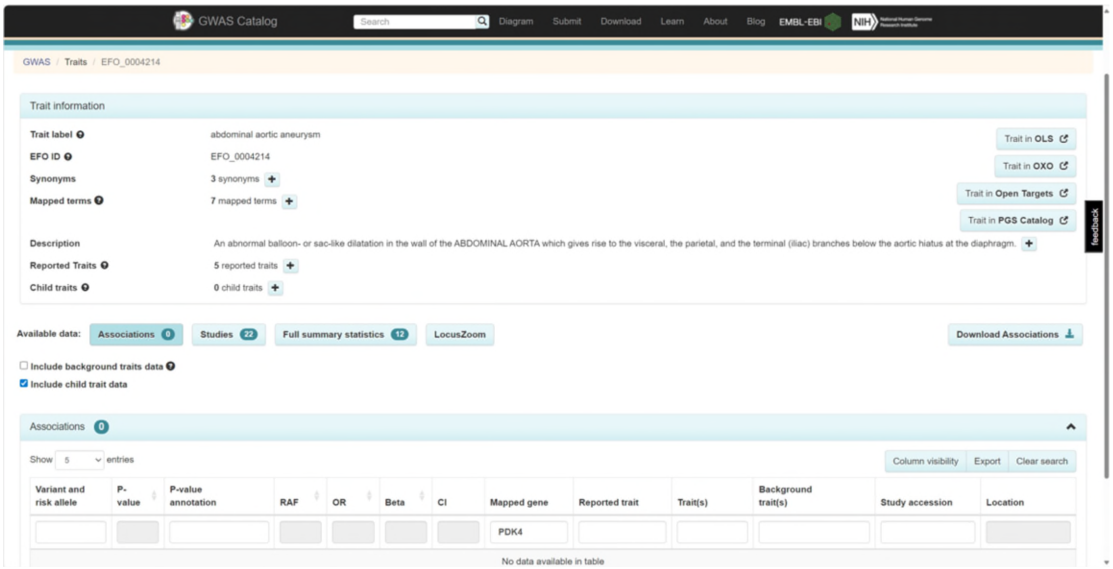

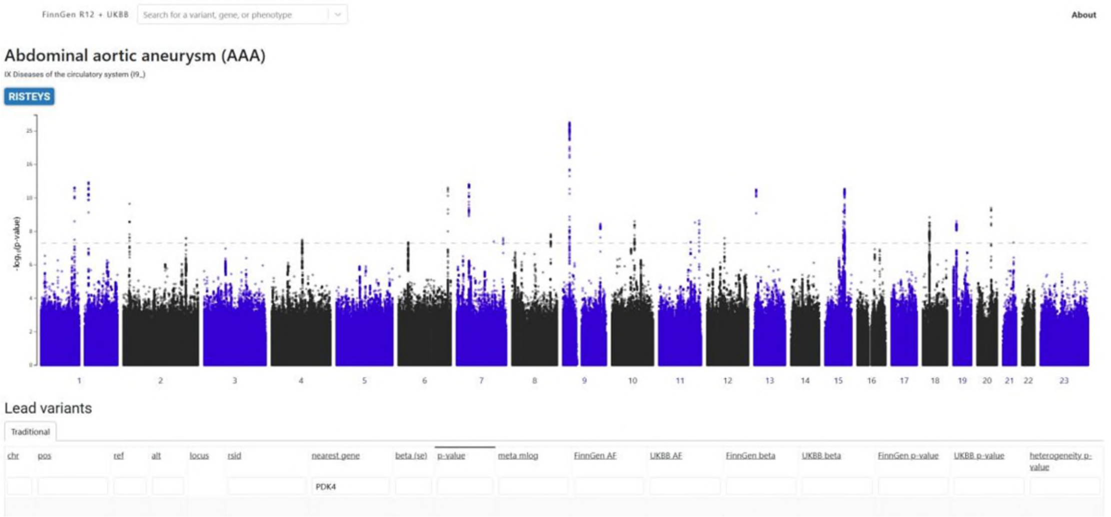

Moreover, recent studies have indicated that activation of the PI3K/AKT pathway suppresses PDK4 expression ***(FGF21 protects against HFpEF by improving cardiac mitochondrial bioenergetics in mice. Nature Communications. Published online 15 February, 2025. doi: 10.1038/s41467-025-56885-9.)***. Given that the PI3K/AKT pathway has been reported to play a key role in AAA ***(Mesenchymal stem cell-derived extracellular vesicles protect against abdominal aortic aneurysm formation by inhibiting NET-induced ferroptosis. Experimental & Molecular Medicine. Published online 1 May, 2023. doi: 10.1038/s12276-023-00986-2.)***, we used the PI3K inhibitor LY294002 to examine PDK4 expression. Our results showed that LY294002 increased the mRNA levels of PDK4, suggesting a possible regulatory link (**Figure S1I**). Please refer to the revised manuscript on **line 102-106, Page 5**. We fully agree that further mechanistic studies are needed and plan to conduct a more targeted analysis in future work to better elucidate the upstream regulation of PDK4. We will conduct genomic DNA sequencing on blood samples collected from well-phenotyped AAA patients and matched controls. This will enable specific analysis of PDK4-related SNPs (including rare variants and those in regulatory regions) and their potential association with both AAA development and PDK4 expression levels. This approach provides a direct

assessment of genetic contributions within our study population. We thank the reviewer for the valuable comments.

2. The study is quite extensive and includes the effects of PDK4 on VSMC phenotypic switch, mitochondrial dysfunction, and NLRP3 inflammasome. However, the study is very descriptive because of a lack of mechanisms in how these different aspects are connected. The proposed link is not particularly novel

Response:
We sincerely appreciate the reviewer's valuable perspective. Our data demonstrate that the phenotypic switch is a consequence, not a cause, of underlying molecular events. MCC950 treatment significantly increased contractile markers and decreased synthetic markers compared to saline treatment, supporting that NLRP3 inflammasome activation drives the phenotypic switch (**Figure S9K**). Rotenone treatment reversed the reduction in the expression of GSDMD and N-GSDMD in MASMCs induced by PDK4 deficiency, and this effect was blocked by MCC950 **(Figure 7G).** Moreover, through re-analysis of the single-cell RNA-seq data (GEO: GSE239620), we identified six VSMC subpopulations: Contractile_VSMC, Fibroblast-like_VSMC, Immune-like_VSMC1, Immune-like_VSMC2, Proliferating_VSMC, and Stressed_VSMC (**Figure S1E-S1F**). PDK4 was significantly upregulated in Fibroblast-like_VSMCs, showing the most pronounced differential expression compared to Contractile_VSMCs (**Figure 1D**). To establish mechanistic connections, we quantified pyroptosis and mitochondrial respiration pathway activities in these subpopulations using the AUCell algorithm, revealing that Fibroblast-like_VSMCs exhibited elevated pyroptosis pathway activity and glycolysis pathway activity but suppressed mitochondrial respiration pathway activity compared to Contractile_VSMCs (**Figure 5N-5O and 6N**). Gene Set Enrichment Analysis (GSEA) further demonstrated significant correlation between NOD-like receptor signaling and AAA progression in VSMCs (**Figure 6L**). Moreover, we identified TXNIP as the molecular linker: PDK4-mediated alteration of mitochondrial respiration triggers thioredoxin-interacting protein (TXNIP) dissociation from thioredoxin (TRX), thereby promoting TXNIP-NLRP3 binding and subsequent NLRP3 inflammasome assembly (**Figure 7H-7J**). Combining with our experimental finding that PDK4 is upregulated in AAA, these results directly couple PDK4-mediated metabolic remodeling to NLRP3-dependent pyroptosis, providing a mechanism connecting VSMC phenotypic switch to AAA progression. Please refer to the revised manuscript on **line 96-199, Page 5, 218-221, Page 10-11, 288-293, Page 14 and line 332-340, Page 16**.

3. Figure 1 is this dataset derived from a single AAA sample without an organ donor control? The authors should clarify whether appropriate controls were included and provide more detailed evidence or analysis to support the claim of cell type-specific PDK4 expression. If no controls were used, this raises concerns about the validity of the conclusions—particularly how the authors define PDK4

as "highly expressed" in SMCs or ECs within AAA based solely on scRNA-seq data.

Response:
We acknowledge the reviewer's valid concern regarding controls in our original single-cell RNA-seq dataset (GSE166676), where the limited cell yield from control samples precluded robust comparative analysis. To rigorously address this, we have now re-analyzed a published dataset with integrated mouse AAA and control aortic tissues (GEO: GSE239620; Sheng Le et al.). This integrated analysis confirmed that PDK4 expression is predominantly highest in VSMCs, followed by fibroblasts and endothelial cells (**Figure S1C-S1D**). The most pronounced differential expression of PDK4 between AAA and control groups was specifically observed in VSMCs (**Figure 1B**). This cell-type-specific pattern was further validated at the protein level via Western blot, demonstrating highest PDK4 expression in VSMCs (**Figure S1H**). Importantly, subclustering of VSMCs revealed six distinct subpopulations, with PDK4 expression most significantly elevated in the Fibroblast-like_VSMC (**Figure S1E-S1F and 1D**). Collectively, these analyses robustly confirm PDK4's specific enrichment in VSMC subpopulations within AAA.

4. Figure 1I, the authors used AAV-PCSK9DY/Ang II model. It is not clear why authors used SMCs from Apoe-/- mice.

Response:
We appreciate the reviewer's inquiry regarding the use of SMCs from *Apoe*$^{-/-}$ mice in our study. To clarify, we initially validated PDK4 upregulation in SMCs isolated from the AAV-*PCSK9*^{DY}/Ang II model as confirmed by qPCR, Western blot, and immunofluorescence analysis (**Figure 1L and S2B-S2C**). The additional experiments using *Apoe*$^{-/-}$ -derived SMCs served as a complementary model to reinforce the generality of PDK4's role across different hyperlipidemic backgrounds. In future studies, we will further validate the role of PDK4 in AAA using *Pdk4*$^{fl/fl}$ *Apoe*$^{-/-}$ mice and *Pdk4*^{SMKO} *Apoe*$^{-/-}$ double knockout mice. This approach aligns with our goal to ensure robustness by demonstrating PDK4 dysregulation in multiple AAA-relevant settings. Please refer to the revised manuscript on **line 123-128, Page 6**.

5. Figure 1E, the immunostaining is not convincing, as the tissue morphology appears compromised, with disrupted cell-cell architecture, which raises concerns about tissue preservation and staining reliability.

Response:
We sincerely appreciate the reviewer's careful evaluation of Figure 1E. We acknowledge that the original immunostaining image may have raised concerns due to suboptimal tissue morphology. To address this, we have now replaced the representative fluorescence image with a higher-quality AAA sample, ensuring clear visualization of cellular architecture and specific staining signals. Please refer to the revised **Figure 1G**.

6. Figure 4A, includes only Pdk4SMKO-AngII and Pdk4fl/fl -AngII groups data, Pdk4SMKO-Vehicle and Pdk4fl/fl - Vehicle mice data should also be presented.

Response:

We sincerely appreciate the reviewer's suggestion to include the vehicle control data for comprehensive comparison. In response, we have now supplemented the RT-qPCR results from both *Pdk4*^{SMKO} + Vehicle and *Pdk4*$^{fl/fl}$ + Vehicle groups. RT-qPCR analysis showed that mRNA levels of contractile markers (ACTA2, CNN1, and SM22α) were significantly increased, while mRNA levels of synthetic markers (KLF4, KLF5) were significantly decreased in vehicle or Ang II-treated MASMCs from *Pdk4*^{SMKO} mice compared to those from *Pdk4*$^{fl/fl}$ mice. Comprehensive data and analysis are presented in the revised **Figure 4A** and can be found in the updated manuscript, specifically on lines **174-177, page 8-9**.

7. The authors utilize KLF4 and KLF5 as markers of synthetic VSMC phenotype, but these are not mentioned in the manuscript. Although the data suggests that PDK4 deficiency in VSMCs prevented the phenotypic switch from a contractile to a synthetic phenotype, the evidence is not sufficient to strongly support the protective role of VSMC-PDK4 deficiency in against AAA development.

Response:

We sincerely appreciate the reviewer's valuable suggestion regarding the clarification of KLF4 and KLF5 as markers of the synthetic VSMC phenotype. In response to this comment, we have now expanded the manuscript to include a detailed discussion of VSMC phenotypic markers and their functional significance in both physiological and pathological contexts. Please refer to the revised manuscript on line **491-510, Page 23-24.** Furthermore, re-analysis of the single-cell RNA-seq dataset (GEO: GSE239620) revealed that Fibroblast-like_VSMCs (where PDK4 is most highly expressed) exhibited elevated pyroptosis pathway activity and glycolysis pathway activity but suppressed mitochondrial respiration pathway activity compared to Contractile_VSMCs (**Figure 5N-5O and 6N**). This distinct pathway activity signature demonstrates the pro-inflammatory and metabolically dysregulated nature of PDK4-enriched VSMC subpopulations in AAA. This finding, complemented by our *in vivo* evidence that PDK4 deletion in VSMCs mitigates AAA in mice, collectively indicates that PDK4 deficiency prevents the transition toward this detrimental phenotype and confers protection against AAA development.

8. Figure 4E, IF staining of mouse SR tissues showed that contractile markers ACTA2, CNN1 and SM22a in Pdk4SMKO mice showed significantly higher expression, but they are nearly absent in Pdk4fl/fl mice after AAV-PCSK9DY/Ang II treatment. Given that these are common VSMC markers, complete loss is biologically unlikely. Negative control images saline treated Pdk4SMKO mice and Pdk4fl/fl mice should be included for comparison. The same issue applies to

Figures 7D/E and 8G/I, where saline-treated controls are essential to validate the observed expression patterns.

Response:

We sincerely appreciate the reviewer's insightful feedback regarding the immunofluorescence staining in Figure 4E, 7D/E and 8G/I. We agree that the complete absence of contractile markers (ACTA2, CNN1, SM22α) in *Pdk4*$^{fl/fl}$ mice after AAV-*PCSK9*^{DY}/Ang II treatment may appear biologically counterintuitive. Upon re-examination, we identified that the merged channel images (particularly the DAPI signal intensity in some areas) partially obscured the weaker fluorescence signals of these markers in *Pdk4*$^{fl/fl}$ mice after AAV-*PCSK9*^{DY}/Ang II treatment, creating an impression of near-total loss. To resolve this ambiguity, we have replaced the original figure panels with new representative images (**Figures 4E, 7D/E, and 8I**) in order to avoid suggesting a biologically implausible loss of markers. Furthermore, as suggested, we have now added saline-treated control images for **Figures 7D/E and 8G/I** to validate the observed expression patterns under baseline conditions, similarly demonstrating the protective effect of PDK4 deficiency against AAA.

9. Figure 6, the experimental conditions—such as the concentration and duration of Ang II treatment in MASMCs (e.g., 1 μM for 48 h)—should be clearly stated in the main manuscript at the beginning of the relevant section to improve reproducibility and clarity.

Response:

We sincerely appreciate the reviewer's constructive suggestion regarding the clarification of experimental conditions. In response, we have now explicitly stated the MASMC treatment parameters (1 μM Ang II for 48 h) in the main manuscript at the beginning of the relevant section. This revision ensures full transparency and reproducibility of our RNA-seq and functional analyses. Please refer to the revised manuscript on line **249-263, Page 12-13**.

10. The study links PDK4-driven mitochondrial ROS are implicated as upstream activators to NLRP3 but does not clarify how ROS triggers NLRP3.

Response:

We thank the reviewer for requesting clarification on how mitochondrial ROS activates NLRP3. Under oxidative stress, ROS accumulation triggers thioredoxin-interacting protein (TXNIP) dissociation from the TXNIP-TRX complex, enabling TXNIP translocation to bind NLRP3 and initiate inflammasome. Our experiments demonstrate that MitoQ treatment attenuated PDK4-induced TXNIP upregulation and restored TRX levels in MASMCs (**Figure 7H**). Co-immunoprecipitation further confirmed significantly weakened TXNIP-TRX binding and conversely enhanced TXNIP-NLRP3 interaction in PDK4-overexpressing MASMCs (**Figures 7I-7J**). This TXNIP-mediated ROS-NLRP3 axis is well-established in prior studies ***(1. Thioredoxin-interacting***

protein links oxidative stress to inflammasome activation. Nature Immunology. Published online 20 December, 2009. doi:10.1038/ni.1831. 2. Reactive oxygen species promote tubular injury in diabetic nephropathy: The role of the mitochondrial ros-txnip-nlrp3 biological axis. Redox Biology. Published online 15 February, 2018. doi:10.1016/j.redox.2018.02.013. 3. STING-IRF3 contributes to lipopolysaccharide-induced cardiac dysfunction, inflammation, apoptosis and pyroptosis by activating NLRP3. Redox Biology. Published online 13 May, 2019. doi: 10.1016/j.redox.2019.101215.), and our work extends this mechanism to PDK4-driven AAA pathogenesis. Please refer to the revised manuscript on line **334-343, Page 16**.

11. While NLRP3 inhibitor MCC950 shows efficacy in mice, pharmacological targeting of PDK4 (PDK4 inhibitors, such as AZD7545, DPI) could be repurposed for AAA?

Response:

We thank the reviewer for this insightful suggestion. Multiple studies have employed AZD7545 to inhibit PDK1 ***(1. Distinct structural mechanisms for inhibition of pyruvate dehydrogenase kinase isoforms by AZD7545, dichloroacetate, and radicicol. Structure. Published online 2 August, 2007. doi: 10.1016/j.str.2007.07.001. 2. ROS production induced by BRAF inhibitor treatment rewires metabolic processes affecting cell growth of melanoma cells. Molecular Cancer. Published online 8 June, 2017. doi: 10.1186/s12943-017-0667-y.***), while DPI is a NOX inhibitor ***(NADPH oxidase 1: A target in the capacity of dimeric ECG and EGCG procyanidins to inhibit colorectal cancer cell invasion. Redox Biology. Published online 25 July, 2023. doi: 10.1016/j.redox.2023.102827.***). Thus, to directly evaluate pharmacological PDK4 inhibition as a therapeutic strategy for AAA, we employed dichloroacetate (DCA), a well-established inhibitor of PDK activity, as widely utilized in previous studies targeting PDK4 ***(1. Pyruvate dehydrogenase kinase 4 deficiency attenuates cisplatin-induced acute kidney injury. Kidney International. Published online 28 December, 2016. doi: 10.1016/j.kint.2016.10.011. 2. Inhibition of Pyruvate Dehydrogenase Kinase 4 Protects Cardiomyocytes from lipopolysaccharide-Induced Mitochondrial Damage by Reducing Lactate Accumulation. Inflammation. Published online 24 February, 2024. doi: 10.1007/s10753-024-01981-z. 3. Dichloroacetate Prevents Sepsis Associated Encephalopathy by Inhibiting Microglia Pyroptosis through PDK4/NLRP3. Inflammation. Published online 23 August, 2024. doi:10.1007/s10753-024-02105-3.***). Our results demonstrate that DCA treatment robustly attenuated AAA progression. Compared to saline-treated controls, DCA significantly reduced AAA incidence and mortality. Furthermore, DCA effectively inhibited pathological aortic dilation and reduced elastin degradation. Collectively, our data demonstrate that DCA-mediated pharmacological inhibition of PDK4 mitigates AAA progression by directly targeting PDK4 activity, thereby validating PDK4 as a promising therapeutic target for AAA.Comprehensive data and analysis are presented in the revised **Figure 9** and can be found in the updated manuscript, specifically on **lines 367-382, page 17-18**.

Minor Comments

1. Please add molecular weight markers to all western blot figures to improve data interpretation.

Response:

We sincerely appreciate the reviewer's suggestion regarding the inclusion of molecular weight markers in our Western blot figures. In response, we have now added precise molecular weight markers (kDa) to each lane of all Western blot panels in the revised manuscript. These markers are clearly labeled and aligned with the corresponding protein bands to facilitate accurate data interpretation.

2. Include a schematic diagram in Figure 1 to illustrate the AAV-PCSK9^D377Y/Ang II model. This should clearly show mouse strain, age, diet, duration of treatment, and timeline of interventions.

Response:

We sincerely appreciate the reviewer's constructive suggestion to improve the clarity of our experimental design. In response, we have now added a detailed schematic diagram to **Figure 1G**, which clearly illustrates the AAV-*PCSK9*^{DY}/Ang II model, including: mouse strain, age, diet, duration of treatment, and timeline of interventions.

3. The study uses male mice exclusively, which is consistent with the higher prevalence of AAA in males. However, sex-specific differences in AAA mechanisms should be briefly discussed or acknowledged.

Response:

We appreciate the reviewer's insightful comment regarding sex-specific differences in AAA. Key risk factors for AAA include smoking status, genetic predisposition, male sex, and advancing age, with epidemiological studies showing a 4-6 times greater prevalence in males compared to females. ***(1.Genome-wide association meta-analysis identifies risk loci for abdominal aortic aneurysm and highlights PCSK9 as a therapeutic target. Nature Genetics. Published online 16 October, 2023. doi:10.1038/s41588-023-01510-y. 2.Abdominal aortic aneurysm: Sex differences. Maturitas. Published online 13 December, 2017. doi:10.1016/j.maturitas.2017.12.012.)***. In experimental models, male C57BL/6 mice exhibit greater susceptibility to AAA induction ***(Adiponectin attenuates abdominal aortic aneurysm formation in hyperlipidemic mice. Atherosclerosis. Published online 23 May, 2014. doi:10.1016/j.atherosclerosis.2014.05.923.)***. Importantly, the *Myh11*-Cre mice [B6.FVB-Tg (Myh11-icre/ERT2)1Soff/J; stock no. 019079] were purchased from Jackson Laboratory (Bar Harbor, ME, USA). In this strain, the *Myh11*-Cre transgene is a Y-linked inheritance pattern. This Mendelian transmission mechanism ensures that Cre recombinase expression is strictly limited to XY male offspring, as females (XX constitution) inherently lack the Y chromosome required for transgene

transmission. Consequently, our vascular smooth muscle cells specific PDK4 knockout model (*Pdk4*^{SMKO}) exclusively manifests in male cohorts. Please refer to the revised manuscript on line **417-419, Page 19-20**.

4. To provide broader context, consider referencing recent single-cell RNA-seq or ATAC-seq datasets in AAA research. This may help support the cell type-specific findings and highlight PDK4's potential relevance in broader transcriptional landscapes.

Response:

We thank the reviewer for this valuable suggestion. To provide broader transcriptional context, we reanalyzed a publicly available single-cell RNA-seq dataset (GSE239620) from mouse aortic tissues, originating from a recently published (2024) study in *Cardiovascular Research*, as detailed in our Results (**Figure 1A-1D, S1A-S1F, 5N-5O, and 6L-6N**). Our analysis revealed that: 1. PDK4 expression was highest in VSMCs, with the most pronounced differential expression between AAA and control groups also observed in this cell type (**Figure 1B**). 2.PDK4 expression is predominantly highest in VSMCs, followed by fibroblasts and endothelial cells (**Figure S1C-S1D**). 3.Unique isoform role: PDK4 (but not PDK1-3) exhibited the most significant differential expression in aortic tissues, particularly within VSMCs (**Figure 1A and 1C**). 4.Gene Set Enrichment Analysis (GSEA) further demonstrated significant correlation between NOD-like receptor signaling and AAA progression in VSMCs (**Figure 6L**). 5.Analysis of VSMC clusters identified six distinct subpopulations: Contractile_VSMC, Fibroblast-like_VSMC, Immune-like_VSMC1, Immune-like_VSMC2, Proliferating_VSMC, and Stressed_VSMC (**Figure S1E-S1F**). Compared to Contractile_VSMCs, Fibroblast-like_VSMCs exhibit the highest PDK4 levels, elevated pyroptosis pathway activity and glycolysis pathway activity but suppressed mitochondrial respiration activity (**Figure 1D, 5N-5O, and 6N**). 6. AAA groups exhibited higher pyroptosis pathway activity than Control groups (**Figure 6M**). This integrated analysis directly links PDK4-driven pyroptosis to a disease-associated VSMC phenotype within a mouse AAA scRNA-seq atlas.

Response to reviewer #3

Reviewer #3: The manuscript by Zhao Li and co-workers, showing the effects of PDK4 on smooth muscle cell pyroptosis and the progression of abdominal aortic aneurysm (AAA), as well as its relevant molecular mechanisms. In general, the topic is novel. The authors provide convincing results regarding the promoting effects of PDK4 on abdominal aortic aneurysm formation. However, the link between PDK4-mediated mitochondrial reprogramming and pyroptosis and the progression of AAA are not very clear. The fine molecular mechanisms should be elucidated. There are several issues that should be addressed:

1. In Fig. 1F, PDK4 bands of western blot in the Ang II group is not clear.

Response:

We sincerely appreciate the reviewer's careful observation regarding the clarity of the PDK4 Western blot bands in Figure 1F. In response, we have now replaced the representative western blot with a higher-quality image that clearly demonstrates PDK4 expression in the abdominal aortas of mice treated with AAV-*PCSK9*^{DY}/Ang II or Saline. Please refer to the revised **Figure 1I**.

2. In Fig. 1H, the suprarenal abdominal aortas from the Ang II group do not seem to be in the same proportion.

Response:

We sincerely appreciate the reviewer's attention to detail regarding the apparent size discrepancy in Figure 1H. To clarify, all immunofluorescence images were captured at the same magnification (scale bars are explicitly labeled in each figure). The seemingly larger aortic diameter in the Ang II group reflects pathological vascular dilation induced by Ang II. However, we acknowledge that the original representative image may have caused ambiguity. To address this, we have replaced the figure panel with a new representative image that maintains identical scaling while providing clearer visualization of vascular architecture. Please refer to the revised **Figure 1K**. We thank the reviewer for this valuable feedback.

3. In Fig. 2, the author found that VSMC-PDK4 deficiency plays a protective role against AAA development. Whether inhibition of the PDK4 activity, such as DCA could also lead to the same results?

Response:

We thank the reviewer for this critical question. To directly address whether pharmacological inhibition of PDK4 activity confers protection against abdominal aortic aneurysm (AAA), we evaluated the effects of dichloroacetate (DCA), a well-established PDK4 activity inhibitor. As detailed in **Figure 9**, wild-type mice subjected to AAV-*PCSK9*^{DY}/Ang II-induced AAA were treated with DCA. Our results definitively

demonstrate that DCA treatment robustly attenuated AAA progression. Compared to saline-treated controls, DCA significantly reduced AAA incidence and mortality. Furthermore, DCA effectively inhibited pathological aortic dilation and reduced elastin degradation. Critically, these outcomes mirror the protection observed in VSMC-specific PDK4-deficient mice. Collectively, our data confirm that pharmacological inhibition of PDK4 activity with DCA mitigates AAA progression through a mechanism dependent on PDK4 activity, thereby validating PDK4 as a druggable target for AAA therapy. The detailed results have been included in the revised manuscript (**lines 367-382, page 17-18**) and are presented in **Figure 9A-9H**.

4. The authors performed RNA-seq on MASMCs transfected with Ad-PDK4 or Ad-Con to investigate the mechanisms of PDK4 and VSMC functions in AAA pathogenesis (Figure S6A). Why not use the MASMCs isolated from Pdk4SMKO mice and Pdk4fl/fl mice in response to Ang II treatment to investigate the role of PDK4 in AAA pathogenesis.

Response:

We sincerely appreciate the reviewer's insightful suggestion to strengthen our mechanistic investigation. In the revised manuscript, we have now integrated the RNA-seq data from Ang II-treated *Pdk4*^{SMKO} and *Pdk4*$^{fl/fl}$ MASMCs (GEO: GSE305105), complementing our original analysis of Ad-*PDK4* MASMCs. KEGG pathway analysis revealed significant downregulation of key inflammatory pathways in *Pdk4*^{SMKO} MASMCs, including TNF signaling, IL-17 signaling, NOD-like receptor signaling and cytokine-cytokine receptor interactions pathways (**Figure S6E**). DEG analysis further confirmed marked suppression of critical inflammasome-related genes (Nlrp3, Aim2, Casp4, Nod2) (**Figure S6F**). These findings demonstrate that PDK4 drives AAA pathogenesis by promoting inflammasome activation. The detailed results have been included in the revised manuscript (**lines 262-267, page 12-13**) and are presented in **Figure S6D-S6F**.

5. In Fig. 5A-E, the basal OCR in MASMCs isolated from Pdk4SMKO mice is increased compared with the MASMCs from Pdk4fl/fl mice. In contrast, the basal ECAR in MASMCs isolated from Pdk4SMKO mice is decreased compared with the MASMCs from Pdk4fl/fl mice. These evidences probably suggested that Pdk4, but not Pdk1, Pdk2, and Pdk3, is involved in the mitochondrial respiration in wide-type mice. Whether the expression of the Pdk1, Pdk2, and Pdk3 are compensatory upregulated in Pdk4SMKO mice, and involved in the regulation of mitochondrial metabolism.

Response:

We thank the reviewer for raising this important point. To address whether compensatory upregulation of other PDK isoforms occurs in *Pdk4*^{SMKO} mice, we performed Western blot analysis of PDK1-3 in both vehicle and Ang II-treated MASMCs. Critically, no compensatory upregulation of PDK1, PDK2, or PDK3 was

observed in *Pdk4*^{SMKO} MASMCs compared to *Pdk4*$^{fl/fl}$. This confirms that PDK4 drives metabolic reprogramming and mitochondrial respiratory remodeling without compensatory PDK1-3 overexpression. The detailed results have been included in the revised manuscript (**lines 240-245, page 11-12**) and are presented in **Figure S5J**.

6. mitoSOX in the MASMCs isolated from Pdk4SMKO mice and the MASMCs from Pdk4fl/fl mice in the Saline group should also be detected.

Response:
We thank the reviewer for this valuable suggestion. As requested, we have now performed additional MitoSOX staining quantifying mitochondrial ROS levels in MASMCs isolated from *Pdk4*^{SMKO} mice and in MASMCs from *Pdk4*$^{fl/fl}$ mice under Vehicle conditions. The results reveal no significant difference in mitochondrial ROS levels between MASMCs isolated from *Pdk4*^{SMKO} mice and those isolated from *Pdk4*$^{fl/fl}$ mice under Vehicle conditions. Please refer to the revised **Figure 5Q**.

7. The OXPHOS is increased in MASMCs from Pdk4SMKO mice compared with MASMCs from Pdk4fl/fl mice in response to Ang II treatment. Whether increased OXPHOS activity could promote mitochondrial ROS production.

Response:
We appreciate the reviewer's insightful question regarding the relationship between increased OXPHOS activity and mitochondrial ROS production. As shown in Figure **5P and 5Q**, our data demonstrate that mitochondrial ROS levels under basal conditions remain unaltered in both *Pdk4*^{SMKO} aortic tissues and MASMCs compared to *Pdk4*$^{fl/fl}$ controls, despite PDK4 deficiency-driven OXPHOS enhancement. This suggests that the increased OXPHOS activity caused by PDK4 deficiency may be accompanied by efficient coupling of the electron transport chain (ETC), reducing electron leakage during electron transfer and thereby decreasing ROS generation. A number of studies have documented this phenomenon: ***(1. Nuclear Receptor NR1D1 Regulates Abdominal Aortic Aneurysm Development by Targeting the Mitochondrial Tricarboxylic Acid Cycle Enzyme Aconitase-2. Circulation. Published online 26 July, 2022. doi:10.1161/CIRCULATIONAHA.121.057623. 2. Hypoxia-induced BNIP3 facilitates the progression and metastasis of uveal melanoma by driving metabolic reprogramming. Autophagy. Published online 12 September, 2024. doi:10.1080/15548627.2024.2395142. 3. Mitochondrial Respiratory Chain Supercomplexes: From Structure to Function. International Journal of Molecular Sciences. Published online 10 November, 2022. doi: 10.3390/ijms232213880.)***.

8. In Fig. 6, DEG analysis indicated that inflammation and apoptosis-related genes were markedly increased with PDK4 overexpression. The author mentioned that "Previous studies emphasized the crucial role of pyroptosis in AAA. However, several references also reported that apoptosis of SMCs could also result in the development of AAA. The author should provide more comprehensive evidence to

illustrate the pyroptosis, but not apoptosis, is mainly involved in the AAA pathogenesis.

Response:

We appreciate the reviewer's constructive feedback. To clarify the predominant role of pyroptosis in AAA pathogenesis, we have provided the following evidence: 1.Western blot analysis revealed that the protein levels of NLRP3, Cleaved Caspase1, Cleaved IL-1β, Cleaved IL-18, GSDMD, and N-GSDMD were significantly increased in human AAA tissues obtained from patients undergoing abdominal aorta replacement surgery compared to control aortic tissues from a non-aneurysmal segment of the same patient (**Figure 6P**). 2. TEM analysis of Ang II-treated MASMCs showed that PDK4 deficiency attenuated pyroptosis hallmarks (cell swelling and membrane pore formation) (**Figure 6O**). 3. Re-analysis of the single-cell RNA-seq data revealed elevated pyroptosis pathway activity in AAA samples (**Figure 6M**). These findings further support pyroptosis as the mechanism regulated by PDK4.

9. The authors found that PDK4 promotes glycolysis and reduces the OXPHOS in VASMC, which indicates that PDK4 enzyme plays an important role in mitochondrial respiration. Meanwhile, the authors further found that PDK4 promotes pyroptosis in VSMCs. Therefore, the effect of PDK4 in AAA is dependent on its enzyme activity or function as a regulator of AAA Not related to its enzyme activity?

Response:

We thank the reviewer for this critical mechanistic inquiry. Our data definitively establish that PDK4 promotes abdominal aortic aneurysm (AAA) pathogenesis specifically through its enzymatic activity, as demonstrated by the protective effects of dichloroacetate (DCA), a well-established PDK4 activity inhibitor. As documented in prior structural studies, DCA directly suppresses PDK4 enzymatic activity and reduces PDH phosphorylation without altering total PDK4 protein expression (***1. Pyruvate dehydrogenase kinase 4 deficiency attenuates cisplatin-induced acute kidney injury. Kidney International. Published online 28 December, 2016. doi: 10.1016/j.kint.2016.10.011. 2. Inhibition of Pyruvate Dehydrogenase Kinase 4 Protects Cardiomyocytes from lipopolysaccharide-Induced Mitochondrial Damage by Reducing Lactate Accumulation. Inflammation. Published online 24 February, 2024. doi: 10.1007/s10753-024-01981-z.***). Our results, consistent with these findings, definitively demonstrate that DCA treatment robustly attenuated AAA progression through reduced PDH phosphorylation without altering total PDK4 protein expression (**Figure 9I**). Compared to saline-treated controls, DCA significantly reduced AAA incidence and mortality. Furthermore, DCA effectively inhibited pathological aortic dilation and reduced elastin degradation (**Figure 9A-9H**). If PDK4 contributed to AAA pathogenesis through non-enzymatic activity-dependent pathways, the inhibitory effect of DCA would likely be less pronounced. Thus, PDK4 promotes AAA pathogenesis through its enzymatic activity.

10. The PDK4-mediated ROS generation in AAA is only associated with metabolic reprogramming? Whether other factors, such as PDK4-mediated apoptosis or inflammation, could also result in the ROS generation? If so,the alteration of mitochondrial respiration by PDK4 in AAA may not be important.

Response:
We thank the reviewer for this important inquiry about the mechanistic relationship between PDK4-mediated ROS and metabolic reprogramming. Our data indicate that PDK4-driven mitochondrial ROS generation in abdominal aortic aneurysm is primarily attributable to metabolic reprogramming. This conclusion is supported by the evidence that DCA significantly attenuates mitochondrial ROS production induced by PDK4 overexpression in Ang II-stimulated MASMCs (**Figure 9J**). Moreover, our results demonstrate that both mitochondrial-targeted antioxidant MitoQ and general antioxidant NAC suppressed activation of NLRP3 inflammasome components (NLRP3, Cleaved Caspase1, Cleaved IL-1β, Cleaved IL-18) and inhibited pyroptosis executor (GSDMD, N-GSDMD) in MASMCs (**Figure 7B-C and S7A-S7B**). Rotenone treatment reversed the reduction in the expression of GSDMD and N-GSDMD in MASMCs induced by PDK4 deficiency, and this effect was blocked by MCC950 (**Figure 7G**). This confirms that PDK4-generated ROS acts mechanistically upstream of inflammasome activation. Crucially, these findings align with prior studies demonstrating that ROS serves as an upstream activator of PDK4-mediated apoptosis or inflammatory pathways ***(1. Reducing Oxidative Stress and Inflammation by Pyruvate Dehydrogenase Kinase 4 Inhibition Is Important in Prevention of Renal Ischemia-Reperfusion Injury in Diabetic Mice. Diabetes & Metabolism Journal. Published online 1 February, 2024. doi: 10.4093/dmj.2023.0196. 2. Dichloroacetate Prevents Sepsis Associated Encephalopathy by Inhibiting Microglia Pyroptosis through PDK4/NLRP3. Inflammation. Published online 23 August, 2024. doi:10.1007/s10753-024-02105-3. 3. PDK4 Decrease Neuronal Apoptosis via Inhibiting ROS-ASK1/P38 Pathway in Early Brain Injury After Subarachnoid Hemorrhage. Antioxidants & Redox Signaling. Published online 4 January, 2022. doi: 10.1089/ars.2021.0083.)***.

11.Whether PDK4-mediated ROS generation could result in AAA directly, rather than through NLRP3-mediated pyroptosis in VSMCs?

Response:
We appreciate this critical inquiry regarding the mechanistic role of PDK4-mediated ROS in AAA pathogenesis. Our key *in vivo* intervention experiment demonstrates that NLRP3 inhibition with MCC950 significantly attenuated AAA severity in PDK4-overexpressing mice, evidenced by reduced aortic dilation, decreased elastin degradation, suppressed pyroptosis markers, and improved survival rates (**Figure 10A-10H**). This direct functional evidence indicates that PDK4-driven AAA largely depends

on NLRP3-mediated pyroptosis in VSMCs. While ROS may theoretically exert NLRP3-independent effects, our data align with established literature where ROS-triggered NLRP3 activation is a dominant pathway in AAA ***(1. Activating α7nAChR ameliorates abdominal aortic aneurysm through inhibiting pyroptosis mediated by NLRP3 inflammasome. Acta Pharmacologica Sinica. Published online 25 February, 2022. doi:10.1038/s41401-022-00876-9. 2. 3, 4-Benzopyrene (Bap) aggravated abdominal aortic aneurysm formation by targeting pyroptosis in smooth muscle cells through ET-1 mediated NLRP3-inflammasome activation. International Immunopharmacology. Published online 29 August, 2023. doi:10.1016/j.intimp.2023.110851.)***. To further dissect this axis, future studies will employ VSMC-specific NLRP3 knockout mice to definitively test whether PDK4-mediated ROS requires NLRP3 to drive AAA, thereby addressing residual questions about alternative mechanisms.

12. PDK4 deletion in VSMCs reduces mitochondrial ROS production and suppresses VSMC pyroptosis. Is there a parallel or causal relationship between ROS and pyroptosis?

Response:

We thank the reviewer for this important question. Our data support a causal relationship between mitochondrial ROS and pyroptosis in VSMCs, rather than a mere parallel association. This conclusion is based on the following evidence:

1.Pharmacological inhibition of ROSusing MitoQ and NAC significantly reduced PDK4 overexpression-induced activation of NLRP3 inflammasome components (NLRP3, Cleaved Caspase1, Cleaved IL-1β, Cleaved IL-18) and pyroptosis executor (GSDMD, N-GSDMD) in MASMCs (**Figure 7B and S7A**).2.MitoQ and NAC treatment further reduced the expression of pyroptosis proteins in MASMCs isolated from *Pdk4*^{SMKO} mice compared to those from *Pdk4*$^{fl/fl}$ mice (**Figure 7C and S7B**), indicating that ROS acts upstream of pyroptosis. 3. ROS-induced activation of pyroptosis aligns with prior mechanistic studies ***(1. Control of gasdermin D oligomerization and pyroptosis by the Ragulator-Rag-mTORC1 pathway. Cell. Published online 21 July, 2021. doi:10.1016/j.cell.2021.06.028. 2. The metabolite α-KG induces GSDMC-dependent pyroptosis through death receptor 6-activated caspase-8. Cell Research. Published online 19 May, 2021. doi:10.1038/s41422-021-00506-9. 3. Nicotine promotes atherosclerosis via ROS-NLRP3-mediated endothelial cell pyroptosis. Cell Death & Disease. Published online 7 February, 2018. doi: 10.1038/s41419-017-0257-3.)***. These findings collectively demonstrate that PDK4-driven mitochondrial ROS production is a prerequisite for pyroptosis activation in AAA pathogenesis.

13. The association between pyroptosis and AAA in this article could be explained more clearly. Inhibition of NLRP3 inflammasome activation did not fully explain that PDK4-mediated pyroptosis promotes the AAA progression.

Response:

We thank the reviewer for this valuable comment and agree that the link between pyroptosis and AAA progression deserves clearer explanation.

First, previous studies have firmly established that NLRP3 inflammasome activation and pyroptosis contribute to AAA pathogenesis ***(1. Activating α7nAChR ameliorates abdominal aortic aneurysm through inhibiting pyroptosis mediated by NLRP3 inflammasome. Acta Pharmacologica Sinica. Published online 25 February, 2022. doi:10.1038/s41401-022-00876-9. 2. 3, 4-Benzopyrene (Bap) aggravated abdominal aortic aneurysm formation by targeting pyroptosis in smooth muscle cells through ET-1 mediated NLRP3-inflammasome activation. International Immunopharmacology. Published online 29 August, 2023. doi:10.1016/j.intimp.2023.110851.*)**. These reports support the biological relevance of pyroptosis in abdominal aortic aneurysm.

Second, to directly assess this pathway in our model, we treated PDK4-overexpressing mice with the NLRP3 inhibitor MCC950, and observed a significant reduction in AAA severity, as measured by aortic morphology, mortality rate, AAA incidence, aortic diameter, elastin degradation, and pyroptosis-associated protein levels (**Figure 10A-10H and S9F**).

Third, *in vitro* and transcriptomic analyses provide further mechanistic support: Transmission electron microscopy of Ang II-treated MASMCs confirmed that PDK4 deficiency substantially attenuated key pyroptotic hallmarks (cell swelling and membrane pore formation) (**Figure 6O**). This phenotypic evidence was corroborated by reanalysis of mouse single-cell RNA-seq data, which demonstrated elevated pyroptosis pathway activity specifically in AAA samples compared to controls using AUCell algorithm scoring (**Figure 6M**). Crucially, Gene Set Enrichment Analysis (GSEA) further demonstrated significant correlation between NOD-like receptor signaling and AAA progression in VSMCs (**Figure 6L**). Moreover, western blot analysis revealed that the protein levels of NLRP3, Cleaved Caspase1, Cleaved IL-1β, Cleaved IL-18, GSDMD, and N-GSDMD were significantly increased in human AAA tissues obtained from patients undergoing abdominal aorta replacement surgery compared to control aortic tissues from a non-aneurysmal segment of the same patient (**Figure 6P**). These findings further provide evidence that PDK4 promotes AAA via NLRP3-mediated pyroptosis.

Response to reviewer #1

Reviewer #1: The revised manuscript by Zhao et al. has improved substantially, and the authors have addressed most of the previous concerns. The inclusion of additional validation experiments and a more detailed description of the experimental design is appreciated. That said, several important issues remain that should be carefully considered.

1) The revised version appropriately cites prior work on the PDK/PDH axis (Griepke et al., 2025) and specifies that their approach, using VSMC-specific genetic models and a different AAA model, provides a more cell-specific and mechanistic perspective. However, although the authors argue in their reply that the identification of PDK4 rather than PDK1 may partly reflect model differences (PPE vs. AAV-PCSK9/Ang II), this point has not been explored or discussed in the revised manuscript. Acknowledging such model-dependent factors would help contextualize their findings and prevent overstating the specificity of PDK4 to AAA. Thus, the claim that this work "significantly advances the field" should be moderated.

Response:

We thank the reviewer for this insightful comment. We fully agree that model-dependent differences should be acknowledged to avoid overstating the specificity of PDK4. In the revised Discussion, we have added a dedicated paragraph explicitly addressing the distinct pathophysiological contexts of the PPE model versus the AAV-*PCSK9*^{DY}/Ang II model, and how these differences may underlie the divergent involvement of PDK1 and PDK4. This addition provides clearer context for our findings and better aligns our interpretation with prior literature.

Furthermore, in accordance with the reviewer's suggestion, we have moderated the strength of our original statement by revising the phrase "significantly advances the field" to a more balanced description of the contribution made by this study.

These changes can be found in the revised manuscript (**Page 23-24, lines 482-504**).

2) The authors identify PDK4 as the dominant isoform in AAA, but this conclusion should be tempered. Griepke et al., 2025 showed, using three independent human datasets and one murine dataset, that PDK1 was a dominantly expressed isoform in AAA. Hence, the current study's reliance on a single scRNA-seq dataset does not seem sufficient to disregard these prior findings. Moreover, as the authors have not experimentally excluded possible roles of other isoforms in their own system, the overall message of the manuscript aligns with a broader PDK/PDH axis role rather than a specific PDK4-driven mechanism.

Response:

We thank the reviewer for raising this important point and appreciate the reviewer's insightful perspective. We fully agree that the specificity of PDK4 should be interpreted with caution, particularly in light of prior reports suggesting a dominant role of PDK1

in AAA. To address this concern, and building upon our earlier Western blot analysis which showed no significant changes in PDK1, PDK2, or PDK3 protein levels in AAA tissues from the AAV-*PCSK9*^{DY}/Ang II model (**Figure S1G**), we have conducted additional experiments in the revised manuscript. In these new experiments, PDK1 knockdown in MASMCs did not attenuate Ang II–induced pyroptosis, as evidenced by comparable levels of NLRP3, Cleaved Caspase-1, Cleaved IL-1β, Cleaved IL-18, GSDMD, and N-GSDMD between control and PDK1-knockdown cells. These findings suggest that, within the context of our experimental system, PDK1 may not exert a dominant functional role.

At the same time, we acknowledge that these results do not exclude the possibility that PDK1 or other PDK isoforms may contribute to AAA development in different models or disease contexts. Isoform involvement within the PDK/PDH axis is likely context dependent, and comparative studies across multiple AAA models will be important for clarifying isoform-specific functions.

These additions have been incorporated into the revised manuscript (**Page 15, lines 309-315**) and in **Figure S6I**.

3) The new Oil Red O data (included in the rebuttal letter) showing comparable plaque burden between genotypes are important and should be fully integrated into the manuscript.

Response:

We thank the reviewer for this helpful suggestion. As recommended, the Oil Red O staining data have now been fully incorporated into the revised manuscript. These results demonstrate that plaque burden is comparable between genotypes, thereby supporting that the observed differences in AAA severity are not attributable to alterations in atherosclerotic lesion formation.

The detailed data have been added to the Results section (**Page 7, lines 150-151 and Page 8-9, lines 172-173**) and the corresponding point has also been discussed in the Discussion section (**Page 21, lines 441-443**). The images are presented in **Figure S3I and Figure S4I**.

4) Finally, a major concern remains, as the SMC-specific genetic targeting was conducted without the use of littermate controls. The explanation provided is understandable from a logistical standpoint but not sufficient to exclude potential effects of background genetic variability, which can substantially influence vascular phenotypes. In the absence of littermate comparisons, the authors should provide evidence of genetic background equivalence, for example, through verification such as microsatellite or SNP genotyping.

Response:

We thank the reviewer for raising this important point. We agree that ensuring genetic background uniformity is critical for the accurate interpretation of our results, especially in the absence of littermate controls. As the reviewer suggested, we have now

performed SNP genotyping to directly address this concern.

For this analysis, male *Pdk4*^{SMKO} (n = 5) and *Pdk4*$^{fl/fl}$ (n = 5) mice were selected from five independent litters within the same breeding colony used for AAA induction (one *Pdk4*^{SMKO} and one *Pdk4*$^{fl/fl}$ mouse per litter). Genomic DNA from these samples was subjected to SNP analysis across a panel of informative loci for the C57BL/6J background. The results showed that all tested *Pdk4*^{SMKO} and *Pdk4*$^{fl/fl}$ mice shared identical genotypes at all examined loci, consistent with a uniform C57BL/6J genetic background and with no evidence of mixed-background alleles. These findings support that the phenotypic differences observed in our study are attributable to VSMC-specific deletion of Pdk4 rather than underlying genetic background variability.

The detailed SNP results have been included in the revised manuscript (**Results: pages 7-8, lines 151-152; Methods: pages 37-38, lines 806-814**) and are summarized in **Table 1.**

Response to reviewer #2

Reviewer #2: The authors have made substantial revisions and addressed majority of previous concerns through additional experiments and improved figure presentation. Authors have included additional single-cell RNA-seq analysis and new experimental evidence, which together provide more mechanistic link. The inclusion of pharmacological inhibition data using DCA further supports the conclusion that PDK4 enzymatic activity contributes to AAA progression. Overall, the manuscript has been improved significantly and the conclusions are supported by solid evidence.

Minor Comments:

1.Add a scale bar to Figure 1L.

Response:

We thank the reviewer for pointing this out. A scale bar has now been added to Figure 1L in the revised manuscript.

2.The term "prevent AAA" may overstate the therapeutic effect. Consider consistently using terms such as "attenuate" or "reduce severity" to better reflect pharmacodynamic accuracy.

Response:

We thank the reviewer for this insightful suggestion. We agree that "prevent" may overstate the therapeutic effect observed. Therefore, we have revised the manuscript to consistently use the more precise term "attenuate" throughout, as recommended. These changes have been implemented in the revised manuscript (**line 41, page 2; line 85, page 4; line 395, page 19**).

3.The font size in the schematic illustration (Figure 10I) is relatively small; increasing the font size will improve readability.

Response:

We thank the reviewer for this helpful suggestion. The font sizes in the schematic illustration (**Figure 10I**) have been increased to improve overall readability in the revised manuscript.

Response to reviewer #3

Reviewer #3:

The manuscript written by Zhao Li and co-workers, showing the effects of PDK4 on smooth muscle cell pyroptosis and the progression of abdominal aortic aneurysm (AAA), as well as its relevant molecular mechanisms.

Overall, this paper has certain innovativeness. The author and his colleagues have made careful revisions to the paper. The questions raised by the reviewers have been responded to one by one. It can be considered that the author has completed the basic revision tasks.

However, the link between PDK4-mediated mitochondrial reprogramming and pyroptosis and the progression of AAA is to be investigated furtherly.

Response:

We thank the reviewer for this thoughtful and constructive comment. We agree that, although our study establishes a mechanistic framework linking PDK4 to mitochondrial dysfunction, mitochondrial ROS accumulation, TXNIP release, NLRP3 inflammasome activation, and VSMC pyroptosis, the full sequence of events driving AAA progression is not yet completely defined.

To address the reviewer's point, we have now expanded the Discussion to explicitly acknowledge these remaining mechanistic uncertainties. In particular, we note that it is still unclear whether PDK4-driven mitochondrial dysfunction promotes AAA primarily through NLRP3-dependent pyroptosis or whether additional ROS-responsive or inflammasome-independent pathways contribute. We further emphasize that future studies using more refined genetic and mechanistic approaches will be necessary to delineate the relative contributions of these pathways to PDK4-driven AAA development.

These clarifications and statements have been added to the revised Discussion (**Page 26-27, lines 563-574**).

Response to reviewer #1

Reviewer #1:The authors have responded appropriately to my previous comments, and the tone of the Conclusions has been notably improved. However, during the revision process, a new high impact study has been published demonstrating that VSMC-specific ablation of Pdk1 in the PCSK9-AAV/hyperlipidemia plus Ang II model confers protection against AAA (Liu et al., Circulation, PMID: 41487086), suggesting a mechanistic contribution distinct from that proposed here and in other studies.

On page 23, the authors state: "This divergence is likely influenced by the intrinsic pathophysiological differences between the two models⋯" and further argue that model-dependent inflammatory, metabolic, and hemodynamic contexts may preferentially engage different PDK isoforms. While this interpretation is reasonable, the recent evidence directly implicating PDK1 in VSMC-driven AAA development indicates that these findings should be more thoroughly integrated into the discussion.

Therefore, it would be important for the authors not only to cite this new study but also to reconcile it conceptually with their current framework. Rather than emphasizing individual PDK isoforms in isolation, the authors may consider framing alterations as changes in the PDK/PDH axis as the central mechanistic theme. Such an approach would better accommodate the emerging evidence that both PDK1 (in two studies) and PDK4 could be functionally relevant, influencing immunity, VSMCs and vascular disease.

I have no other comments.

Response:
We thank the reviewer for bringing this important new study to our attention and for the insightful suggestion to reframe our discussion around the PDK/PDH axis. We agree that this broader perspective better accommodates the emerging evidence implicating multiple PDK isoforms in AAA pathogenesis. Accordingly, we have now revised the Discussion section to:
1.Cite the new study by Liu et al. (*Circulation*, PMID: 41487086) demonstrating that VSMC-specific PDK1 deficiency protects against aortic aneurysm and dissection in the high-fat diet plus Ang II model.
2.Conceptually reconcile this finding with our own data by shifting the emphasis from individual PDK isoforms to the PDK/PDH axis as a central metabolic node. We now discuss how both PDK1 and PDK4 can be functionally relevant, likely operating in a context-dependent manner by influencing different cell types or responding to distinct pathological stimuli, yet converging on common downstream metabolic consequences

through the PDK/PDH axis.
These changes have now been incorporated into the revised Discussion (Page 23-24, lines 499-510). We thank the reviewer again for their valuable input, which has significantly strengthened our manuscript.
